# Ubiquitin Interacting Motifs: Duality Between Structured and Disordered Motifs

**DOI:** 10.3389/fmolb.2021.676235

**Published:** 2021-06-28

**Authors:** Matteo Lambrughi, Emiliano Maiani, Burcu Aykac Fas, Gary S. Shaw, Birthe B. Kragelund, Kresten Lindorff-Larsen, Kaare Teilum, Gaetano Invernizzi, Elena Papaleo

**Affiliations:** ^1^ Computational Biology Laboratory, Danish Cancer Society Research Center, Copenhagen, Denmark; ^2^ Department of Biotechnology and Bioscience, University of Milano-Bicocca, Milano, Italy; ^3^ Department of Biochemistry, Schulich School of Medicine and Dentistry, The University of Western Ontario, London, ON, Canada; ^4^ Structural Biology and NMR Laboratory and The Linderstrøm-Lang Centre for Protein Science, Department of Biology, University of Copenhagen, Copenhagen, Denmark; ^5^ Cancer Systems Biology, Section for Bioinformatics, Department of Health and Technology, Technical University of Denmark, Lyngby, Denmark

**Keywords:** molecular dynamics, peptide arrays, ubiquitin, short linear motifs, moonlight functions, intrinsic disorder

## Abstract

Ubiquitin is a small protein at the heart of many cellular processes, and several different protein domains are known to recognize and bind ubiquitin. A common motif for interaction with ubiquitin is the Ubiquitin Interacting Motif (UIM), characterized by a conserved sequence signature and often found in multi-domain proteins. Multi-domain proteins with intrinsically disordered regions mediate interactions with multiple partners, orchestrating diverse pathways. Short linear motifs for binding are often embedded in these disordered regions and play crucial roles in modulating protein function. In this work, we investigated the structural propensities of UIMs using molecular dynamics simulations and NMR chemical shifts. Despite the structural portrait depicted by X-crystallography of stable helical structures, we show that UIMs feature both helical and intrinsically disordered conformations. Our results shed light on a new class of disordered UIMs. This group is here exemplified by the C-terminal domain of one isoform of ataxin-3 and a group of ubiquitin-specific proteases. Intriguingly, UIMs not only bind ubiquitin. They can be a recruitment point for other interactors, such as parkin and the heat shock protein Hsc70-4. Disordered UIMs can provide versatility and new functions to the client proteins, opening new directions for research on their interactome.

## Introduction

Protein biochemistry relied for a long time on the paradigm that a protein’s function is tied to its three-dimensional structure. Over the past 20 years, several proteins or regions in proteins that do not fit within the structure-function paradigm have been reported (Wright and Dyson, 1999; Chen and Kriwacki, 2018; Milles et al., 2018). They are known as intrinsically disordered proteins (IDPs) or regions (IDRs). IDPs and IDRs lack stable tertiary contacts, are highly dynamic, pliable, and typically do not exhibit stable secondary structures. Proteins containing IDRs constitute 30–44% of eukaryotic proteomes (Perdigão et al., 2015). They attain multiple and chameleon conformations for interactions with different partners (Wright and Dyson, 2014; Bugge et al., 2020). Consequently, the modulation of the structural landscape of an IDP can result in opposing actions on different — or even the same — binding partners, making them elusive, but attractive targets to study (Metallo, 2010; Flock et al., 2014). IDPs and IDRs can also be involved in allosteric mechanisms with key roles in many processes, including modulation of protein-protein interactions and catalytic activities of enzymes (Ma et al., 2011; Li et al., 2017; Berlow et al., 2018; Guarnera and Berezovsky, 2019; Tee et al., 2020).

IDPs and IDRs often interact with binding partners through short stretches of conserved residues, called short linear motifs (SLiMs), embedded in otherwise non-conserved regions (Davey et al., 2012; Van Der Lee et al., 2014). The occurrence of two or more SLiMs in the same IDP/IDR can increase the interaction strength via avidity by multivalent interactions (Van Roey et al., 2014; Fung et al., 2018). Although individual SLiMs are short and mostly participate in transient interactions, they are essential to protein binding specificity and function (Bugge et al., 2020; Kumar et al., 2020).

Some functional motifs of proteins that were traditionally defined as helical elements have been recently reclassified as disordered SLiMs, such as the Bcl-2 Homology 3 motifs (Hinds et al., 2007; Aouacheria et al., 2015). Another well-known functional motif traditionally considered to have a high helical propensity (Scott et al., 2015) is the so-called Ubiquitin Interacting Motif (UIM) or ‘LALAL-motif’. UIMs are motifs of approximately 20 residues and were described for the first time in the 26S proteasome subunit PSD4/RPN-10 to bind ubiquitin (Young et al., 1998; Hofmann and Falquet, 2001), now representing the archetypal UIM in the families of ubiquitin binding domains (Scott et al., 2015). UIMs can be found, often in tandem or triplets, in a multitude of proteins involved in ubiquitination, ubiquitin metabolism, or that interact with ubiquitin-like modifiers (Buchberger, 2002). UIM binding partners are not limited to ubiquitin. As an example, ubiquitin-like proteins involved in autophagy feature an interface to recruit UIMs (Marshall et al., 2019; Sora et al., 2020). The UIM consensus motif is X-Ac-Ac-Ac-X-Φ-X-X-Ala-Φ-X-X-Ser-X-X-Ac-X, where Φ represents any hydrophobic residues (often Leu or Ile), Ac represents an acidic residue (Glu, Asp), and X loosely conserved positions (Hofmann and Falquet, 2001; Scott et al., 2015).

**FIGURE 1:**
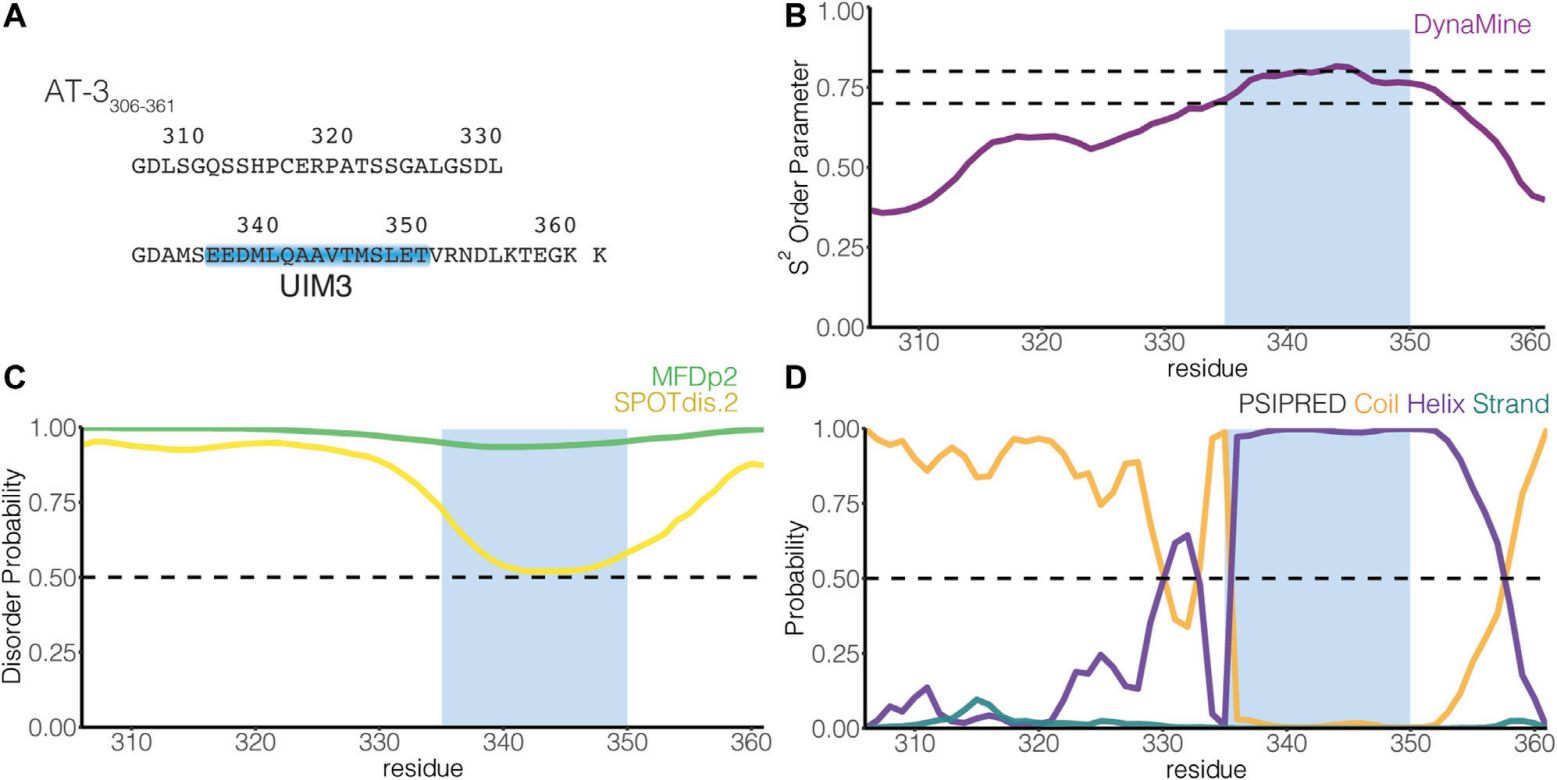
The C-terminus of the UIM3 isoform of ataxin-3 is predicted disordered and includes a UIM. **(A)** The figure illustrates the amino acid sequence of AT-3_306-361_. The blue box indicates UIM3 of AT-3 (residues E336-T350). **(B)** The panel shows the residue-wise prediction of the S^2^ order parameter in AT-3_306-361_ from *DynaMine* (purple). The dashed lines show the thresholds of S^2^ predicted scores to classify residues as ordered (S^2^ ≥  0.75), context-dependent (0.65 < S^2^  < 0.75), and disordered (S^2^   ≤ 0.65). **(C)** The panel shows the residue-wise prediction of disorder propensity in AT-3_306-361_ from the *Multilayered Fusion-based Disorder predictor v. 2.00* (MFDp2, green), and *SPOT-Disorder2* (SPOTdis.2, yellow) predictors. The dashed line shows the threshold of disorder probability to classify the residues as disordered ( ≥ 0.5) and ordered ( < 0.5). **(D)** The plot shows the residue-wise prediction of secondary structure propensity (Coil orange, Helix violet, Strand teal) from *PSI-PRED*. The dashed line shows the threshold of secondary structure probability for each structural class to classify the residues as coil, helix or strand ( ≥ 0.5). The light blue box indicates UIM3. The predictors report AT-3_306-361_ as a mainly disordered tract with propensity to order and helical structures in proximity of UIM3.

Among different UIMs, we focused our attention on the poorly characterized UIM within the C-terminus (residues 306–361) of the human ataxin-3 (AT-3). AT-3 is a multi-domain polyglutamine deubiquitinating enzyme used as a model system to study polyglutamine neurodegenerative diseases (Burnett et al., 2003; Carvalho et al., 2018; Invernizzi et al., 2012). AT-3 contains two UIM regions (UIM1 and UIM2) in the central part of the protein, surrounded by disordered regions (Burnett et al., 2003; Invernizzi et al., 2013; Masino et al., 2003; Sicorello et al., 2018, 2021). AT-3 also undergoes alternative splicing, and its isoforms differ in the C-terminus (Harris et al., 2010). Among the main isoforms, one isoform contains a third UIM, called UIM3 (Figure 1A (Goto et al., 1997; Bettencourt et al., 2010). The UIM3-containing isoform is widely expressed and appears to be the predominant form in the human brain (Ichikawa et al., 2001; Harris et al., 2010). Furthermore, AT-3 UIMs are involved in multivalent binding to the Ubl domain of the E3 ubiquitin ligase parkin (Bai et al., 2013; Aguirre et al., 2018). It has also been suggested that the three UIMs of AT-3 interact with the heat shock protein Hsc70-4 in *Drosophila melanogaster* (Johnson et al., 2020).

Recent advances in all-atom molecular dynamics (MD) simulations in terms of enhanced sampling (Abrams and Bussi, 2013; Spiwok et al., 2015; Bonomi et al., 2017; Sugita et al., 2019; Bussi and Laio, 2020) and physical models for disordered proteins (Best, 2017; Huang and MacKerell, 2018) offer a possibility to unveil heterogeneous conformational ensembles at the atomic level. The presence of multiple UIMs in the disordered C-terminus of AT-3 that are involved in the binding of different interaction partners makes this protein a good model to investigate the structural propensities of UIM using molecular dynamics simulations and chemical shifts from NMR.

We here report a study on the structural propensity and dynamics of the C-terminus of the UIM3-containing isoform of AT-3 (residues 306–361, AT-3_306-361_). We used two different methods to enhance the sampling of the MD simulations based on temperature exchange or bias along with selected collective variables. We also employed three different force fields (available at the time we performed the simulations) suitable to study disordered/unfolded states of proteins (Best and Mittal, 2010; Knott and Best, 2012; Lindorff-Larsen et al., 2012; Best et al., 2014). The simulation results for AT-3_306-361_ were then been compared to NMR data for other UIMs in solution (Sgourakis et al., 2010; Lim et al., 2011; Lange et al., 2012; Anamika et al., 2014; Shi et al., 2014; Wen et al., 2014; Sicorello et al., 2018) or to NMR data recorded in this work. In addition, we validated the simulations against previously published NMR chemical shifts of a construct of AT-3 including UIM3 (Bai et al., 2013).

We find that UIM-containing regions can account for both stable helical conformations and more disordered ones, which, in turn, are the more pliable toward a wider range of interactors beyond ubiquitin itself. Thus, our study provides a broader view on the ubiquinome through uncovering an enhanced structural heterogeneity within the groups of UIMs.

## Materials and Methods

### Bioinformatic Analysis

For the sequence-based prediction of secondary structure propensity, we used the PSIPRED predictor (Jones, 1999). We performed disorder prediction from the amino acid sequence, using DynaMine (Cilia et al., 2013), Multilayered Fusion-based Disorder predictor v. 2.00 (MFDp2, (Mizianty et al., 2013), and SPOT-Disorder2 (Hanson et al., 2019). MFDp2 is a meta-method that combines disorder probabilities predicted at residue- and sequence-level by MFDp and DisCon, respectively, and uses post-processing filters and sequence alignment. SPOT-Disorder2 combines long short-term memory with deep bidirectional neural networks to capture non-local and long-range interactions, integrating information from evolutionary profiles of aligned sequences. DynaMine allows high-quality predictions of protein backbone dynamics using an accurate NMR data set for training.

### Replica-Exchange Molecular Dynamics Simulations

REMD simulations were performed by GROMACS (Groninger MAchine for Chemical Simulation) using a conformation of the C-terminus of AT-3 (56 residues, 306–361, AT-3_306-361_) initially generated with Crystallography and NMR System version 1.3 (Brunger, 2007) as the starting structure. We further imposed a helical structure for the region E336-T357, according to the secondary structure prediction by PSIPRED, using MODELLER 9.14 (Eswar et al., 2007). In particular, we selected the model that lacked intermolecular side-chain contacts (defined as intramolecular contacts at a distance in sequence over three residues).

The models were soaked in a dodecahedron box of water molecules with periodic boundary conditions, with a minimal distance for the protein atoms from the box edges of at least 14 Å. We applied the Particle-Mesh Ewald method (Darden et al., 1993) with a 1.2 Å grid spacing. Van der Waals and Coulomb interactions were truncated at 12 Å. Na^+^ and Cl^−^ counterions were added to the system to neutralize the overall charge and to simulate a physiological ionic strength (i.e., 150 mM).

Each system was initially relaxed by 10,000 steps of energy minimization by the steepest descent method. The optimization step was followed by 50 ps of solvent equilibration at 300 K, while restraining the protein atomic positions using a harmonic potential. The systems were subsequently simulated for five ns at 300 K at a constant pressure of 1 bar (NPT ensemble) with coupling constants of 5 and 10 ps, respectively. From the NPT trajectories, we selected a conformation with the volume close to the average volume of the trajectory and used as the starting point for the subsequent NVT preparatory step at 300 K for 20 ns. The 64 initial conformations for REMD simulations were selected from different points (between 10 and 20 ns) along the NVT trajectory using the v-rescale thermostat (Bussi et al., 2007). Other details are reported in the parameter files in the GitHub repository.

In the temperature REMD scheme a number of different copies (replicas) of the system were simulated in parallel at different temperatures and exchanges of configurations are attempted periodically between pairs of replicas. The advantage of this method is that if the trajectory is temporarily trapped in a local minimum can exchange with a replica at a higher temperature and cross high-energy barriers. We carried out REMD simulations using 64 replicas, each replica for 50 ns for a collective simulation time of 3.2 μs. Each replica was run at a different temperature in the range 299–360 K. We selected the temperature spacing between each neighboring replica to ensure an exchange probability higher than 0.2. The replica-exchanges were attempted every ten ps.

### Well-Tempered-Metadynamics Simulations

The WT-metaD (Barducci et al., 2008) simulations were performed using GROMACS and the open-source, community-developed PLUMED library (Bonomi et al., 2009; PLUMED Consortium, 2019). In the WT-metaD simulations, the sampling of the free energy surface is enhanced by adding a history-dependent potential to a set of collective variables (CVs). Similar approaches have been applied to simulations of other intrinsically disordered proteins and peptides (Do et al., 2014; Palazzesi et al., 2015). We employed two CVs in our simulations, i.e., 1) the Cα radius of gyration, and 2) *alphabeta*, a CV that measures the similarity of each ψ dihedral angle of AT-3_306-361_ to a reference value of 0.7854 rad, which corresponds to *a*-helix. Gaussian potentials with an initial height of 0.12 kcal/mol were added to the time-dependent potential every two ps. We used an initial bias factor of four for rescaling the Gaussian height following the WT-metaD scheme. In addition, we used Gaussian widths of 0.2 and one for each CV, respectively. We collected one-μs WT-metaD simulations. We used an extended and disordered conformation of the peptide generated by Profasi (Irbäck and Mohanty, 2006) as the initial structure for the WT-metaD simulations.

### Force Fields and Water Models Employed in the REMD and WT-metaD Simulations

For the REMD simulations, we employed four different combinations of protein force fields and water models in our simulations: 1) Amber ff03w [ff03w (Best and Mittal, 2010)] with TIP4P/2005 (Abascal and Vega, 2005), 2) Amber ff03ws [ff03ws (Best et al., 2014)] with TIP4P/2005, in which the protein–water pair interactions have been modified to improve the description of disordered proteins, 3) CHARMM22* (Piana et al., 2011) with TIP3P (CHARMM22*_1_) (Jorgensen et al., 1983) or 4) TIPS3P (CHARMM22*_2_) (MacKerell, et al., 1998). WT-metaD was carried out only for ff03w, ff03ws, and CHARMM22*_2_.

### Analyses of the Simulations

The replica at 304 K was used for the analysis. To study the temperature distributions, we converted each replica to be continuous to the simulation time to follow each replica through the temperature space. We used DSSP (Kabsch and Sander, 1983) to estimate the helical content. We used MDAnalysis (Michaud-Agrawal et al., 2011) to calculate the root mean square deviation (RMSD) of UIM3 of AT-3_306-361_ with respect to the starting helical conformation. We considered the Cβ atom of A343 and the backbone (Cα, C, O, N) atoms of the residues E336-T350 of UIM3 for rigid body superposition and the RMSD calculation.

For the WT-metaD simulations, we reconstructed the one-dimensional free energy landscape from the deposited bias during the simulation with a stride value of 10,000. We extracted four ensembles of structures of AT-3_306-361_ from the CHARMM22*_2_ metadynamics trajectory with alphabeta values in the ranges of 1) 9–17, 2) 18–23, 3) 24–30, and 4) 31–34, respectively. On these ensembles, we estimated the propensity to helical structures using the DSSP dictionary (Kabsch and Sander, 1983) and including *a*-helix, *π*-helix and 3.10 helix in the analyses. We applied the MDplot R/CRAN package (Margreitter and Oostenbrink, 2017) to calculate a residue-wise persistence degree of helical secondary structures. On the ensembles selected from the CHARMM22*_2_ metadynamics trajectory, we used MDAnalysis to calculate the RMSD of UIM3 of AT-3_306-361_ (residues 336–350) with respect to: 1) the starting structure of AT-3_306-361_ used for the REMD simulations, 2) the experimental structure of yeast vps27 UIM1 [residue E259-E273, PDB entry 1Q0W (Swanson et al., 2003)], human proteasome subunit S5a UIM1 [residue A212-E226, PDB entry 1YX5 (Wang et al., 2005)] and UIM2 [residue E283-G297, PDB entry 1YX6 (Wang et al., 2005)], and mouse RAP80 UIM1 [residues E81-E95, PDB entry 3A1Q (Sato et al., 2009)] in complex with ubiquitin. We used the same subset of atoms for structural alignment and RMSD calculations, i.e., the Cβ atom of A343 and the backbone (Cα, C, O, N) atoms of residues E336-T350 of AT-3_306-361_.

### Comparison to the Available Chemical Shifts of AT-3_306-361_

To evaluate the REMD ensembles, we calculated the backbone chemical shifts as a function of the simulation time using PPM (Li and Brüschweiler, 2012) and compared them to the available NMR backbone chemical shifts for a construct of AT-3 including UIM3 [residues 194–361 (Bai et al., 2013)]. To compare the calculated backbone chemical shifts with the experimental ones, we used a reduced χ^2^ metric as previously described (Papaleo et al., 2018), using the Python package delta_cs (Sora et al., 2021). The reduced χ^2^ relates the squared deviation between the predicted and experimental value and normalized by the variance of the chemical shift predictor for each type of chemical shift and the total number of chemical shifts. Lower values of χ^2^_red_ metric indicate a better agreement between experimental and calculated chemical shifts.

### Protein Purification

We produced recombinant yeast ubiquitin in E. coli strain BL21 using a pMCSG7vector. Ubiquitin was expressed as a 6X histidine (6His)-TEV N-tagged fusion protein by the addition of 1 mM IPTG and incubation 5 h at 37°C. Cells were harvested, resuspended in a lysis buffer (50 mM Tris pH 8.0, 150 mM NaCl, 10 mM imidazole) plus protease inhibitor mixture (Roche), and disrupted by sonication. 6His-TEV-Ubiquitin was affinity purified with Ni Sepharose 6Fast Flow (GE Healthcare) and eluted with 20 mM Na2HPO4.2H2O, 0.5M NaCl, 500 mM imidazole, pH7.4.

For the construct of human AT-3 including residues 182–291 (AT-3_182–291_) we cloned it in frame with glutathione S-transferase (GST) in a pGEX-6P-1 (GE Healthcare LifeSciences, Little Chalfont, England) plasmid and expressed in E. coli BL21 Codon Plus strain (Stratagene, La Jolla, CA, United States) in auto-inducing growth minimal medium (Tyler et al., 2005). For the production of ^15^N labeled proteins, we included ^15^NH_4_Cl or (^15^NH_4_)_4_SO_4_ 1 g/l as the sole nitrogen source. For ^15^N^13^C labeled proteins, we added ^15^NH_4_Cl 1 g/l or (^15^NH_4_)_4_SO_4_ 1 g/l and substituted the carbon source with a solution of 0.4% ^13^C-glucose. Cells were harvested, resuspended in a lysis buffer (50 mM KH_2_PO_4_, 50 mM Na_2_HPO_4_, 300 mM NaCl, pH 7.4) to which DNAse (10 μg/ml, Sigma-Aldrich, St. Louis, MO, United States) and PMSF (1 mM) were added and then disrupted by sonication. We purified the soluble protein fractions by affinity chromatography with Glutathione Sepharose four Fast Flow resin (GE Healthcare Bio-Sciences, Uppsala, Sweden) and subsequently in-site cleaved with 60 units of PreScission Protease (HRV 3C Protease Sino Biological inc., Beijing, P.R.China) per ml of resin. We then further purified the eluted samples by size-exclusion chromatography on a Superdex 75 10/300 GL column (GE Healthcare LifeSciences, Little Chalfont, England) in PBS buffer, pH 7.4, 150 mM NaCl.

### Peptide Array

We purchased peptide arrays from Intavis and modified the procedures for blocking and probing the arrays from (Frank and Dubel, 2006). Briefly, the peptide array was re-hydrated through incubation in 100% ethanol and transferred in TBS (137 mM NaCl, 2.7 mM KCl, and 50 mM Tris, pH 7.0) for 5 min at room temperature. The blocking was performed by incubating the membrane 4°C overnight in TBS with 5% nonfat dry milk (MBS). Membranes were then incubated with 10 ml MBS with 2 mg/ml of 6His-TEV-Ubiquitin for 3 h at room temperature. The peptide array was then rinsed with a blocking buffer and then incubated with anti-6His antibody (Sigma Aldrich C6594) diluted 1:1,000 in the blocking buffer for 2 h at room temperature. The membrane was washed in Tween TBS 3 times and then incubated 1 h at room temperature with the secondary antibody (anti-mouse AP from Immunstar kit 170–5010).

### NMR Spectroscopy of AT-3_182-291_

NMR samples were prepared by dissolving the purified protein in 90% PBS buffer, pH 7.4, 150 mM NaCl and 10% D_2_O with4,4-dimethyl-4-silapentane-1-sulfonic acid (DSS) added as internal calibration standard. Protein concentrations were from 0.5 to 1 mM in a volume of 400 μl. Assignment of backbone chemical shifts was performed on a 0.5 mM ^13^C, ^15^N AT-3_182-291_ sample and ^1^H, ^15^N-HSQC spectrum and the following triple resonance spectra were recorded, HNCA, HN(CO)CA, HNCO, HN(CA)CO, CBCA(CO)NH, CBCANH, CC(CO)NH and H(CCO)NH (all pulse programs from Agilent BioPack) at 25 °C on a Varian Unity Inova 750 and 800 Mhz instruments. NMR data were processed by NMRPipe (Delaglio et al., 1995) and analyzed using CCPNMR (Skinner et al., 2016). The chemical shift assignment for AT-3_182-291_ is deposited in the Biological Magnetic Resonance Bank (BMRB) with entry 50888.

### Prediction of Secondary Structural Propensity From NMR Chemical Shifts

We downloaded NMR chemical shift data from the Biological Magnetic Resonance Bank (BMRB) for STAM1 [BMRB entry 17065 (Lim et al., 2011)], STAM2 [BMRB entry 18403 (Lange et al., 2012)], vps27 [BMRB entry 16114 (Sgourakis et al., 2010)], USP25 [including UIM1 and UIM2, BMRB entry 19111 (Shi et al., 2014)], RAP80 ([including UIM1 and UIM2, BMRB entry 19774 (Anamika et al., 2014)], USP28 [BMRB entries 18560 and 19077 (Wen et al., 2014)], and AT-3 [including UIM1, UIM2, and UIM3, BMRB entry 27380 (Sicorello et al., 2018)]. Furthermore, we included in the analyses the chemical shifts for AT-3_182-291_ (including UIM1 and UIM2 of AT-3) from experiments performed in this study, along with previously published data for an AT-3 construct including the UIM3 residues 194–361 (Bai et al., 2013). We used the backbone chemical shifts from these NMR sets to predict the secondary structure propensity by δ2D (Camilloni et al., 2012).

### Helical Wheel Projections

We calculated the helical wheel projections of UIMs of the selected proteins by the freely available NetWheels web-based application (Mól et al., 2018).

## Results

### Conformational Ensemble of AT-3_306-361_ in Solution

We used NMR data for AT-3 UIM3 from a previous publication (including residues 194–361) (Bai et al., 2013). MD simulations of such a long and disordered region are challenging, due to several conformational transitions to sample and a large number of degrees of freedom involved. We thus focused on a shorter construct for MD simulations, i.e., AT-3_306-361_.

We employed two different methods to characterize the conformational ensemble of AT-3_306-361_ in solution, i.e., REMD and WT-metaD. These methods provide the possibility to enhance the sampling of the conformational space in MD simulations while keeping a description of both the protein and the solvent at the atomic level. We also evaluated the influence of different force-field descriptions for both the protein and the solvent: Amber ff03w-TIP4P/2005, Amber ff03ws-TIP4P/2005, CHARMM22*-TIP3P, CHARMM22*-TIPS3P (indicated as ff03w, ff03ws, CHARMM22*_1_, and CHARMM22*_2_, respectively) to assess the reproducibility of the result and identify force-field dependent properties. These approaches enabled us 1) to address if AT-3_306-361_ is stable or not in a helical conformation in solution, 2) to estimate the population of the helical conformations and compare them to the available experimental information on a variant of AT-3 (residues 194–361) characterized by NMR and on other known UIMs that have been similarly studied by solution NMR (see *Materials and Methods*) or recorded by us in this work ( AT-3_182-291_), 3) to identify conformations that resemble ubiquitin-bound states in the ensemble of the free AT-3_306-361_ region through the comparison of our ensembles to the experimentally known ubiquitin-bound UIM structures of other proteins.

### Low Structural Propensity and Heterogeneous Helical Formation in the Free State of AT-3_306-361_ Domain in Solution

UIMs are thought to assume an α-helical structure also in the absence of ubiquitin binding (Hofmann and Falquet, 2001; Scott et al., 2015). Nevertheless, many investigations on UIMs focus on characterizing the binding with ubiquitin, making it unclear if UIMs present transient propensity to disordered conformations in their free state, a typical trait of SLiMs (Davey et al., 2012; Van Roey et al., 2012, 2014). In AT-3_306-361_, UIM3 spans residues E336-T350 [(Donaldson et al., 2003), Figure 1A]. To identify inherent structural properties, we used four sequence-based methods to predict disorder or secondary structure propensity (Nielsen and Mulder, 2019). Overall, the predictors showed a disordered state for the AT-3_306-361_ region with propensity to order and helicity around UIM3 (Figures 1B–D).

**FIGURE 2:**
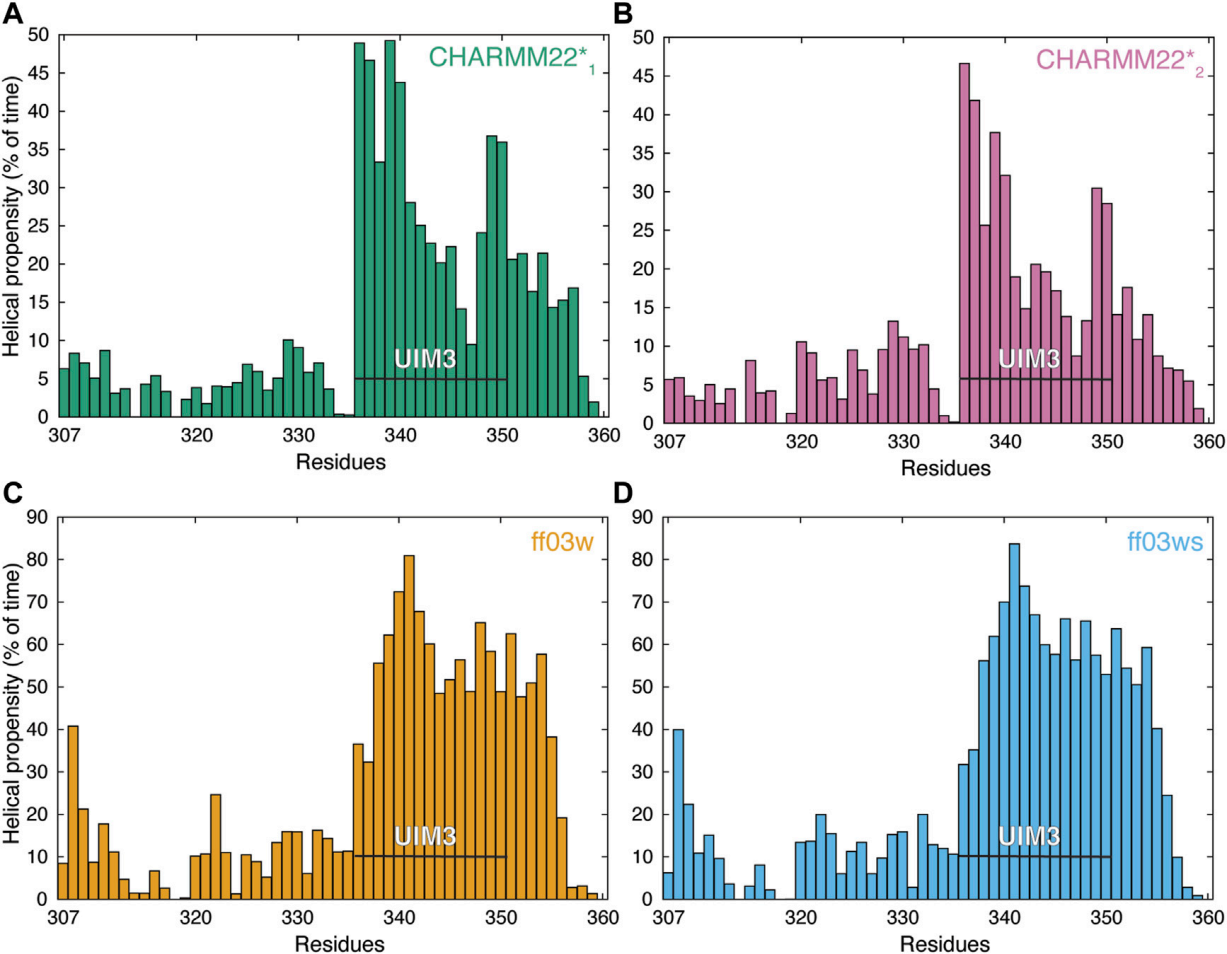
AT-3_306-361_ in the free state assumes both helical and non-helical conformations. The panels show the per-residue helical content of each replica at 304 K from the REMD simulations of the AT-3_306-361_ for each combination of protein force fields and water models: **(A)** CHARMM22*-TIP3P (CHARMM22*_1_, green), **(B)** CHARMM22*-TIPS3P (CHARMM22*_2_, pink), **(C)** Amber ff03w-TIP4P/2005 (ff03w, orange), and **(D)** Amber ff03ws-TIP4P/2005 (ff03ws, blue). The residues of UIM3 (residues 336–350) are highlighted by the black bars. The REMD simulations with ff03w and ff03ws show high helical content for UIM3 while the CHARMM22*_2_ simulation reports more disordered and heterogeneous conformations of UIM3.

We subsequently modeled this region as an α-helix in the starting structure for the REMD simulations. In the REMD simulations, the UIM3 region assumed both helical and non-helical conformations (Figure 2). The REMD simulations with ff03w and ff03ws showed higher helical content for UIM3 (∼60% and 56%, respectively) than with CHARMM22*_1_, and CHARMM22*_2_ (∼31% and 25%, respectively) (Figure 2). In the case of CHARMM22*_2_ simulation, we observed a more disordered ensemble for UIM3, with helical content < 20% in the region Q341-L348.

**FIGURE 3:**
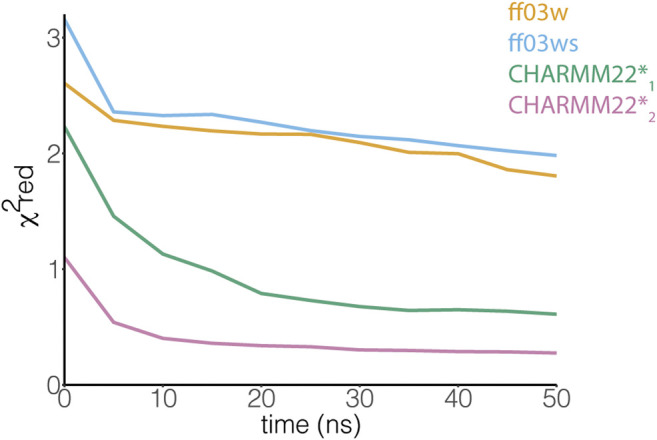
The ensemble of AT-3_306-361_ from the CHARMM22*_2_ REMD simulation better resembles the experimental chemical shifts. The plot shows the comparison between experimental Cα chemical shifts and calculated Cα chemical shifts from the REMD simulations. Similar results have been achieved using the other backbone (N, HN, C, O, Hα) and Cβ chemical shifts. Among the protein force fields and water models tested in this study, the CHARMM22*_2_ REMD simulation shows a better agreement with the experimental NMR measurements.

An NMR backbone chemical shift assignment for AT-3_306-361_is available (Bai et al., 2013). We used this set of experimental data to evaluate the REMD structural ensembles. In particular, we calculated backbone chemical shifts as a function of the simulation time and compared these to the experimental values. The calculated chemical shifts from our simulations converged after only 5 ns of REMD simulations (Figure 3). They are in agreement with the experimental values with low χ^2^_red_ values of CHARMM22* simulations, but not in the ff03w/ff03ws simulations (Figure 3). In ff03w/ff03ws, the simulations converged to structures that are unlikely to resemble the ones observed by solution NMR, probably due to the high helicity sampled by these trajectories.

**FIGURE 4:**
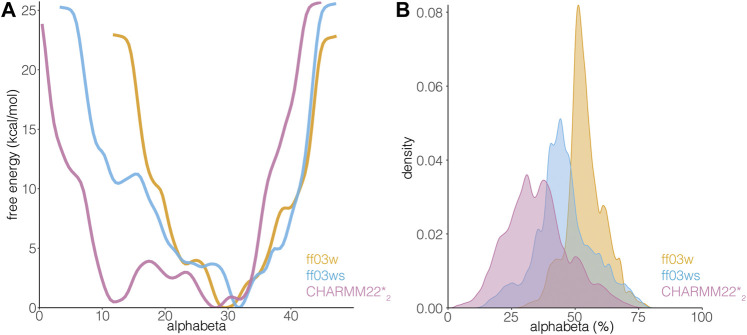
AT-3_306-361_ is characterized by a disordered ensemble with a low structural propensity and heterogeneous helical states. **(A)** The plot shows the one-dimensional free energy profile associated with the collective variable alphabeta for the ff03w (orange), ff03ws (blue), and CHARMM22*_2_ (pink) metadynamics simulations. **(B)** The plot shows the distribution of alphabeta values expressed as a percentage, i.e., alphabeta values divided by the total number of torsional angles considered. Ff03w and ff03ws show overstabilization of helical conformations while CHARMM22*_2_ better describes the disordered ensemble of AT-3_306-361_ in the free state.

The differences in the sampling of helical structures in the REMD simulations with different force fields could be ascribed to either force field differences or limitation of the conformational sampling. Since we started from an α-helical conformation the simulation time might not have been sufficient, even with the temperature replica-exchange, to allow the protein to exhaustively explore the conformational space in the different force-field simulations. Thus, to be able to discriminate between these two scenarios, we applied another method for enhanced sampling, based on metadynamics. In particular, we carried out simulations with WT-metaD, which should allow a more extensive exploration of the conformational space by using the Cα radius of gyration and *alphabeta* as collective variables (CVs) to bias the systems*.* Alphabeta is a collective variable in which we measured similarity for all ψ dihedral angles of the peptide to the ψ dihedral angles of an ideal α-helix (Figure 4). It is a suitable CV to enhance the sampling of disordered regions which might have local propensity for helical structures (Granata et al., 2015). The alphabeta estimated by the three different force fields were different with the CHARMM22*_2_ simulation providing more disordered conformations (i.e., alphabeta between 8 and 15 residues in Figure 4). As also observed in the REMD simulations, the ff03w ensemble was characterized by a higher helical content, suggesting that the difference observed is not necessarily related to limitations in the sampling or initial conformation, but to differences in force field parameters. In this context, overstabilization of helical conformations with ff03w has been observed also in other studies (Huang and Mackerell, 2014). The modification of the ff03ws force field with more balanced interactions between the protein and the solvent (Best et al., 2014) partially mitigates this effect, providing an ensemble of structures with a lower helical propensity, including also disordered states corresponding to the ones observed for CHARMM22*_2_ (Figure 4).

The transition between more ordered and disordered states is favored in the description provided by CHARMM22*_2_ (with difference in free energy of 1.5 kcal/mol). In the ff03ws simulation, the two states were separated by a barrier of more than 8 kcal/mol. The intrinsic preference for helical conformations of ff03w/ff03ws is likely to make the sampling of disordered states more challenging even with an enhanced sampling approach. The high energy barriers observed are thus likely to be due to limitations of the sampling. Longer simulations or other enhanced sampling approaches could help to obtain free energy profiles with a larger number of order to disorder transitions for this peptide and ff0ws (Bussi and Laio, 2020).

In summary, AT-3_306-361_ is characterized by a disordered ensemble, which is better described by CHARMM22*_2_ among the force fields tested in this study. The UIM3 region of AT-3_306-361_ can interconvert between more disordered and partially helical states.

### Bound-Like Conformations in the Unbound AT-3_306-361_ Ensemble in Solution

**FIGURE 5:**
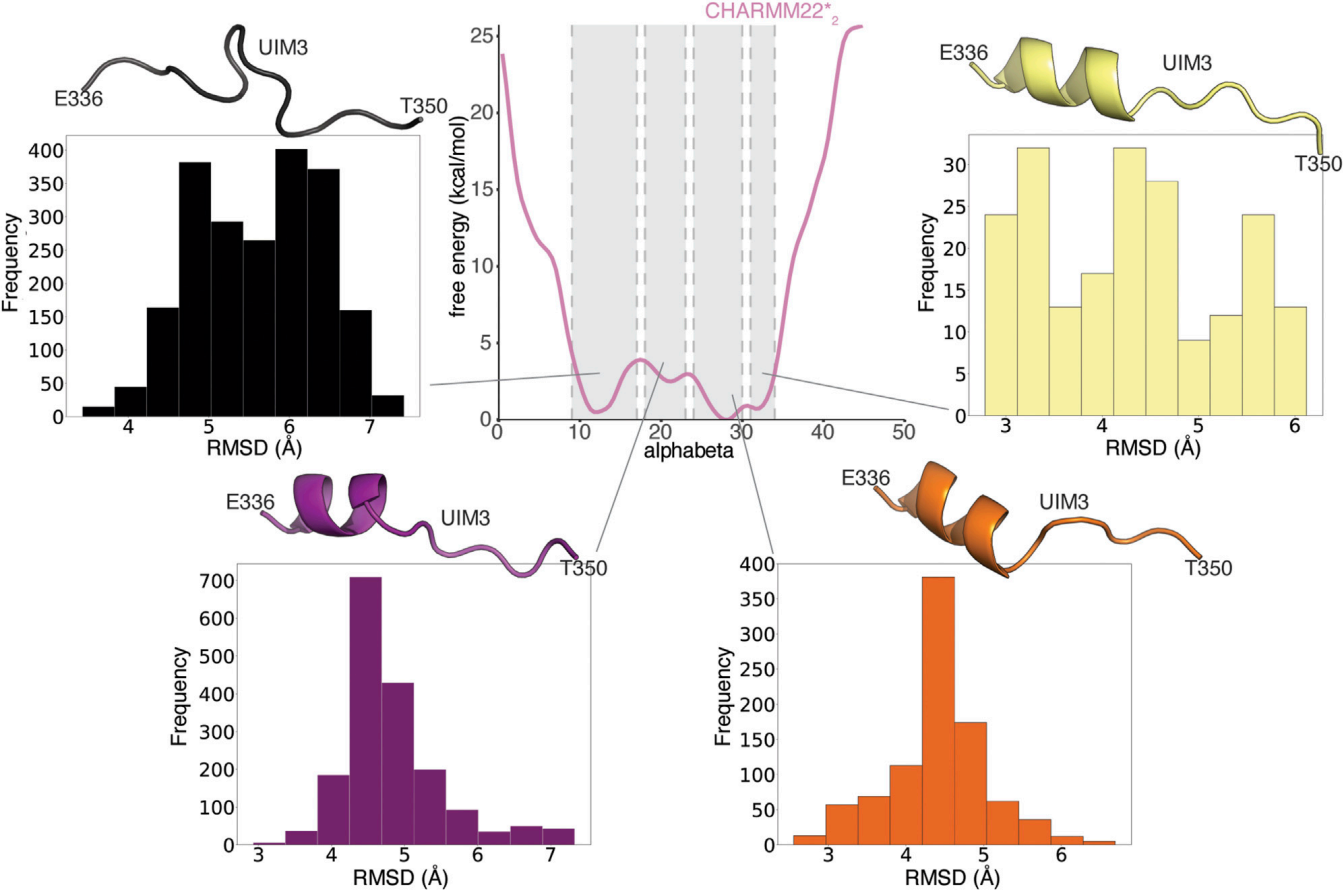
UIM3 of AT-3_306-361_ interconverts between disordered conformations and partially helical states in the free ensemble. The central panel shows the mono-dimensional free energy profile associated with alphabeta for CHARMM22*_2_ (pink) metadynamics simulation. The four side panels show the RMSD calculations of UIM3 structures from the CHARMM22*_2_ metadynamics trajectory, using as a reference the starting structure of AT-3_306-361_ for the REMD simulations, in which UIM3 is modeled as an α-helix. We used the Cβ atom of A343 and the backbone (Cα, C, O, N) atoms of the residues E336-T350 of UIM3 for rigid body superposition and the RMSD calculation. We calculated the RMSD of four ensembles of structures of UIM3 with alphabeta values in the ranges of: i) 9–17 (black), ii) 18–23 (purple), iii) 24–30 (red), and iv) 31–34 (yellow), respectively. The cartoon representations show the structures of UIM3 with the lowest RMSD in each of the four subsets. We identify partially helical states of UIM3 associated with alphabeta values in the range of 24-30 and 31–34.

Both ordered and disordered proteins often sample bound-like states that could be important for their binding, which may sometimes occur via a mechanism known as conformational selection (Davey, 2019). We, therefore, asked if this was also the case for UIM3 of AT-3_306-361_. To this end, we compared conformations from the CHARMM22*_2_ WT-metaD simulation with the starting structure of AT-3_306-361_ for the REMD simulations, in which UIM3 is modeled as a well-folded *a*-helix (Figure 5). We identify partially folded states of UIM3 (around 3 Å of RMSD), characterized by helical conformations in the N-terminal region of the motif (residues 336–344) (Figure 5) and alphabeta values in the range of 24-30 and 31–34 residues (Supplementary Figure S1). We observed that the region with the highest propensity to fold to helix corresponds to the UIM3 region (residues 336–344). This accounts for approximately 20% of the structures from the entire WT-MetaD. We also observed a minor helical propensity in other regions of the peptide, especially around residues 320–334 (less than 10% of the structures from the metaD).

We performed the same RMSD analysis on the replicas at 304 K from the REMD simulations (Supplementary Figure S2). In contrast with the results from WT-metaD, the REMD simulations tend to show a group of fully helical conformations of UIM3 (which are a minority of the frames in the CHARMM22^*^_2_ simulations, i.e. ∼ 3% of the frames) (Supplementary Figure S2). These analyses suggest that the REMD simulations provide a limited sampling and they are still biased by the initial helical conformation of UIM3. We thus relied on the WT-metaD results for the following analyses.

**FIGURE 6:**
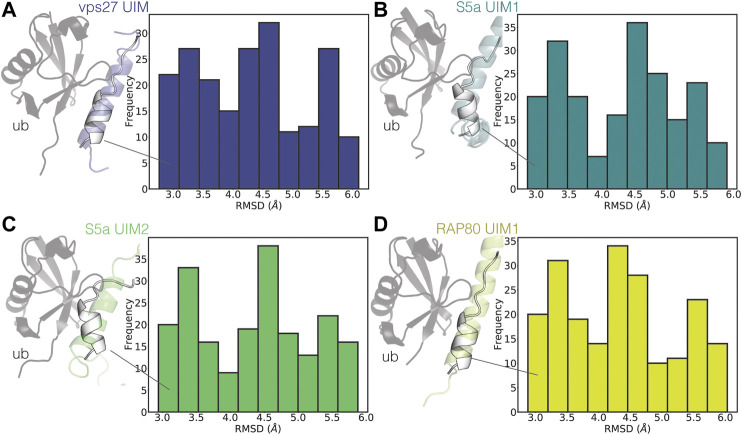
UIM3 of AT-3_306-361_ samples states partially resembling ubiquitin-bound conformations. The plots show the RMSD calculations of UIM3 of AT-3_306-361_ from the CHARMM22*_2_ metadynamics trajectory, using as a reference the experimental structure of the UIMs of other proteins in complex with ubiquitin **(A)** VPS27 UIM (PDB entry 1Q0W, blue), **(B)** S5a UIM1 (PDB entry 1YX5, teal), **(C)** S5a UIM2 (PDB entry 1YX6, green), **(D)** RAP80 UIM1 (PDB entry 3A1Q, yellow). We calculated RMSD for the ensembles of structures of UIM3 with alphabeta values in the range of 31-34. The cartoon representations show the structures of the experimental complexes, with the ubiquitin monomers shown as gray cartoons. The white cartoon representation shows the conformations of UIM3 (residues 336–350) from the CHARMM22*_2_ metadynamics simulation with the lowest RMSD to each experimental structure.

To identify the presence of bound-like states, we then compared the partially helical conformations of UIM3 of AT-3_306-361_ from the CHARMM22*_2_ WT-metaD simulation with the experimental structures of ubiquitin in complex with UIMs from other proteins (Figure 6).

UIMs are generally in folded helical conformations when bound to ubiquitin (Fisher et al., 2003; Swanson et al., 2003). We identified states of UIM3 partially resembling the bound conformations of other UIMs, characterized by RMSD around 3 Å with respect to the experimental complexes (Figure 6).

### Disordered UIMs With Low Helical Propensity: A More General Class of UIMs

**FIGURE 7:**
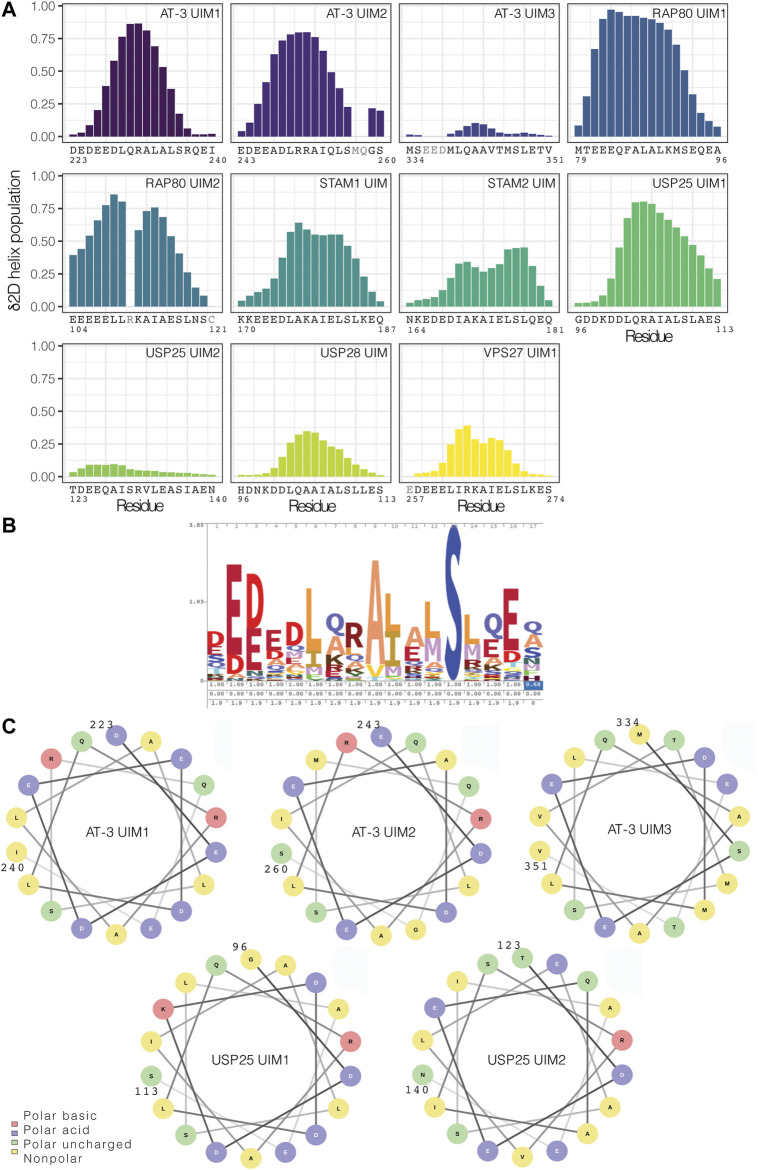
UIMs in the free state can vary from highly helical conformations to disordered counterparts. **(A)** Helical content for the UIMs predicted from chemical shifts by δ2D. We used nine sets of released chemical shifts of UIMs in the free state in solution from the BMRB database, including AT-3 UIM3, STAM1 UIM, STAM2 UIM, USP28 UIM, USP25 UIM1, and UIM2, and RAP80 UIM1 and UIM2. We also used a set of released chemical shifts of VPS27 UIM1 in fusion with the ubiquitin. In addition, we used the NMR chemical shifts that we recorded for AT-3_182-291_ UIM1 and UIM2. We highlighted in gray the residues for which there are not enough chemical shifts to run the prediction with δ2D. UIMs have a wide range of predicted helical content. **(B)** Consensus sequence for UIMs in the PFAM database. **(C)** Helical wheel representation of AT-3 UIM1, UIM2, and UIM3, and of USP25 UIM1 and UIM2.

To discriminate if the low occurrence of a helical conformation in solution is a distinctive trait of UIM3 or a more common property of other UIMs, we searched the NMR database BMRB for chemical shift data on other UIMs in solution. We identified nine sets of released chemical shifts for AT-3 (including UIM1, UIM2, and UIM3), STAM1, STAM2, USP28, USP25, and RAP80 (holding two UIMs each) (Supplementary Figure S3). We also used a set of chemical shifts of VPS27 UIM1 in fusion with ubiquitin (Supplementary Figure S3). In addition, we recorded NMR experiments to collect backbone and side-chain chemical shifts for UIM1 and UIM2 of AT-3 in solution, using AT-3_182-291_. From the chemical shifts, we predicted the secondary structural propensity by δ2D (Figure 7A and Supplementary Figure S3). We observed UIMs with high helical content, such as UIM1 and UIM2 of AT-3, UIM1 and UIM2 of RAP80, and UIM1 of USP25 (average δ2D helix population higher than 0.3), and low helical content, as in the case of UIM3 of AT-3 and UIM2 of USP25 (average δ2D helix population lower than 0.1). USP28 has also a lower helical content compared to other UIMs suggesting a heterogeneous ensemble of conformations. We observe a lower helical content in the case of VPS27 UIM in fusion with ubiquitin, possibly suggesting that in the bound state some UIMs could retain disorder. Our NMR data of AT-3_182-291_ are in agreement with previously published sets of chemical shifts of AT-3, showing high helical content for UIM1 and UIM2 (average δ2D helix population above 0.3 for UIM1 and 0.4 for UIM2 in all datasets) (Supplementary Figure S3). Furthermore, our analysis on the two sets of chemical shifts of UIM3 shows low helical content for both of them (average δ2D helix population under 0.1 for each set) (Figure 7A and Supplementary Figure S3), confirming the presence of disordered conformations.

Our results overall indicate that UIMs in the unbound state can span not only fully-formed helical conformations but also rather disordered counterparts. Moreover, a peptide SPOT arrays in which we studied the interaction of some representative UIMs with recombinant yeast ubiquitin shows that both disordered UIMs (AT-3 UIM3 and USP25 UIM1 and 2) and helical UIMs (STAM1, STAM2 and, AT-3 UIM1) interact with ubiquitin (Supplementary Figure S4). Thus, as all other UIMs tested are folded in the unbound state, these data suggest that a disordered UIM is not a barrier to bind ubiquitin.

To address if different classes of UIMs can be derived based on sequence and disorder, we compared the UIM3 sequence to other known UIMs and with the consensus sequence deposited in the Pfam database (entry PF02809) (Figure 7B). The 336–350 region of the C-terminus of AT-3 presents the typical signature of a UIM with conserved residues, such as L340, A343, S347, acidic residues in the N-terminal part of the motif (E336-D338), and the pattern of hydrophobic residues (Figure 7B **)**. Moreover, in comparison to other UIMs, we should notice that suboptimal residues for helical formation are observed in the UIM3 sequence in comparison to other UIMs. For example, V344 and T345 are both at low helix propensity (Nick Pace and Martin Scholtz, 1998) and are localized in the region of UIM3 where the helix tend to break in some of the simulation frames (see above). Furthermore, USP25 has a valine replacing the invariant alanine of the motif and an insertion of an arginine in the N-terminal region of the motif which might alter the helical pattern.

To further identify if this is a common signature to other disordered UIMs, we carried out a helical wheel analysis of AT-3 UIM1, UIM2, UIM3, and of UIMs previously investigated (USP25 UIM1 and UIM2 Figure 7C). The analysis shows that when UIM3 assumes a helical conformation T345 is located on the face of the helix with one of the acidic residues (i.e., E337) that is conserved in UIMs. Moreover, T350 is located at the same face of the helix as A343 and S347; two residues that are strictly conserved in all UIMs since they are involved in the interaction with ubiquitin (Fisher et al., 2003). For the disordered UIM2 of USP25, a similar valine and isoleucine, two beta-branched amino acids, break the helicity. This means that disordered UIMs may carry similar sequence properties that allow for their identification. Our analysis and simulations overall suggest that the location of suboptimal residues, especially threonine and valine, coul be related to the low propensity to populate stable helical conformations in solution. A search based on regular expression with the motif x-[ED]-[ED]-[ED]-x-[AILVFWMP]-x-x(1,2)-[AVP]-[VPL]-[EDVNTCGPH]-x-S-x-x-[EDTVNCGPH]-x against the sequences associated with the Pfam entry PF02809 highlights other 1,614 hits in 626 sequences of UIMs with likelihood to be (partially) disordered in the unbound state (against 172 hits found in a randomized dataset from Uniprot). Among the disordered UIM candidates we find UBP37 from different species (residues 704–720), which feature patterns similar to USP25. The motif search suggests that disordered UIM could be a common class of SLiMs (see Supplementary Text S5).

## Discussion

We focused on the structural characterization of the conformational ensemble of a functional motif that has been classically defined for its helical conformation and originally associated with the binding of ubiquitin; the Ubiquitin Interacting Motif (UIM). We showed that the motifs could be more degenerate and account for both helical and more disordered members, a diversity that has functional implications. With an approach integrating simulations and experimental biophysical data, we showed that a C-terminal UIM of AT-3 is embedded in an intrinsically disordered region, bearing a predominantly disordered UIM of which a small fraction of the ensemble has helical propensity in the N-terminal region. An unbound ensemble as the one depicted by WT-metaD might suggest that a combination of conformational selection (i.e., pre-formed regions of the UIM in helical conformation) and folding upon binding could be in place for UIM3. The occurrence of one or the other mechanism might also depend on the nature of the client protein and help to confer UIM3 promiscuity toward different partners of interaction, an effect that can be further tuned by post-translational modifications. These mechanisms will require future investigations in which kinetics can be accounted for.

We also discovered that the disordered nature of UIM3, and low helical propensity in the free state, is not an isolated example, as shown by the analysis of the NMR data of USP25 UIM2, USP28 UIM, and VPS27 UIM1.

In our work, all the UIMs tested for binding with ubiquitin are either folded or disordered in the unbound state. These data suggest that a disordered UIM is not a barrier to bind ubiquitin. NMR measurements on UIM3 still suggest that the binding could be of lower affinity than what observed for helical UIMs (Bai et al., 2013), supporting a more pliable partner toward a different range of client proteins, at the cost of larger entropy loss in binding ubiquitin.

UIMs not only bind ubiquitin but can also be interfaces to recruit other proteins, such as the case of UIM and parkin. Proteins including disordered UIMs can have additional diversity in their protein-protein interactions and cellular functions. For example, it has been suggested by mass-spectrometry and co-immunoprecipitation assays that AT-3 isoforms differ in their interaction with other proteins (Weishäupl et al., 2019). Post-translational modifications are likely to add an extra level of regulation, and they could modulate the helical propensity of disordered UIMs and their preferences for binding partners, as seen for other IDRs (Mylona et al., 2016; Hendus-Altenburger et al., 2017; Csizmok and Forman-Kay, 2018; Marceau et al., 2019). For example, UIM3 is sumoylated at K356 and this enhances affinity for the binding to ATPase p97 to transfer proteins for proteasomal degradation (Almeida et al., 2015). Further experimental and computational studies of these disordered UIMs here identified and their post-translational regulation or the study of UIM from other proteins could contribute to clarify the structural and sequence features of disordered and folded UIMs, along with their connection with certain binding partners and biological functions.

Our analysis and simulations overall suggest that the location of suboptimal residues for helix formation, especially beta-branched residues as threonine, valine and isoleucine could play a role in gearing the low propensity of UIMs to populate stable helical conformations in solution and provide a gateway to multispecificity.

UIMs are not the only example of such structural duality. Some regions of proteins that were traditionally defined as helical elements, due to their conformation in the bound states, have been reclassified as disordered SLiMs, as in the case of the Bcl-2 Homology 3 motifs (Hinds et al., 2007; Aouacheria et al., 2015). The presence of this emerging higher structural variability into different classes of SLiMs is a shift in our view of functional protein regions that can account for both helical and more disordered counterparts. A better understanding of the structural diversity within each class of functional motifs could open new directions to understand biomolecular interactions and their specificity or flexibility toward multiple partners of interaction. SLiMs with disorder propensity and a more versatile interface could enhance the pool of functions of a certain protein, for example increasing the number of potential binding partners, allowing the protein to act at the cross-road among different biological processes, or allow for a fine regulation by post-translational modifications.

## Data Availability

All the scripts, raw data, and outputs associated with this publication are available at the repository on GitHub https://github.com/ELELAB/disoUIM and on OSF https://osf.io/zfy9s/.
