# Supplementary material for: Ubiquitin Interacting Motifs: Duality Between Structured and Disordered Motifs
Source: Front Mol Biosci. 2021 Jun 28;8:676235. doi: 10.3389/fmolb.2021.676235 (PMC8273247; doi:10.3389/fmolb.2021.676235)
Supplement: Supplementary file 1 [file Table5.pdf]

ScanProsite tool

Output format: Table

Hits for USERPAT1{x-[ED]-[ED]-[ED]-x-[AILVFWMP]-x-x(1,2)-[AVP]-[VPL]-[EDVNTCGPH]-x-S-x-x-[EDTVNCGPH]-x} motif on all MsFJlCjlEK custom database database sequences :

found: 1614 hits in 626 sequences

Graphical view (graphical view with feature detection)
shaded alignment of hits

A0A060VR78_ONCMY 681 697 USERPAT1 . . .
nDEEmLaa.VLEmSrqDa
A0A060VR78_ONCMY 681 697 USERPAT1 . . .
nDEEmLaa.VLEmSrqDa
A0A060VR78_ONCMY 681 697 USERPAT1 . . .
nDEEmLaa.VLEmSrqDa

A0A060WGY3_ONCMY 141 158 USERPAT1 . . .
tDEEqAisrVLEaSiaEn
A0A060WGY3_ONCMY 141 158 USERPAT1 . . .
tDEEqAisrVLEaSiaEn

A0A067P993_PLEOS 93 109 USERPAT1 . . .
gEDDeLsr.ALNlSmeTh
A0A067P993_PLEOS 93 109 USERPAT1 . . .
gEDDeLsr.ALNlSmeTh

A0A087QTT4_APTFO 714 730 USERPAT1 . . .
sEDElLaa.VLEiSkrEa
A0A087QTT4_APTFO 714 730 USERPAT1 . . .
sEDElLaa.VLEiSkrEa
A0A087QTT4_APTFO 714 730 USERPAT1 . . .
sEDElLaa.VLEiSkrEa

A0A087Y5I7_POEFO 680 696 USERPAT1 . . .
nDDEmLaa.VLEmSreEa
A0A087Y5I7_POEFO 680 696 USERPAT1 . . .
nDDEmLaa.VLEmSreEa
A0A087Y5I7_POEFO 680 696 USERPAT1 . . .
nDDEmLaa.VLEmSreEa

A0A091CKQ7_FUKDA 281 297 USERPAT1 . . .
sEEDmLqa.AVTmSleTv
A0A091CKQ7_FUKDA 281 297 USERPAT1 . . .
sEEDmLqa.AVTmSleTv

A0A091CKQ7_FUKDA 281 297 USERPAT1 . . .
sEEDmLqa.AVTmSleTv

A0A091CS09_FUKDA 705 721 USERPAT1 . . .
sEEElLaa.VLEiSkrEa
A0A091CS09_FUKDA 705 721 USERPAT1 . . .
sEEElLaa.VLEiSkrEa
A0A091CS09_FUKDA 705 721 USERPAT1 . . .
sEEElLaa.VLEiSkrEa

A0A091EF63_CORBR 714 730 USERPAT1 . . .
sEDElLaa.VLEiSkrEa
A0A091EF63_CORBR 714 730 USERPAT1 . . .
sEDElLaa.VLEiSkrEa
A0A091EF63_CORBR 714 730 USERPAT1 . . .
sEDElLaa.VLEiSkrEa

A0A091FKM6_9AVES 714 730 USERPAT1 . . .
sEDElLaa.VLEiSkrEa
A0A091FKM6_9AVES 714 730 USERPAT1 . . .
sEDElLaa.VLEiSkrEa
A0A091FKM6_9AVES 714 730 USERPAT1 . . .
sEDElLaa.VLEiSkrEa

A0A091IUL0_CALAN 714 730 USERPAT1 . . .
sEDElLaa.VLEiSkrEa
A0A091IUL0_CALAN 714 730 USERPAT1 . . .
sEDElLaa.VLEiSkrEa
A0A091IUL0_CALAN 714 730 USERPAT1 . . .
sEDElLaa.VLEiSkrEa

A0A091JKT2_EGRGA 715 731 USERPAT1 . . .
sEDElLaa.VLEiSkrEa
A0A091JKT2_EGRGA 715 731 USERPAT1 . . .
sEDElLaa.VLEiSkrEa
A0A091JKT2_EGRGA 715 731 USERPAT1 . . .
sEDElLaa.VLEiSkrEa

A0A091UXJ9_NIPNI 715 731 USERPAT1 . . .
sEDElLaa.VLEiSkrEa
A0A091UXJ9_NIPNI 715 731 USERPAT1 . . .
sEDElLaa.VLEiSkrEa
A0A091UXJ9_NIPNI 715 731 USERPAT1 . . .
sEDElLaa.VLEiSkrEa

A0A091VMP1_OPIHO 714 730 USERPAT1 . . .
sEDElLaa.VLEiSkrEa
A0A091VMP1_OPIHO 714 730 USERPAT1 . . .
sEDElLaa.VLEiSkrEa
A0A091VMP1_OPIHO 714 730 USERPAT1 . . .
sEDElLaa.VLEiSkrEa

A0A091VP97_OPIHO 109 125 USERPAT1 . . .
sEEEaLqr.ALEmSlaEs

A0A093FZT0_DRYPU 714 730 USERPAT1 . . .
sEDElLaa.VLEiSkrEa
A0A093FZT0_DRYPU 714 730 USERPAT1 . . .
sEDElLaa.VLEiSkrEa
A0A093FZT0_DRYPU 714 730 USERPAT1 . . .
sEDElLaa.VLEiSkrEa

A0A093J736_STRCA 714 730 USERPAT1 . . .
sEDElLaa.VLEiSkrEa
A0A093J736_STRCA 714 730 USERPAT1 . . .
sEDElLaa.VLEiSkrEa
A0A093J736_STRCA 714 730 USERPAT1 . . .
sEDElLaa.VLEiSkrEa

A0A096MP66_PAPAN 123 140 USERPAT1 . . .
tDEEqAisrVLEaSiaEn
A0A096MP66_PAPAN 123 140 USERPAT1 . . .
tDEEqAisrVLEaSiaEn

A0A096NFH7_PAPAN 705 721 USERPAT1 . . .
sEEElLaa.VLEiSkrDa
A0A096NFH7_PAPAN 705 721 USERPAT1 . . .
sEEElLaa.VLEiSkrDa

A0A096NZ11_PAPAN 123 140 USERPAT1 . . .
tDEEqAisrVLEaSiaEn
A0A096NZ11_PAPAN 123 140 USERPAT1 . . .
tDEEqAisrVLEaSiaEn

A0A099ZA85_TINGU 714 730 USERPAT1 . . .
sEDElLaa.VLEiSkrEa
A0A099ZA85_TINGU 714 730 USERPAT1 . . .
sEDElLaa.VLEiSkrEa
A0A099ZA85_TINGU 714 730 USERPAT1 . . .
sEDElLaa.VLEiSkrEa

A0A099ZXF8_CHAVO 714 730 USERPAT1 . . .
sEDElLaa.VLEiSkrEa
A0A099ZXF8_CHAVO 714 730 USERPAT1 . . .
sEDElLaa.VLEiSkrEa
A0A099ZXF8_CHAVO 714 730 USERPAT1 . . .
sEDElLaa.VLEiSkrEa

A0A0A0MS38_HUMAN 284 300 USERPAT1 . . .
sEEDmLqa.AVTmSleTv
A0A0A0MS38_HUMAN 284 300 USERPAT1 . . .
sEEDmLqa.AVTmSleTv
A0A0A0MS38_HUMAN 284 300 USERPAT1 . . .
sEEDmLqa.AVTmSleTv

A0A0D2N727_9AGAR 164 180 USERPAT1 . . .
kEEEeLqr.VLEmSmqDk

A0A0D9R8Z2_CHLSB 706 722 USERPAT1 . . .
sEEElLaa.VLEiSkrDa
A0A0D9R8Z2_CHLSB 706 722 USERPAT1 . . .
sEEElLaa.VLEiSkrDa

A0A0D9R913_CHLSB 111 128 USERPAT1 . . .
tDEEqAisrVLEaSiaEn
A0A0D9R913_CHLSB 111 128 USERPAT1 . . .
tDEEqAisrVLEaSiaEn

A0A0G4MMG8_9PEZI 816 832 USERPAT1 . . .
dDDEdFkr.ALEaSkiPs
A0A0G4MMG8_9PEZI 816 832 USERPAT1 . . .
dDDEdFkr.ALEaSkiPs
A0A0G4MMG8_9PEZI 816 832 USERPAT1 . . .
dDDEdFkr.ALEaSkiPs
A0A0G4MMG8_9PEZI 816 832 USERPAT1 . . .
dDDEdFkr.ALEaSkiPs
A0A0G4MMG8_9PEZI 816 832 USERPAT1 . . .
dDDEdFkr.ALEaSkiPs

A0A0G4MR85_9PEZI 588 604 USERPAT1 . . .
dDDEdFkr.ALEaSkiPs
A0A0G4MR85_9PEZI 588 604 USERPAT1 . . .
dDDEdFkr.ALEaSkiPs
A0A0G4MR85_9PEZI 588 604 USERPAT1 . . .
dDDEdFkr.ALEaSkiPs
A0A0G4MR85_9PEZI 588 604 USERPAT1 . . .
dDDEdFkr.ALEaSkiPs
A0A0G4MR85_9PEZI 588 604 USERPAT1 . . .
dDDEdFkr.ALEaSkiPs

A0A0H2UKG5_DANRE 670 686 USERPAT1 . . .
nDEEmLaa.VLEmSrhDt
A0A0H2UKG5_DANRE 670 686 USERPAT1 . . .
nDEEmLaa.VLEmSrhDt
A0A0H2UKG5_DANRE 670 686 USERPAT1 . . .
nDEEmLaa.VLEmSrhDt

A0A0K3C6D2_RHOTO 338 354 USERPAT1 . . .
eEDEnLrr.ALElSraGe
A0A0K3C6D2_RHOTO 338 354 USERPAT1 . . .
eEDEnLrr.ALElSraGe
A0A0K3C6D2_RHOTO 338 354 USERPAT1 . . .
eEDEnLrr.ALElSraGe

A0A0L0DRJ6_THETB 140 156 USERPAT1 . . .
dDDEeLqr.VLElSrlEa
A0A0L0DRJ6_THETB 140 156 USERPAT1 . . .
dDDEeLqr.VLElSrlEa

A0A0L0HM51_SPIPD 254 270 USERPAT1 . . .
pEDEdLkr.ALEqSllEg
A0A0L0HM51_SPIPD 254 270 USERPAT1 . . .

pEDEdLkr.ALEqSllEg

A0A0L6WE66_9AGAR 305 322 USERPAT1 . . .
dDEErLlqqALVmSqeGn
A0A0L6WE66_9AGAR 305 322 USERPAT1 . . .
dDEErLlqqALVmSqeGn
A0A0L6WE66_9AGAR 305 322 USERPAT1 . . .
dDEErLlqqALVmSqeGn

A0A0P7U669_SCLFO 677 693 USERPAT1 . . .
nDDEmLaa.VLEmSrqDa
A0A0P7U669_SCLFO 677 693 USERPAT1 . . .
nDDEmLaa.VLEmSrqDa
A0A0P7U669_SCLFO 677 693 USERPAT1 . . .
nDDEmLaa.VLEmSrqDa

A0A0P7UQA9_SCLFO 198 215 USERPAT1 . . .
tDEEqAisrVLEaSiaEn
A0A0P7UQA9_SCLFO 198 215 USERPAT1 . . .
tDEEqAisrVLEaSiaEn

A0A0P7X588_SCLFO 110 127 USERPAT1 . . .
tDEEqAisrVLEaSiaEn
A0A0P7X588_SCLFO 110 127 USERPAT1 . . .
tDEEqAisrVLEaSiaEn

A0A0Q3RFT5_AMAAE 511 527 USERPAT1 . . .
sEDElLaa.VLEiSkrEa
A0A0Q3RFT5_AMAAE 511 527 USERPAT1 . . .
sEDElLaa.VLEiSkrEa
A0A0Q3RFT5_AMAAE 511 527 USERPAT1 . . .
sEDElLaa.VLEiSkrEa

A0A0Q9XLI1_DROMO 568 584 USERPAT1 . . .
eDDEqLqr.VLHeSlmGa
A0A0Q9XLI1_DROMO 568 584 USERPAT1 . . .
eDDEqLqr.VLHeSlmGa
A0A0Q9XLI1_DROMO 568 584 USERPAT1 . . .
eDDEqLqr.VLHeSlmGa
A0A0Q9XLI1_DROMO 568 584 USERPAT1 . . .
eDDEqLqr.VLHeSlmGa

A0A0R4J2D0_MOUSE 704 720 USERPAT1 . . .
sEEEvLaa.VLEiSrrEa
A0A0R4J2D0_MOUSE 704 720 USERPAT1 . . .
sEEEvLaa.VLEiSrrEa
A0A0R4J2D0_MOUSE 704 720 USERPAT1 . . .
sEEEvLaa.VLEiSrrEa

A0A0W8CIL9_PHYNI 330 346 USERPAT1 . . .
dEDEdIrk.ALElSlqTa

A0A151N8X1_ALLMI 710 726 USERPAT1 . . .
sEDElLaa.VLEiSkrEa

A0A151N8X1_ALLMI 710 726 USERPAT1 . . .
sEDElLaa.VLEiSkrEa
A0A151N8X1_ALLMI 710 726 USERPAT1 . . .
sEDElLaa.VLEiSkrEa

A0A168I4K6_MUCCL 304 320 USERPAT1 . . .
nEDDaLqr.ALEmSmnDg
A0A168I4K6_MUCCL 304 320 USERPAT1 . . .
nEDDaLqr.ALEmSmnDg
A0A168I4K6_MUCCL 304 320 USERPAT1 . . .
nEDDaLqr.ALEmSmnDg

A0A178AIS1_9PLEO 1197 1213 USERPAT1 . . .
tDEEdLqk.ALElSmkEh
A0A178AIS1_9PLEO 1197 1213 USERPAT1 . . .
tDEEdLqk.ALElSmkEh

A0A1A6HL89_NEOLE 277 293 USERPAT1 . . .
sEEDiLra.AVTmSleTa
A0A1A6HL89_NEOLE 277 293 USERPAT1 . . .
sEEDiLra.AVTmSleTa
A0A1A6HL89_NEOLE 277 293 USERPAT1 . . .
sEEDiLra.AVTmSleTa

A0A1D2MH25_ORCCI 153 169 USERPAT1 . . .
eEEEnLkk.VLElSlkEq
A0A1D2MH25_ORCCI 153 169 USERPAT1 . . .
eEEEnLkk.VLElSlkEq

A0A1D5Q7L5_MACMU 123 140 USERPAT1 . . .
tDEEqAisrVLEaSiaEn
A0A1D5Q7L5_MACMU 123 140 USERPAT1 . . .
tDEEqAisrVLEaSiaEn

A0A1D5QWL7_MACMU 283 299 USERPAT1 . . .
gEEDmLqa.AVTmSleTv
A0A1D5QWL7_MACMU 283 299 USERPAT1 . . .
gEEDmLqa.AVTmSleTv
A0A1D5QWL7_MACMU 283 299 USERPAT1 . . .
gEEDmLqa.AVTmSleTv

A0A1D5R1Y0_MACMU 278 294 USERPAT1 . . .
gEEDmLqa.AVTmSleTv
A0A1D5R1Y0_MACMU 278 294 USERPAT1 . . .
gEEDmLqa.AVTmSleTv
A0A1D5R1Y0_MACMU 278 294 USERPAT1 . . .
gEEDmLqa.AVTmSleTv

A0A1D5REK4_MACMU 264 280 USERPAT1 . . .
gEEDmLqa.AVTmSleTv
A0A1D5REK4_MACMU 264 280 USERPAT1 . . .
gEEDmLqa.AVTmSleTv
A0A1D5REK4_MACMU 264 280 USERPAT1 . . .
gEEDmLqa.AVTmSleTv

A0A1D5RK12_MACMU 325 341 USERPAT1 . . .
gEEDmLqa.AVTmSleTv
A0A1D5RK12_MACMU 325 341 USERPAT1 . . .
gEEDmLqa.AVTmSleTv
A0A1D5RK12_MACMU 325 341 USERPAT1 . . .
gEEDmLqa.AVTmSleTv

A0A1E4S9B1_CYBJN 256 272 USERPAT1 . . .
dDDDdLkr.ALElSlkEs
A0A1E4S9B1_CYBJN 256 272 USERPAT1 . . .
dDDDdLkr.ALElSlkEs

A0A1I7TNE3_9PELO 485 501 USERPAT1 . . .
dEEEdLrl.ALElSrqEh

A0A1L8ENU6_XENLA 696 712 USERPAT1 . . .
nDEElMaa.VLEmSkrEt
A0A1L8ENU6_XENLA 696 712 USERPAT1 . . .
nDEElMaa.VLEmSkrEt
A0A1L8ENU6_XENLA 696 712 USERPAT1 . . .
nDEElMaa.VLEmSkrEt

A0A1L8EVK7_XENLA 197 213 USERPAT1 . . .
tEEEaLqr.ALEmSlaEs

A0A1L8EVS5_XENLA 702 718 USERPAT1 . . .
nDEElVaa.VLEmSkkEt
A0A1L8EVS5_XENLA 702 718 USERPAT1 . . .
nDEElVaa.VLEmSkkEt

A0A1P8B9V9_ARATH 235 251 USERPAT1 . . .
dEDEqLak.AVEeSlkGk
A0A1P8B9V9_ARATH 235 251 USERPAT1 . . .
dEDEqLak.AVEeSlkGk

A0A1P8B9X5_ARATH 235 251 USERPAT1 . . .
dEDEqLak.AVEeSlkGk
A0A1P8B9X5_ARATH 235 251 USERPAT1 . . .
dEDEqLak.AVEeSlkGk

A0A1S2ZIA5_ERIEU 153 169 USERPAT1 . . .
tEDEaLqr.ALElSleEt

A0A1S2ZIC2_ERIEU 197 213 USERPAT1 . . .
tEDEaLqr.ALElSleEt

A0A1S3ERZ2_DIPOR 680 696 USERPAT1 . . .
sEEElLaa.VLEiSkrEa
A0A1S3ERZ2_DIPOR 680 696 USERPAT1 . . .
sEEElLaa.VLEiSkrEa
A0A1S3ERZ2_DIPOR 680 696 USERPAT1 . . .
sEEElLaa.VLEiSkrEa

A0A1S3ETC3_DIPOR 630 646 USERPAT1 . . .
sEEElLaa.VLEiSkrEa
A0A1S3ETC3_DIPOR 630 646 USERPAT1 . . .
sEEElLaa.VLEiSkrEa
A0A1S3ETC3_DIPOR 630 646 USERPAT1 . . .
sEEElLaa.VLEiSkrEa

A0A1S3EUR3_DIPOR 702 718 USERPAT1 . . .
sEEElLaa.VLEiSkrEa
A0A1S3EUR3_DIPOR 702 718 USERPAT1 . . .
sEEElLaa.VLEiSkrEa
A0A1S3EUR3_DIPOR 702 718 USERPAT1 . . .
sEEElLaa.VLEiSkrEa

A0A1S3F517_DIPOR 224 240 USERPAT1 . . .
eDEEgLqr.ALTlSrqEi
A0A1S3F517_DIPOR 224 240 USERPAT1 . . .
eDEEgLqr.ALTlSrqEi
A0A1S3F517_DIPOR 224 240 USERPAT1 . . .
eDEEgLqr.ALTlSrqEi
A0A1S3F517_DIPOR 329 345 USERPAT1 . . .
sEEDmLqa.AVTmSleTv
A0A1S3F517_DIPOR 329 345 USERPAT1 . . .
sEEDmLqa.AVTmSleTv
A0A1S3F517_DIPOR 329 345 USERPAT1 . . .
sEEDmLqa.AVTmSleTv

A0A1S3F529_DIPOR 223 239 USERPAT1 . . .
eDEEgLqr.ALTlSrqEi
A0A1S3F529_DIPOR 223 239 USERPAT1 . . .
eDEEgLqr.ALTlSrqEi
A0A1S3F529_DIPOR 223 239 USERPAT1 . . .
eDEEgLqr.ALTlSrqEi
A0A1S3F529_DIPOR 328 344 USERPAT1 . . .
sEEDmLqa.AVTmSleTv
A0A1S3F529_DIPOR 328 344 USERPAT1 . . .
sEEDmLqa.AVTmSleTv
A0A1S3F529_DIPOR 328 344 USERPAT1 . . .
sEEDmLqa.AVTmSleTv

A0A1S3F533_DIPOR 217 233 USERPAT1 . . .
eDEEgLqr.ALTlSrqEi
A0A1S3F533_DIPOR 217 233 USERPAT1 . . .
eDEEgLqr.ALTlSrqEi
A0A1S3F533_DIPOR 217 233 USERPAT1 . . .
eDEEgLqr.ALTlSrqEi
A0A1S3F533_DIPOR 322 338 USERPAT1 . . .
sEEDmLqa.AVTmSleTv
A0A1S3F533_DIPOR 322 338 USERPAT1 . . .
sEEDmLqa.AVTmSleTv
A0A1S3F533_DIPOR 322 338 USERPAT1 . . .
sEEDmLqa.AVTmSleTv

A0A1S3F545_DIPOR 173 189 USERPAT1 . . .

eDEEgLqr.ALTlSrqEi
A0A1S3F545_DIPOR 173 189 USERPAT1 . . .
eDEEgLqr.ALTlSrqEi
A0A1S3F545_DIPOR 173 189 USERPAT1 . . .
eDEEgLqr.ALTlSrqEi
A0A1S3F545_DIPOR 278 294 USERPAT1 . . .
sEEDmLqa.AVTmSleTv
A0A1S3F545_DIPOR 278 294 USERPAT1 . . .
sEEDmLqa.AVTmSleTv
A0A1S3F545_DIPOR 278 294 USERPAT1 . . .
sEEDmLqa.AVTmSleTv

A0A1S3F577_DIPOR 177 193 USERPAT1 . . .
eDEEgLqr.ALTlSrqEi
A0A1S3F577_DIPOR 177 193 USERPAT1 . . .
eDEEgLqr.ALTlSrqEi
A0A1S3F577_DIPOR 177 193 USERPAT1 . . .
eDEEgLqr.ALTlSrqEi
A0A1S3F577_DIPOR 282 298 USERPAT1 . . .
sEEDmLqa.AVTmSleTv
A0A1S3F577_DIPOR 282 298 USERPAT1 . . .
sEEDmLqa.AVTmSleTv
A0A1S3F577_DIPOR 282 298 USERPAT1 . . .
sEEDmLqa.AVTmSleTv

A0A1S3F586_DIPOR 224 240 USERPAT1 . . .
eDEEgLqr.ALTlSrqEi
A0A1S3F586_DIPOR 224 240 USERPAT1 . . .
eDEEgLqr.ALTlSrqEi

A0A1S3F638_DIPOR 224 240 USERPAT1 . . .
eDEEgLqr.ALTlSrqEi
A0A1S3F638_DIPOR 224 240 USERPAT1 . . .
eDEEgLqr.ALTlSrqEi
A0A1S3F638_DIPOR 224 240 USERPAT1 . . .
eDEEgLqr.ALTlSrqEi
A0A1S3F638_DIPOR 328 344 USERPAT1 . . .
sEEDmLqa.AVTmSleTv
A0A1S3F638_DIPOR 328 344 USERPAT1 . . .
sEEDmLqa.AVTmSleTv
A0A1S3F638_DIPOR 328 344 USERPAT1 . . .
sEEDmLqa.AVTmSleTv

A0A1S3F6W2_DIPOR 173 189 USERPAT1 . . .
eDEEgLqr.ALTlSrqEi
A0A1S3F6W2_DIPOR 173 189 USERPAT1 . . .
eDEEgLqr.ALTlSrqEi
A0A1S3F6W2_DIPOR 173 189 USERPAT1 . . .
eDEEgLqr.ALTlSrqEi
A0A1S3F6W2_DIPOR 277 293 USERPAT1 . . .
sEEDmLqa.AVTmSleTv
A0A1S3F6W2_DIPOR 277 293 USERPAT1 . . .
sEEDmLqa.AVTmSleTv
A0A1S3F6W2_DIPOR 277 293 USERPAT1 . . .

sEEDmLqa.AVTmSleTv

A0A1S3PLF1_SALSA 663 679 USERPAT1 . . .
nDEEmLaa.VLEmSrqDa
A0A1S3PLF1_SALSA 663 679 USERPAT1 . . .
nDEEmLaa.VLEmSrqDa
A0A1S3PLF1_SALSA 663 679 USERPAT1 . . .
nDEEmLaa.VLEmSrqDa

A0A1S3PME2_SALSA 681 697 USERPAT1 . . .
nDEEmLaa.VLEmSrqDa
A0A1S3PME2_SALSA 681 697 USERPAT1 . . .
nDEEmLaa.VLEmSrqDa
A0A1S3PME2_SALSA 681 697 USERPAT1 . . .
nDEEmLaa.VLEmSrqDa

A0A1S3W742_ERIEU 198 214 USERPAT1 . . .
tEDEaLqr.ALElSleEt

A0A1S3W887_ERIEU 198 214 USERPAT1 . . .
tEDEaLqr.ALElSleEt

A0A1S3WIT1_ERIEU 279 295 USERPAT1 . . .
sEEDmLra.AVTlSleTv
A0A1S3WIT1_ERIEU 279 295 USERPAT1 . . .
sEEDmLra.AVTlSleTv
A0A1S3WIT1_ERIEU 279 295 USERPAT1 . . .
sEEDmLra.AVTlSleTv

A0A1U7Q4D3_MESAU 123 140 USERPAT1 . . .
tDEEqAisrVLEaSiaEn
A0A1U7Q4D3_MESAU 123 140 USERPAT1 . . .
tDEEqAisrVLEaSiaEn

A0A1U7SIU1_ALLSI 721 737 USERPAT1 . . .
sEDElLaa.VLEiSkrEa
A0A1U7SIU1_ALLSI 721 737 USERPAT1 . . .
sEDElLaa.VLEiSkrEa
A0A1U7SIU1_ALLSI 721 737 USERPAT1 . . .
sEDElLaa.VLEiSkrEa

A0A1U7TKV6_TARSY 704 720 USERPAT1 . . .
sEEElLaa.VLEiSkrEa
A0A1U7TKV6_TARSY 704 720 USERPAT1 . . .
sEEElLaa.VLEiSkrEa
A0A1U7TKV6_TARSY 704 720 USERPAT1 . . .
sEEElLaa.VLEiSkrEa

A0A1V2L4I6_CYBFA 259 275 USERPAT1 . . .
dDDEdLkk.ALElSlqEs
A0A1V2L4I6_CYBFA 259 275 USERPAT1 . . .
dDDEdLkk.ALElSlqEs

A0A1V4K6A6_PATFA 714 730 USERPAT1 . . .

sEDElLaa.VLEiSkrEa
A0A1V4K6A6_PATFA 714 730 USERPAT1 . . .
sEDElLaa.VLEiSkrEa
A0A1V4K6A6_PATFA 714 730 USERPAT1 . . .
sEDElLaa.VLEiSkrEa

A0A1X2GN42_9FUNG 291 307 USERPAT1 . . .
dEDEeLqr.ALEmSmnDn
A0A1X2GN42_9FUNG 291 307 USERPAT1 . . .
dEDEeLqr.ALEmSmnDn
A0A1X2GN42_9FUNG 291 307 USERPAT1 . . .
dEDEeLqr.ALEmSmnDn

A0A218VAT4_9PASE 123 140 USERPAT1 . . .
tDEEqAisrVLEaSiaEn
A0A218VAT4_9PASE 123 140 USERPAT1 . . .
tDEEqAisrVLEaSiaEn

A0A218VCA6_9PASE 570 586 USERPAT1 . . .
sEDElLaa.VLEiSkrEa
A0A218VCA6_9PASE 570 586 USERPAT1 . . .
sEDElLaa.VLEiSkrEa
A0A218VCA6_9PASE 570 586 USERPAT1 . . .
sEDElLaa.VLEiSkrEa

A0A226N991_CALSU 121 138 USERPAT1 . . .
tDEEqAisrVLEaSiaEn
A0A226N991_CALSU 121 138 USERPAT1 . . .
tDEEqAisrVLEaSiaEn

A0A226NN36_CALSU 581 597 USERPAT1 . . .
sEDElLaa.VLEiSkrEa
A0A226NN36_CALSU 581 597 USERPAT1 . . .
sEDElLaa.VLEiSkrEa
A0A226NN36_CALSU 581 597 USERPAT1 . . .
sEDElLaa.VLEiSkrEa

A0A226PAP4_COLVI 407 423 USERPAT1 . . .
sEDElLaa.VLEiSkrEa
A0A226PAP4_COLVI 407 423 USERPAT1 . . .
sEDElLaa.VLEiSkrEa
A0A226PAP4_COLVI 407 423 USERPAT1 . . .
sEDElLaa.VLEiSkrEa

A0A286XUX8_CAVPO 684 700 USERPAT1 . . .
nEEElLaa.VLEiSkrEa
A0A286XUX8_CAVPO 684 700 USERPAT1 . . .
nEEElLaa.VLEiSkrEa
A0A286XUX8_CAVPO 684 700 USERPAT1 . . .
nEEElLaa.VLEiSkrEa

A0A286Y2A8_CAVPO 123 140 USERPAT1 . . .
tDEEqAisrVLEaSiaEn
A0A286Y2A8_CAVPO 123 140 USERPAT1 . . .

tDEEqAisrVLEaSiaEn

A0A286ZIY3_PIG 683 699 USERPAT1 . . .
sEEElLaa.VLEiSkrEa
A0A286ZIY3_PIG 683 699 USERPAT1 . . .
sEEElLaa.VLEiSkrEa
A0A286ZIY3_PIG 683 699 USERPAT1 . . .
sEEElLaa.VLEiSkrEa

A0A287AIY3_PIG 595 611 USERPAT1 . . .
sEEElLaa.VLEiSkrEa
A0A287AIY3_PIG 595 611 USERPAT1 . . .
sEEElLaa.VLEiSkrEa
A0A287AIY3_PIG 595 611 USERPAT1 . . .
sEEElLaa.VLEiSkrEa

A0A2B4RED8_STYPI 12 29 USERPAT1 . . .
eDEDeAlakALElSslEq

A0A2C9F3C4_PIG 705 721 USERPAT1 . . .
sEEElLaa.VLEiSkrEa
A0A2C9F3C4_PIG 705 721 USERPAT1 . . .
sEEElLaa.VLEiSkrEa
A0A2C9F3C4_PIG 705 721 USERPAT1 . . .
sEEElLaa.VLEiSkrEa

A0A2C9JDB1_BIOGL 563 579 USERPAT1 . . .
rEEEdFqm.VLElSrrEq
A0A2C9JDB1_BIOGL 563 579 USERPAT1 . . .
rEEEdFqm.VLElSrrEq
A0A2C9JDB1_BIOGL 563 579 USERPAT1 . . .
rEEEdFqm.VLElSrrEq

A0A2C9JKV6_BIOGL 270 286 USERPAT1 . . .
vDEEmLer.ALGaSlnPd
A0A2C9JKV6_BIOGL 270 286 USERPAT1 . . .
vDEEmLer.ALGaSlnPd
A0A2C9JKV6_BIOGL 270 286 USERPAT1 . . .
vDEEmLer.ALGaSlnPd

A0A2D0R3W6_ICTPU 674 690 USERPAT1 . . .
nDDEmLaa.VLEmSrqDa
A0A2D0R3W6_ICTPU 674 690 USERPAT1 . . .
nDDEmLaa.VLEmSrqDa
A0A2D0R3W6_ICTPU 674 690 USERPAT1 . . .
nDDEmLaa.VLEmSrqDa

A0A2I0LKF1_COLLI 714 730 USERPAT1 . . .
sEDElLaa.VLEiSkrEa
A0A2I0LKF1_COLLI 714 730 USERPAT1 . . .
sEDElLaa.VLEiSkrEa
A0A2I0LKF1_COLLI 714 730 USERPAT1 . . .
sEDElLaa.VLEiSkrEa

A0A2I0MS83_COLLI 194 211 USERPAT1 . . .
tDEEqAisrVLEaSiaEn
A0A2I0MS83_COLLI 194 211 USERPAT1 . . .
tDEEqAisrVLEaSiaEn

A0A2I2UUV1_FELCA 123 140 USERPAT1 . . .
tDEEqAisrVLEaSiaEn
A0A2I2UUV1_FELCA 123 140 USERPAT1 . . .
tDEEqAisrVLEaSiaEn

A0A2I2Y2A1_GORGO 123 140 USERPAT1 . . .
tDEEqAisrVLEaSiaEn
A0A2I2Y2A1_GORGO 123 140 USERPAT1 . . .
tDEEqAisrVLEaSiaEn

A0A2I2Y7T6_GORGO 316 332 USERPAT1 . . .
sEEDmLqa.AVTmSleTv
A0A2I2Y7T6_GORGO 316 332 USERPAT1 . . .
sEEDmLqa.AVTmSleTv
A0A2I2Y7T6_GORGO 316 332 USERPAT1 . . .
sEEDmLqa.AVTmSleTv

A0A2I2YBD4_GORGO 116 133 USERPAT1 . . .
tDEEqAisrVLEaSiaEn
A0A2I2YBD4_GORGO 116 133 USERPAT1 . . .
tDEEqAisrVLEaSiaEn

A0A2I2YJG1_GORGO 331 347 USERPAT1 . . .
sEEDmLqa.AVTmSleTv
A0A2I2YJG1_GORGO 331 347 USERPAT1 . . .
sEEDmLqa.AVTmSleTv
A0A2I2YJG1_GORGO 331 347 USERPAT1 . . .
sEEDmLqa.AVTmSleTv

A0A2I2YSI6_GORGO 340 356 USERPAT1 . . .
sEEDmLqa.AVTmSleTv
A0A2I2YSI6_GORGO 340 356 USERPAT1 . . .
sEEDmLqa.AVTmSleTv
A0A2I2YSI6_GORGO 340 356 USERPAT1 . . .
sEEDmLqa.AVTmSleTv

A0A2I2YVJ0_GORGO 123 140 USERPAT1 . . .
tDEEqAisrVLEaSiaEn
A0A2I2YVJ0_GORGO 123 140 USERPAT1 . . .
tDEEqAisrVLEaSiaEn

A0A2I2Z4Q3_GORGO 123 140 USERPAT1 . . .
tDEEqAisrVLEaSiaEn
A0A2I2Z4Q3_GORGO 123 140 USERPAT1 . . .
tDEEqAisrVLEaSiaEn

A0A2I2ZD42_GORGO 116 133 USERPAT1 . . .
tDEEqAisrVLEaSiaEn
A0A2I2ZD42_GORGO 116 133 USERPAT1 . . .

tDEEqAisrVLEaSiaEn

A0A2I2ZJ77_GORGO 280 296 USERPAT1 . . .
sEEDmLqa.AVTmSleTv
A0A2I2ZJ77_GORGO 280 296 USERPAT1 . . .
sEEDmLqa.AVTmSleTv
A0A2I2ZJ77_GORGO 280 296 USERPAT1 . . .
sEEDmLqa.AVTmSleTv

A0A2I2ZV90_GORGO 704 720 USERPAT1 . . .
sEEElLaa.VLEiSkrDa
A0A2I2ZV90_GORGO 704 720 USERPAT1 . . .
sEEElLaa.VLEiSkrDa

A0A2I3FVD2_NOMLE 123 140 USERPAT1 . . .
tDEEqAisrVLEaSiaEn
A0A2I3FVD2_NOMLE 123 140 USERPAT1 . . .
tDEEqAisrVLEaSiaEn

A0A2I3G3E4_NOMLE 265 281 USERPAT1 . . .
sEEDmLqa.AVTmSleTv
A0A2I3G3E4_NOMLE 265 281 USERPAT1 . . .
sEEDmLqa.AVTmSleTv
A0A2I3G3E4_NOMLE 265 281 USERPAT1 . . .
sEEDmLqa.AVTmSleTv

A0A2I3GEZ4_NOMLE 320 336 USERPAT1 . . .
sEEDmLqa.AVTmSleTv
A0A2I3GEZ4_NOMLE 320 336 USERPAT1 . . .
sEEDmLqa.AVTmSleTv
A0A2I3GEZ4_NOMLE 320 336 USERPAT1 . . .
sEEDmLqa.AVTmSleTv

A0A2I3GGH3_NOMLE 123 140 USERPAT1 . . .
tDEEqAisrVLEaSiaEn
A0A2I3GGH3_NOMLE 123 140 USERPAT1 . . .
tDEEqAisrVLEaSiaEn

A0A2I3GHK3_NOMLE 305 321 USERPAT1 . . .
sEEDmLqa.AVTmSleTv
A0A2I3GHK3_NOMLE 305 321 USERPAT1 . . .
sEEDmLqa.AVTmSleTv
A0A2I3GHK3_NOMLE 305 321 USERPAT1 . . .
sEEDmLqa.AVTmSleTv

A0A2I3H427_NOMLE 268 284 USERPAT1 . . .
sEEDmLqa.AVTmSleTv
A0A2I3H427_NOMLE 268 284 USERPAT1 . . .
sEEDmLqa.AVTmSleTv
A0A2I3H427_NOMLE 268 284 USERPAT1 . . .
sEEDmLqa.AVTmSleTv

A0A2I3HII5_NOMLE 123 140 USERPAT1 . . .
tDEEqAisrVLEaSiaEn

A0A2I3HII5_NOMLE 123 140 USERPAT1 . . .
tDEEqAisrVLEaSiaEn

A0A2I3HL68_NOMLE 706 722 USERPAT1 . . .
sEEElLaa.VLEiSkrDa
A0A2I3HL68_NOMLE 706 722 USERPAT1 . . .
sEEElLaa.VLEiSkrDa

A0A2I3HS80_NOMLE 329 345 USERPAT1 . . .
sEEDmLqa.AVTmSleTv
A0A2I3HS80_NOMLE 329 345 USERPAT1 . . .
sEEDmLqa.AVTmSleTv
A0A2I3HS80_NOMLE 329 345 USERPAT1 . . .
sEEDmLqa.AVTmSleTv

A0A2I3HTW1_NOMLE 612 628 USERPAT1 . . .
sEEElLaa.VLEiSkrDa
A0A2I3HTW1_NOMLE 612 628 USERPAT1 . . .
sEEElLaa.VLEiSkrDa

A0A2I3LC42_PAPAN 611 627 USERPAT1 . . .
sEEElLaa.VLEiSkrDa
A0A2I3LC42_PAPAN 611 627 USERPAT1 . . .
sEEElLaa.VLEiSkrDa

A0A2I3LQ44_PAPAN 123 140 USERPAT1 . . .
tDEEqAisrVLEaSiaEn
A0A2I3LQ44_PAPAN 123 140 USERPAT1 . . .
tDEEqAisrVLEaSiaEn

A0A2I3MAN5_PAPAN 340 356 USERPAT1 . . .
gEEDmLqa.AVTmSleTv
A0A2I3MAN5_PAPAN 340 356 USERPAT1 . . .
gEEDmLqa.AVTmSleTv
A0A2I3MAN5_PAPAN 340 356 USERPAT1 . . .
gEEDmLqa.AVTmSleTv

A0A2I3MIB0_PAPAN 116 133 USERPAT1 . . .
tDEEqAisrVLEaSiaEn
A0A2I3MIB0_PAPAN 116 133 USERPAT1 . . .
tDEEqAisrVLEaSiaEn

A0A2I3MVB2_PAPAN 326 342 USERPAT1 . . .
gEEDmLqa.AVTmSleTv
A0A2I3MVB2_PAPAN 326 342 USERPAT1 . . .
gEEDmLqa.AVTmSleTv
A0A2I3MVB2_PAPAN 326 342 USERPAT1 . . .
gEEDmLqa.AVTmSleTv

A0A2I3MY00_PAPAN 123 140 USERPAT1 . . .
tDEEqAisrVLEaSiaEn
A0A2I3MY00_PAPAN 123 140 USERPAT1 . . .
tDEEqAisrVLEaSiaEn

A0A2I3RD20_PANTR 324 340 USERPAT1 . . .
sEEDmLqa.AVTmSleTv
A0A2I3RD20_PANTR 324 340 USERPAT1 . . .
sEEDmLqa.AVTmSleTv
A0A2I3RD20_PANTR 324 340 USERPAT1 . . .
sEEDmLqa.AVTmSleTv

A0A2I3RF30_PANTR 123 140 USERPAT1 . . .
tDEEqAisrVLEaSiaEn
A0A2I3RF30_PANTR 123 140 USERPAT1 . . .
tDEEqAisrVLEaSiaEn

A0A2I3RW24_PANTR 123 140 USERPAT1 . . .
tDEEqAisrVLEaSiaEn
A0A2I3RW24_PANTR 123 140 USERPAT1 . . .
tDEEqAisrVLEaSiaEn

A0A2I3S0Z0_PANTR 323 339 USERPAT1 . . .
sEEDmLqa.AVTmSleTv
A0A2I3S0Z0_PANTR 323 339 USERPAT1 . . .
sEEDmLqa.AVTmSleTv
A0A2I3S0Z0_PANTR 323 339 USERPAT1 . . .
sEEDmLqa.AVTmSleTv

A0A2I3S1H4_PANTR 610 626 USERPAT1 . . .
sEEElLaa.VLEiSkrDa
A0A2I3S1H4_PANTR 610 626 USERPAT1 . . .
sEEElLaa.VLEiSkrDa

A0A2I3SC06_PANTR 123 140 USERPAT1 . . .
tDEEqAisrVLEaSiaEn
A0A2I3SC06_PANTR 123 140 USERPAT1 . . .
tDEEqAisrVLEaSiaEn

A0A2I3TCN4_PANTR 288 304 USERPAT1 . . .
sEEDmLqa.AVTmSleTv
A0A2I3TCN4_PANTR 288 304 USERPAT1 . . .
sEEDmLqa.AVTmSleTv
A0A2I3TCN4_PANTR 288 304 USERPAT1 . . .
sEEDmLqa.AVTmSleTv

A0A2I3TPK5_PANTR 123 140 USERPAT1 . . .
tDEEqAisrVLEaSiaEn
A0A2I3TPK5_PANTR 123 140 USERPAT1 . . .
tDEEqAisrVLEaSiaEn

A0A2I3TRW8_PANTR 269 285 USERPAT1 . . .
sEEDmLqa.AVTmSleTv
A0A2I3TRW8_PANTR 269 285 USERPAT1 . . .
sEEDmLqa.AVTmSleTv
A0A2I3TRW8_PANTR 269 285 USERPAT1 . . .
sEEDmLqa.AVTmSleTv

A0A2I3TWP0_PANTR 704 720 USERPAT1 . . .

```
sEEElLaa.VLEiSkrDa
A0A2I3TWP0_PANTR 704      720      USERPAT1 .        .        .
sEEElLaa.VLEiSkrDa

A0A2I3TX18_PANTR 284      300      USERPAT1 .        .        .
sEEDmLqa.AVTmSleTv
A0A2I3TX18_PANTR 284      300      USERPAT1 .        .        .
sEEDmLqa.AVTmSleTv
A0A2I3TX18_PANTR 284      300      USERPAT1 .        .        .
sEEDmLqa.AVTmSleTv

A0A2I4BZG2_9TELE 681      697      USERPAT1 .        .        .
nDDEmMaa.VLEmSrqEa
A0A2I4BZG2_9TELE 681      697      USERPAT1 .        .        .
nDDEmMaa.VLEmSrqEa
A0A2I4BZG2_9TELE 681      697      USERPAT1 .        .        .
nDDEmMaa.VLEmSrqEa

A0A2I4BZG3_9TELE 680      696      USERPAT1 .        .        .
nDDEmMaa.VLEmSrqEa
A0A2I4BZG3_9TELE 680      696      USERPAT1 .        .        .
nDDEmMaa.VLEmSrqEa
A0A2I4BZG3_9TELE 680      696      USERPAT1 .        .        .
nDDEmMaa.VLEmSrqEa

A0A2J8EJY0_VERDA 1167     1183     USERPAT1 .        .        .
dDDEdFkr.ALEaSkiPs
A0A2J8EJY0_VERDA 1167     1183     USERPAT1 .        .        .
dDDEdFkr.ALEaSkiPs
A0A2J8EJY0_VERDA 1167     1183     USERPAT1 .        .        .
dDDEdFkr.ALEaSkiPs
A0A2J8EJY0_VERDA 1167     1183     USERPAT1 .        .        .
dDDEdFkr.ALEaSkiPs

A0A2K1XRX5_POPTR 122      138      USERPAT1 .        .        .
vEDDeFar.ALElSlkTa
A0A2K1XRX5_POPTR 122      138      USERPAT1 .        .        .
vEDDeFar.ALElSlkTa

A0A2K1XRY2_POPTR 190      206      USERPAT1 .        .        .
vEDDeFar.ALElSlkTa
A0A2K1XRY2_POPTR 190      206      USERPAT1 .        .        .
vEDDeFar.ALElSlkTa

A0A2K1XRY3_POPTR 190      206      USERPAT1 .        .        .
vEDDeFar.ALElSlkTa
A0A2K1XRY3_POPTR 190      206      USERPAT1 .        .        .
vEDDeFar.ALElSlkTa

A0A2K2DCV4_BRADI 66       82       USERPAT1 .        .        .
dDDDnLqa.ALEaSkeEt
A0A2K2DCV4_BRADI 66       82       USERPAT1 .        .        .
dDDDnLqa.ALEaSkeEt
A0A2K2DCV4_BRADI 66       82       USERPAT1 .        .        .
```

dDDDnLqa.ALEaSkeEt
A0A2K2DCV4_BRADI 116 132 USERPAT1 . . .
eEEDdLqa.ALEtSkeEi
A0A2K2DCV4_BRADI 116 132 USERPAT1 . . .
eEEDdLqa.ALEtSkeEi
A0A2K2DCV4_BRADI 116 132 USERPAT1 . . .
eEEDdLqa.ALEtSkeEi

A0A2K5DCH2_AOTNA 123 140 USERPAT1 . . .
tDEEqAisrVLEaSiaEn
A0A2K5DCH2_AOTNA 123 140 USERPAT1 . . .
tDEEqAisrVLEaSiaEn

A0A2K5DCJ1_AOTNA 123 140 USERPAT1 . . .
tDEEqAisrVLEaSiaEn
A0A2K5DCJ1_AOTNA 123 140 USERPAT1 . . .
tDEEqAisrVLEaSiaEn

A0A2K5DCJ3_AOTNA 123 140 USERPAT1 . . .
tDEEqAisrVLEaSiaEn
A0A2K5DCJ3_AOTNA 123 140 USERPAT1 . . .
tDEEqAisrVLEaSiaEn

A0A2K5DCK2_AOTNA 123 140 USERPAT1 . . .
tDEEqAisrVLEaSiaEn
A0A2K5DCK2_AOTNA 123 140 USERPAT1 . . .
tDEEqAisrVLEaSiaEn

A0A2K5DCK7_AOTNA 119 136 USERPAT1 . . .
tDEEqAisrVLEaSiaEn
A0A2K5DCK7_AOTNA 119 136 USERPAT1 . . .
tDEEqAisrVLEaSiaEn

A0A2K5E0F1_AOTNA 300 316 USERPAT1 . . .
sEEDmLqa.AVTmSleTv
A0A2K5E0F1_AOTNA 300 316 USERPAT1 . . .
sEEDmLqa.AVTmSleTv
A0A2K5E0F1_AOTNA 300 316 USERPAT1 . . .
sEEDmLqa.AVTmSleTv

A0A2K5E0J7_AOTNA 279 295 USERPAT1 . . .
sEEDmLqa.AVTmSleTv
A0A2K5E0J7_AOTNA 279 295 USERPAT1 . . .
sEEDmLqa.AVTmSleTv
A0A2K5E0J7_AOTNA 279 295 USERPAT1 . . .
sEEDmLqa.AVTmSleTv

A0A2K5E0K4_AOTNA 239 255 USERPAT1 . . .
sEEDmLqa.AVTmSleTv
A0A2K5E0K4_AOTNA 239 255 USERPAT1 . . .
sEEDmLqa.AVTmSleTv
A0A2K5E0K4_AOTNA 239 255 USERPAT1 . . .
sEEDmLqa.AVTmSleTv

A0A2K5E0M2_AOTNA 243 259 USERPAT1 . . .
sEEDmLqa.AVTmSleTv
A0A2K5E0M2_AOTNA 243 259 USERPAT1 . . .
sEEDmLqa.AVTmSleTv
A0A2K5E0M2_AOTNA 243 259 USERPAT1 . . .
sEEDmLqa.AVTmSleTv

A0A2K5E0Q9_AOTNA 224 240 USERPAT1 . . .
sEEDmLqa.AVTmSleTv
A0A2K5E0Q9_AOTNA 224 240 USERPAT1 . . .
sEEDmLqa.AVTmSleTv
A0A2K5E0Q9_AOTNA 224 240 USERPAT1 . . .
sEEDmLqa.AVTmSleTv

A0A2K5E0S1_AOTNA 294 310 USERPAT1 . . .
sEEDmLqa.AVTmSleTv
A0A2K5E0S1_AOTNA 294 310 USERPAT1 . . .
sEEDmLqa.AVTmSleTv
A0A2K5E0S1_AOTNA 294 310 USERPAT1 . . .
sEEDmLqa.AVTmSleTv

A0A2K5E4H2_AOTNA 705 721 USERPAT1 . . .
sEEEvLaa.VLEiSkrEa
A0A2K5E4H2_AOTNA 705 721 USERPAT1 . . .
sEEEvLaa.VLEiSkrEa
A0A2K5E4H2_AOTNA 705 721 USERPAT1 . . .
sEEEvLaa.VLEiSkrEa

A0A2K5E4T1_AOTNA 611 627 USERPAT1 . . .
sEEEvLaa.VLEiSkrEa
A0A2K5E4T1_AOTNA 611 627 USERPAT1 . . .
sEEEvLaa.VLEiSkrEa
A0A2K5E4T1_AOTNA 611 627 USERPAT1 . . .
sEEEvLaa.VLEiSkrEa

A0A2K5HP42_COLAP 278 294 USERPAT1 . . .
gEEDmLqa.AVTmSleTv
A0A2K5HP42_COLAP 278 294 USERPAT1 . . .
gEEDmLqa.AVTmSleTv
A0A2K5HP42_COLAP 278 294 USERPAT1 . . .
gEEDmLqa.AVTmSleTv

A0A2K5HP48_COLAP 293 309 USERPAT1 . . .
gEEDmLqa.AVTmSleTv
A0A2K5HP48_COLAP 293 309 USERPAT1 . . .
gEEDmLqa.AVTmSleTv
A0A2K5HP48_COLAP 293 309 USERPAT1 . . .
gEEDmLqa.AVTmSleTv

A0A2K5HP53_COLAP 242 258 USERPAT1 . . .
gEEDmLqa.AVTmSleTv
A0A2K5HP53_COLAP 242 258 USERPAT1 . . .
gEEDmLqa.AVTmSleTv
A0A2K5HP53_COLAP 242 258 USERPAT1 . . .

gEEDmLqa.AVTmSleTv

A0A2K5HP85_COLAP 285 301 USERPAT1 . . .
gEEDmLqa.AVTmSleTv
A0A2K5HP85_COLAP 285 301 USERPAT1 . . .
gEEDmLqa.AVTmSleTv
A0A2K5HP85_COLAP 285 301 USERPAT1 . . .
gEEDmLqa.AVTmSleTv

A0A2K5HPV2_COLAP 706 722 USERPAT1 . . .
sEEElLaa.VLEiSkrDa
A0A2K5HPV2_COLAP 706 722 USERPAT1 . . .
sEEElLaa.VLEiSkrDa

A0A2K5HPW3_COLAP 612 628 USERPAT1 . . .
sEEElLaa.VLEiSkrDa
A0A2K5HPW3_COLAP 612 628 USERPAT1 . . .
sEEElLaa.VLEiSkrDa

A0A2K5J3H9_COLAP 120 137 USERPAT1 . . .
tDEEqAisrVLEaSiaEn
A0A2K5J3H9_COLAP 120 137 USERPAT1 . . .
tDEEqAisrVLEaSiaEn

A0A2K5J3K2_COLAP 120 137 USERPAT1 . . .
tDEEqAisrVLEaSiaEn
A0A2K5J3K2_COLAP 120 137 USERPAT1 . . .
tDEEqAisrVLEaSiaEn

A0A2K5J3K7_COLAP 108 125 USERPAT1 . . .
tDEEqAisrVLEaSiaEn
A0A2K5J3K7_COLAP 108 125 USERPAT1 . . .
tDEEqAisrVLEaSiaEn

A0A2K5J3L2_COLAP 108 125 USERPAT1 . . .
tDEEqAisrVLEaSiaEn
A0A2K5J3L2_COLAP 108 125 USERPAT1 . . .
tDEEqAisrVLEaSiaEn

A0A2K5KGJ4_COLAP 329 345 USERPAT1 . . .
gEEDmLqa.AVTtSleTv
A0A2K5KGJ4_COLAP 329 345 USERPAT1 . . .
gEEDmLqa.AVTtSleTv
A0A2K5KGJ4_COLAP 329 345 USERPAT1 . . .
gEEDmLqa.AVTtSleTv

A0A2K5L5F2_CERAT 612 628 USERPAT1 . . .
sEEElLaa.VLEiSkrDa
A0A2K5L5F2_CERAT 612 628 USERPAT1 . . .
sEEElLaa.VLEiSkrDa

A0A2K5L5F4_CERAT 706 722 USERPAT1 . . .
sEEElLaa.VLEiSkrDa
A0A2K5L5F4_CERAT 706 722 USERPAT1 . . .

sEEElLaa.VLEiSkrDa

A0A2K5MRI6_CERAT 339 355 USERPAT1 . . .
gEEDmLqa.AVTmSleTv
A0A2K5MRI6_CERAT 339 355 USERPAT1 . . .
gEEDmLqa.AVTmSleTv
A0A2K5MRI6_CERAT 339 355 USERPAT1 . . .
gEEDmLqa.AVTmSleTv

A0A2K5MRJ5_CERAT 286 302 USERPAT1 . . .
gEEDmLqa.AVTmSleTv
A0A2K5MRJ5_CERAT 286 302 USERPAT1 . . .
gEEDmLqa.AVTmSleTv
A0A2K5MRJ5_CERAT 286 302 USERPAT1 . . .
gEEDmLqa.AVTmSleTv

A0A2K5MRK2_CERAT 282 298 USERPAT1 . . .
gEEDmLqa.AVTmSleTv
A0A2K5MRK2_CERAT 282 298 USERPAT1 . . .
gEEDmLqa.AVTmSleTv
A0A2K5MRK2_CERAT 282 298 USERPAT1 . . .
gEEDmLqa.AVTmSleTv

A0A2K5MRL3_CERAT 337 353 USERPAT1 . . .
gEEDmLqa.AVTmSleTv
A0A2K5MRL3_CERAT 337 353 USERPAT1 . . .
gEEDmLqa.AVTmSleTv
A0A2K5MRL3_CERAT 337 353 USERPAT1 . . .
gEEDmLqa.AVTmSleTv

A0A2K5MRX7_CERAT 322 338 USERPAT1 . . .
gEEDmLqa.AVTmSleTv
A0A2K5MRX7_CERAT 322 338 USERPAT1 . . .
gEEDmLqa.AVTmSleTv
A0A2K5MRX7_CERAT 322 338 USERPAT1 . . .
gEEDmLqa.AVTmSleTv

A0A2K5P5B4_CERAT 123 140 USERPAT1 . . .
tDEEqAisrVLEaSiaEn
A0A2K5P5B4_CERAT 123 140 USERPAT1 . . .
tDEEqAisrVLEaSiaEn

A0A2K5P5B6_CERAT 123 140 USERPAT1 . . .
tDEEqAisrVLEaSiaEn
A0A2K5P5B6_CERAT 123 140 USERPAT1 . . .
tDEEqAisrVLEaSiaEn

A0A2K5P5C1_CERAT 123 140 USERPAT1 . . .
tDEEqAisrVLEaSiaEn
A0A2K5P5C1_CERAT 123 140 USERPAT1 . . .
tDEEqAisrVLEaSiaEn

A0A2K5P5C3_CERAT 123 140 USERPAT1 . . .
tDEEqAisrVLEaSiaEn

A0A2K5P5C3_CERAT 123 140 USERPAT1 . . .
tDEEqAisrVLEaSiaEn

A0A2K5P5D7_CERAT 116 133 USERPAT1 . . .
tDEEqAisrVLEaSiaEn
A0A2K5P5D7_CERAT 116 133 USERPAT1 . . .
tDEEqAisrVLEaSiaEn

A0A2K5QRZ1_CEBCA 260 276 USERPAT1 . . .
sEEDmLqa.AVTmSleTv
A0A2K5QRZ1_CEBCA 260 276 USERPAT1 . . .
sEEDmLqa.AVTmSleTv
A0A2K5QRZ1_CEBCA 260 276 USERPAT1 . . .
sEEDmLqa.AVTmSleTv

A0A2K5QS50_CEBCA 279 295 USERPAT1 . . .
sEEDmLqa.AVTmSleTv
A0A2K5QS50_CEBCA 279 295 USERPAT1 . . .
sEEDmLqa.AVTmSleTv
A0A2K5QS50_CEBCA 279 295 USERPAT1 . . .
sEEDmLqa.AVTmSleTv

A0A2K5QS64_CEBCA 243 259 USERPAT1 . . .
sEEDmLqa.AVTmSleTv
A0A2K5QS64_CEBCA 243 259 USERPAT1 . . .
sEEDmLqa.AVTmSleTv
A0A2K5QS64_CEBCA 243 259 USERPAT1 . . .
sEEDmLqa.AVTmSleTv

A0A2K5QS73_CEBCA 239 255 USERPAT1 . . .
sEEDmLqa.AVTmSleTv
A0A2K5QS73_CEBCA 239 255 USERPAT1 . . .
sEEDmLqa.AVTmSleTv
A0A2K5QS73_CEBCA 239 255 USERPAT1 . . .
sEEDmLqa.AVTmSleTv

A0A2K5QS87_CEBCA 296 312 USERPAT1 . . .
sEEDmLqa.AVTmSleTv
A0A2K5QS87_CEBCA 296 312 USERPAT1 . . .
sEEDmLqa.AVTmSleTv
A0A2K5QS87_CEBCA 296 312 USERPAT1 . . .
sEEDmLqa.AVTmSleTv

A0A2K5QS88_CEBCA 224 240 USERPAT1 . . .
sEEDmLqa.AVTmSleTv
A0A2K5QS88_CEBCA 224 240 USERPAT1 . . .
sEEDmLqa.AVTmSleTv
A0A2K5QS88_CEBCA 224 240 USERPAT1 . . .
sEEDmLqa.AVTmSleTv

A0A2K5S783_CEBCA 123 140 USERPAT1 . . .
tDEEqAisrVLEaSiaEn
A0A2K5S783_CEBCA 123 140 USERPAT1 . . .
tDEEqAisrVLEaSiaEn

A0A2K5S795_CEBCA 123 140 USERPAT1 . . .
tDEEqAisrVLEaSiaEn
A0A2K5S795_CEBCA 123 140 USERPAT1 . . .
tDEEqAisrVLEaSiaEn

A0A2K5S798_CEBCA 123 140 USERPAT1 . . .
tDEEqAisrVLEaSiaEn
A0A2K5S798_CEBCA 123 140 USERPAT1 . . .
tDEEqAisrVLEaSiaEn

A0A2K5S7A2_CEBCA 123 140 USERPAT1 . . .
tDEEqAisrVLEaSiaEn
A0A2K5S7A2_CEBCA 123 140 USERPAT1 . . .
tDEEqAisrVLEaSiaEn

A0A2K5S7A3_CEBCA 119 136 USERPAT1 . . .
tDEEqAisrVLEaSiaEn
A0A2K5S7A3_CEBCA 119 136 USERPAT1 . . .
tDEEqAisrVLEaSiaEn

A0A2K5SAG7_CEBCA 612 628 USERPAT1 . . .
sEEElLaa.VLEiSkrEa
A0A2K5SAG7_CEBCA 612 628 USERPAT1 . . .
sEEElLaa.VLEiSkrEa
A0A2K5SAG7_CEBCA 612 628 USERPAT1 . . .
sEEElLaa.VLEiSkrEa

A0A2K5SAI6_CEBCA 706 722 USERPAT1 . . .
sEEElLaa.VLEiSkrEa
A0A2K5SAI6_CEBCA 706 722 USERPAT1 . . .
sEEElLaa.VLEiSkrEa
A0A2K5SAI6_CEBCA 706 722 USERPAT1 . . .
sEEElLaa.VLEiSkrEa

A0A2K5VMK7_MACFA 319 335 USERPAT1 . . .
gEEDmLqa.AVTmSleTv
A0A2K5VMK7_MACFA 319 335 USERPAT1 . . .
gEEDmLqa.AVTmSleTv
A0A2K5VMK7_MACFA 319 335 USERPAT1 . . .
gEEDmLqa.AVTmSleTv

A0A2K5VMM1_MACFA 334 350 USERPAT1 . . .
gEEDmLqa.AVTmSleTv
A0A2K5VMM1_MACFA 334 350 USERPAT1 . . .
gEEDmLqa.AVTmSleTv
A0A2K5VMM1_MACFA 334 350 USERPAT1 . . .
gEEDmLqa.AVTmSleTv

A0A2K5VMQ2_MACFA 279 295 USERPAT1 . . .
gEEDmLqa.AVTmSleTv
A0A2K5VMQ2_MACFA 279 295 USERPAT1 . . .
gEEDmLqa.AVTmSleTv
A0A2K5VMQ2_MACFA 279 295 USERPAT1 . . .

gEEDmLqa.AVTmSleTv

A0A2K5VMU2_MACFA 340 356 USERPAT1 . . .
gEEDmLqa.AVTmSleTv
A0A2K5VMU2_MACFA 340 356 USERPAT1 . . .
gEEDmLqa.AVTmSleTv
A0A2K5VMU2_MACFA 340 356 USERPAT1 . . .
gEEDmLqa.AVTmSleTv

A0A2K5VMX8_MACFA 340 356 USERPAT1 . . .
gEEDmLqa.AVTmSleTv
A0A2K5VMX8_MACFA 340 356 USERPAT1 . . .
gEEDmLqa.AVTmSleTv
A0A2K5VMX8_MACFA 340 356 USERPAT1 . . .
gEEDmLqa.AVTmSleTv

A0A2K5WBW8_MACFA 706 722 USERPAT1 . . .
sEEElLaa.VLEiSkrDa
A0A2K5WBW8_MACFA 706 722 USERPAT1 . . .
sEEElLaa.VLEiSkrDa

A0A2K5WBY8_MACFA 612 628 USERPAT1 . . .
sEEElLaa.VLEiSkrDa
A0A2K5WBY8_MACFA 612 628 USERPAT1 . . .
sEEElLaa.VLEiSkrDa

A0A2K5X505_MACFA 123 140 USERPAT1 . . .
tDEEqAisrVLEaSiaEn
A0A2K5X505_MACFA 123 140 USERPAT1 . . .
tDEEqAisrVLEaSiaEn

A0A2K5X566_MACFA 123 140 USERPAT1 . . .
tDEEqAisrVLEaSiaEn
A0A2K5X566_MACFA 123 140 USERPAT1 . . .
tDEEqAisrVLEaSiaEn

A0A2K5X5E1_MACFA 123 140 USERPAT1 . . .
tDEEqAisrVLEaSiaEn
A0A2K5X5E1_MACFA 123 140 USERPAT1 . . .
tDEEqAisrVLEaSiaEn

A0A2K5X5F0_MACFA 123 140 USERPAT1 . . .
tDEEqAisrVLEaSiaEn
A0A2K5X5F0_MACFA 123 140 USERPAT1 . . .
tDEEqAisrVLEaSiaEn

A0A2K5X5I1_MACFA 116 133 USERPAT1 . . .
tDEEqAisrVLEaSiaEn
A0A2K5X5I1_MACFA 116 133 USERPAT1 . . .
tDEEqAisrVLEaSiaEn

A0A2K5XCF0_MANLE 116 133 USERPAT1 . . .
tDEEqAisrVLEaSiaEn
A0A2K5XCF0_MANLE 116 133 USERPAT1 . . .

tDEEqAisrVLEaSiaEn

A0A2K5XCF2_MANLE 108 125 USERPAT1 . . .
tDEEqAisrVLEaSiaEn
A0A2K5XCF2_MANLE 108 125 USERPAT1 . . .
tDEEqAisrVLEaSiaEn

A0A2K5XCF4_MANLE 108 125 USERPAT1 . . .
tDEEqAisrVLEaSiaEn
A0A2K5XCF4_MANLE 108 125 USERPAT1 . . .
tDEEqAisrVLEaSiaEn

A0A2K5XCH2_MANLE 116 133 USERPAT1 . . .
tDEEqAisrVLEaSiaEn
A0A2K5XCH2_MANLE 116 133 USERPAT1 . . .
tDEEqAisrVLEaSiaEn

A0A2K5ZAF0_MANLE 285 301 USERPAT1 . . .
gEDDmLqa.AVTmSleTv
A0A2K5ZAF0_MANLE 285 301 USERPAT1 . . .
gEDDmLqa.AVTmSleTv
A0A2K5ZAF0_MANLE 285 301 USERPAT1 . . .
gEDDmLqa.AVTmSleTv

A0A2K5ZAM9_MANLE 314 330 USERPAT1 . . .
gEDDmLqa.AVTmSleTv
A0A2K5ZAM9_MANLE 314 330 USERPAT1 . . .
gEDDmLqa.AVTmSleTv
A0A2K5ZAM9_MANLE 314 330 USERPAT1 . . .
gEDDmLqa.AVTmSleTv

A0A2K5ZAN4_MANLE 299 315 USERPAT1 . . .
gEDDmLqa.AVTmSleTv
A0A2K5ZAN4_MANLE 299 315 USERPAT1 . . .
gEDDmLqa.AVTmSleTv
A0A2K5ZAN4_MANLE 299 315 USERPAT1 . . .
gEDDmLqa.AVTmSleTv

A0A2K5ZAN9_MANLE 270 286 USERPAT1 . . .
gEDDmLqa.AVTmSleTv
A0A2K5ZAN9_MANLE 270 286 USERPAT1 . . .
gEDDmLqa.AVTmSleTv
A0A2K5ZAN9_MANLE 270 286 USERPAT1 . . .
gEDDmLqa.AVTmSleTv

A0A2K5ZAP4_MANLE 263 279 USERPAT1 . . .
gEDDmLqa.AVTmSleTv
A0A2K5ZAP4_MANLE 263 279 USERPAT1 . . .
gEDDmLqa.AVTmSleTv
A0A2K5ZAP4_MANLE 263 279 USERPAT1 . . .
gEDDmLqa.AVTmSleTv

A0A2K5ZAP6_MANLE 161 177 USERPAT1 . . .
gEDDmLqa.AVTmSleTv

A0A2K5ZAP6_MANLE 161 177 USERPAT1 . . .
gEDDmLqa.AVTmSleTv
A0A2K5ZAP6_MANLE 161 177 USERPAT1 . . .
gEDDmLqa.AVTmSleTv

A0A2K5ZAS2_MANLE 255 271 USERPAT1 . . .
gEDDmLqa.AVTmSleTv
A0A2K5ZAS2_MANLE 255 271 USERPAT1 . . .
gEDDmLqa.AVTmSleTv
A0A2K5ZAS2_MANLE 255 271 USERPAT1 . . .
gEDDmLqa.AVTmSleTv

A0A2K5ZAS5_MANLE 304 320 USERPAT1 . . .
gEDDmLqa.AVTmSleTv
A0A2K5ZAS5_MANLE 304 320 USERPAT1 . . .
gEDDmLqa.AVTmSleTv
A0A2K5ZAS5_MANLE 304 320 USERPAT1 . . .
gEDDmLqa.AVTmSleTv

A0A2K5ZHD8_MANLE 706 722 USERPAT1 . . .
sEEElLaa.VLEiSkrDa
A0A2K5ZHD8_MANLE 706 722 USERPAT1 . . .
sEEElLaa.VLEiSkrDa

A0A2K5ZHK6_MANLE 612 628 USERPAT1 . . .
sEEElLaa.VLEiSkrDa
A0A2K5ZHK6_MANLE 612 628 USERPAT1 . . .
sEEElLaa.VLEiSkrDa

A0A2K6C5A8_MACNE 314 330 USERPAT1 . . .
gEEDmLqa.AVTmSleTv
A0A2K6C5A8_MACNE 314 330 USERPAT1 . . .
gEEDmLqa.AVTmSleTv
A0A2K6C5A8_MACNE 314 330 USERPAT1 . . .
gEEDmLqa.AVTmSleTv

A0A2K6C5B8_MACNE 305 321 USERPAT1 . . .
gEEDmLqa.AVTmSleTv
A0A2K6C5B8_MACNE 305 321 USERPAT1 . . .
gEEDmLqa.AVTmSleTv
A0A2K6C5B8_MACNE 305 321 USERPAT1 . . .
gEEDmLqa.AVTmSleTv

A0A2K6C5F6_MACNE 297 313 USERPAT1 . . .
gEEDmLqa.AVTmSleTv
A0A2K6C5F6_MACNE 297 313 USERPAT1 . . .
gEEDmLqa.AVTmSleTv
A0A2K6C5F6_MACNE 297 313 USERPAT1 . . .
gEEDmLqa.AVTmSleTv

A0A2K6C5F9_MACNE 288 304 USERPAT1 . . .
gEEDmLqa.AVTmSleTv
A0A2K6C5F9_MACNE 288 304 USERPAT1 . . .
gEEDmLqa.AVTmSleTv

A0A2K6C5F9_MACNE 288 304 USERPAT1 . . .
gEEDmLqa.AVTmSleTv

A0A2K6CR70_MACNE 322 338 USERPAT1 . . .
gEEDmLqa.AVTmSleTv
A0A2K6CR70_MACNE 322 338 USERPAT1 . . .
gEEDmLqa.AVTmSleTv
A0A2K6CR70_MACNE 322 338 USERPAT1 . . .
gEEDmLqa.AVTmSleTv

A0A2K6CR89_MACNE 337 353 USERPAT1 . . .
gEEDmLqa.AVTmSleTv
A0A2K6CR89_MACNE 337 353 USERPAT1 . . .
gEEDmLqa.AVTmSleTv
A0A2K6CR89_MACNE 337 353 USERPAT1 . . .
gEEDmLqa.AVTmSleTv

A0A2K6CRA0_MACNE 286 302 USERPAT1 . . .
gEEDmLqa.AVTmSleTv
A0A2K6CRA0_MACNE 286 302 USERPAT1 . . .
gEEDmLqa.AVTmSleTv
A0A2K6CRA0_MACNE 286 302 USERPAT1 . . .
gEEDmLqa.AVTmSleTv

A0A2K6CRB9_MACNE 267 283 USERPAT1 . . .
gEEDmLqa.AVTmSleTv
A0A2K6CRB9_MACNE 267 283 USERPAT1 . . .
gEEDmLqa.AVTmSleTv
A0A2K6CRB9_MACNE 267 283 USERPAT1 . . .
gEEDmLqa.AVTmSleTv

A0A2K6CRD4_MACNE 282 298 USERPAT1 . . .
gEEDmLqa.AVTmSleTv
A0A2K6CRD4_MACNE 282 298 USERPAT1 . . .
gEEDmLqa.AVTmSleTv
A0A2K6CRD4_MACNE 282 298 USERPAT1 . . .
gEEDmLqa.AVTmSleTv

A0A2K6D0E5_MACNE 706 722 USERPAT1 . . .
sEEElLaa.VLGvSkrDa
A0A2K6D0E5_MACNE 706 722 USERPAT1 . . .
sEEElLaa.VLGvSkrDa

A0A2K6D0H3_MACNE 612 628 USERPAT1 . . .
sEEElLaa.VLGvSkrDa
A0A2K6D0H3_MACNE 612 628 USERPAT1 . . .
sEEElLaa.VLGvSkrDa

A0A2K6E0V0_MACNE 123 140 USERPAT1 . . .
tDEEqAisrVLEaSiaEn
A0A2K6E0V0_MACNE 123 140 USERPAT1 . . .
tDEEqAisrVLEaSiaEn

A0A2K6E0W2_MACNE 123 140 USERPAT1 . . .

tDEEqAisrVLEaSiaEn
A0A2K6E0W2_MACNE 123 140 USERPAT1 . . .
tDEEqAisrVLEaSiaEn

A0A2K6E0Y0_MACNE 123 140 USERPAT1 . . .
tDEEqAisrVLEaSiaEn
A0A2K6E0Y0_MACNE 123 140 USERPAT1 . . .
tDEEqAisrVLEaSiaEn

A0A2K6E0Y7_MACNE 116 133 USERPAT1 . . .
tDEEqAisrVLEaSiaEn
A0A2K6E0Y7_MACNE 116 133 USERPAT1 . . .
tDEEqAisrVLEaSiaEn

A0A2K6E0Z1_MACNE 123 140 USERPAT1 . . .
tDEEqAisrVLEaSiaEn
A0A2K6E0Z1_MACNE 123 140 USERPAT1 . . .
tDEEqAisrVLEaSiaEn

A0A2K6FS24_PROCO 573 589 USERPAT1 . . .
sEEElLaa.VLEiSktEt
A0A2K6FS24_PROCO 573 589 USERPAT1 . . .
sEEElLaa.VLEiSktEt

A0A2K6FS28_PROCO 647 663 USERPAT1 . . .
sEEElLaa.VLEiSktEt
A0A2K6FS28_PROCO 647 663 USERPAT1 . . .
sEEElLaa.VLEiSktEt

A0A2K6FYR8_PROCO 123 140 USERPAT1 . . .
tDEEqAisrVLEaSiaEn
A0A2K6FYR8_PROCO 123 140 USERPAT1 . . .
tDEEqAisrVLEaSiaEn

A0A2K6FYS1_PROCO 123 140 USERPAT1 . . .
tDEEqAisrVLEaSiaEn
A0A2K6FYS1_PROCO 123 140 USERPAT1 . . .
tDEEqAisrVLEaSiaEn

A0A2K6FYT1_PROCO 123 140 USERPAT1 . . .
tDEEqAisrVLEaSiaEn
A0A2K6FYT1_PROCO 123 140 USERPAT1 . . .
tDEEqAisrVLEaSiaEn

A0A2K6KTJ1_RHIBE 706 722 USERPAT1 . . .
sEEElLaa.VLEiSkrDa
A0A2K6KTJ1_RHIBE 706 722 USERPAT1 . . .
sEEElLaa.VLEiSkrDa

A0A2K6KTJ5_RHIBE 612 628 USERPAT1 . . .
sEEElLaa.VLEiSkrDa
A0A2K6KTJ5_RHIBE 612 628 USERPAT1 . . .
sEEElLaa.VLEiSkrDa

A0A2K6LPK2_RHIBE 123 140 USERPAT1 . . .
tDEEqAisrVLEaSiaEn
A0A2K6LPK2_RHIBE 123 140 USERPAT1 . . .
tDEEqAisrVLEaSiaEn

A0A2K6LPK3_RHIBE 123 140 USERPAT1 . . .
tDEEqAisrVLEaSiaEn
A0A2K6LPK3_RHIBE 123 140 USERPAT1 . . .
tDEEqAisrVLEaSiaEn

A0A2K6LPK5_RHIBE 116 133 USERPAT1 . . .
tDEEqAisrVLEaSiaEn
A0A2K6LPK5_RHIBE 116 133 USERPAT1 . . .
tDEEqAisrVLEaSiaEn

A0A2K6LPP5_RHIBE 123 140 USERPAT1 . . .
tDEEqAisrVLEaSiaEn
A0A2K6LPP5_RHIBE 123 140 USERPAT1 . . .
tDEEqAisrVLEaSiaEn

A0A2K6LPQ2_RHIBE 123 140 USERPAT1 . . .
tDEEqAisrVLEaSiaEn
A0A2K6LPQ2_RHIBE 123 140 USERPAT1 . . .
tDEEqAisrVLEaSiaEn

A0A2K6Q5D4_RHIRO 123 140 USERPAT1 . . .
tDEEqAisrVLEaSiaEn
A0A2K6Q5D4_RHIRO 123 140 USERPAT1 . . .
tDEEqAisrVLEaSiaEn

A0A2K6Q5D7_RHIRO 123 140 USERPAT1 . . .
tDEEqAisrVLEaSiaEn
A0A2K6Q5D7_RHIRO 123 140 USERPAT1 . . .
tDEEqAisrVLEaSiaEn

A0A2K6Q5E6_RHIRO 123 140 USERPAT1 . . .
tDEEqAisrVLEaSiaEn
A0A2K6Q5E6_RHIRO 123 140 USERPAT1 . . .
tDEEqAisrVLEaSiaEn

A0A2K6Q5E8_RHIRO 116 133 USERPAT1 . . .
tDEEqAisrVLEaSiaEn
A0A2K6Q5E8_RHIRO 116 133 USERPAT1 . . .
tDEEqAisrVLEaSiaEn

A0A2K6Q5F6_RHIRO 123 140 USERPAT1 . . .
tDEEqAisrVLEaSiaEn
A0A2K6Q5F6_RHIRO 123 140 USERPAT1 . . .
tDEEqAisrVLEaSiaEn

A0A2K6QIU0_RHIRO 257 273 USERPAT1 . . .
gEEDmLqa.AVTmSleTv
A0A2K6QIU0_RHIRO 257 273 USERPAT1 . . .
gEEDmLqa.AVTmSleTv

A0A2K6QIU0_RHIRO 257 273 USERPAT1 . . .
gEEDmLqa.AVTmSleTv

A0A2K6QIX4_RHIRO 278 294 USERPAT1 . . .
gEEDmLqa.AVTmSleTv
A0A2K6QIX4_RHIRO 278 294 USERPAT1 . . .
gEEDmLqa.AVTmSleTv
A0A2K6QIX4_RHIRO 278 294 USERPAT1 . . .
gEEDmLqa.AVTmSleTv

A0A2K6QIY3_RHIRO 312 328 USERPAT1 . . .
gEEDmLqa.AVTmSleTv
A0A2K6QIY3_RHIRO 312 328 USERPAT1 . . .
gEEDmLqa.AVTmSleTv
A0A2K6QIY3_RHIRO 312 328 USERPAT1 . . .
gEEDmLqa.AVTmSleTv

A0A2K6QIY4_RHIRO 339 355 USERPAT1 . . .
gEEDmLqa.AVTmSleTv
A0A2K6QIY4_RHIRO 339 355 USERPAT1 . . .
gEEDmLqa.AVTmSleTv
A0A2K6QIY4_RHIRO 339 355 USERPAT1 . . .
gEEDmLqa.AVTmSleTv

A0A2K6QIZ0_RHIRO 259 275 USERPAT1 . . .
gEEDmLqa.AVTmSleTv
A0A2K6QIZ0_RHIRO 259 275 USERPAT1 . . .
gEEDmLqa.AVTmSleTv
A0A2K6QIZ0_RHIRO 259 275 USERPAT1 . . .
gEEDmLqa.AVTmSleTv

A0A2K6RIY8_RHIRO 684 700 USERPAT1 . . .
sEEElLaa.VLEiSkrDa
A0A2K6RIY8_RHIRO 684 700 USERPAT1 . . .
sEEElLaa.VLEiSkrDa

A0A2K6RIZ5_RHIRO 706 722 USERPAT1 . . .
sEEElLaa.VLEiSkrDa
A0A2K6RIZ5_RHIRO 706 722 USERPAT1 . . .
sEEElLaa.VLEiSkrDa

A0A2K6RJ05_RHIRO 706 722 USERPAT1 . . .
sEEElLaa.VLEiSkrDa
A0A2K6RJ05_RHIRO 706 722 USERPAT1 . . .
sEEElLaa.VLEiSkrDa

A0A2K6S3S7_SAIBB 119 136 USERPAT1 . . .
tDEEqAisrVLEaSiaEn
A0A2K6S3S7_SAIBB 119 136 USERPAT1 . . .
tDEEqAisrVLEaSiaEn

A0A2K6S3T3_SAIBB 119 136 USERPAT1 . . .
tDEEqAisrVLEaSiaEn
A0A2K6S3T3_SAIBB 119 136 USERPAT1 . . .

tDEEqAisrVLEaSiaEn

A0A2K6S3W5_SAIBB 108 125 USERPAT1 . . .
tDEEqAisrVLEaSiaEn
A0A2K6S3W5_SAIBB 108 125 USERPAT1 . . .
tDEEqAisrVLEaSiaEn

A0A2K6S3X0_SAIBB 108 125 USERPAT1 . . .
tDEEqAisrVLEaSiaEn
A0A2K6S3X0_SAIBB 108 125 USERPAT1 . . .
tDEEqAisrVLEaSiaEn

A0A2K6SAB9_SAIBB 284 300 USERPAT1 . . .
sEEDmLqa.AVTmSleTv
A0A2K6SAB9_SAIBB 284 300 USERPAT1 . . .
sEEDmLqa.AVTmSleTv
A0A2K6SAB9_SAIBB 284 300 USERPAT1 . . .
sEEDmLqa.AVTmSleTv

A0A2K6SAC7_SAIBB 229 245 USERPAT1 . . .
sEEDmLqa.AVTmSleTv
A0A2K6SAC7_SAIBB 229 245 USERPAT1 . . .
sEEDmLqa.AVTmSleTv
A0A2K6SAC7_SAIBB 229 245 USERPAT1 . . .
sEEDmLqa.AVTmSleTv

A0A2K6SAD3_SAIBB 269 285 USERPAT1 . . .
sEEDmLqa.AVTmSleTv
A0A2K6SAD3_SAIBB 269 285 USERPAT1 . . .
sEEDmLqa.AVTmSleTv
A0A2K6SAD3_SAIBB 269 285 USERPAT1 . . .
sEEDmLqa.AVTmSleTv

A0A2K6SAE7_SAIBB 214 230 USERPAT1 . . .
sEEDmLqa.AVTmSleTv
A0A2K6SAE7_SAIBB 214 230 USERPAT1 . . .
sEEDmLqa.AVTmSleTv
A0A2K6SAE7_SAIBB 214 230 USERPAT1 . . .
sEEDmLqa.AVTmSleTv

A0A2K6SAG3_SAIBB 297 313 USERPAT1 . . .
sEEDmLqa.AVTmSleTv
A0A2K6SAG3_SAIBB 297 313 USERPAT1 . . .
sEEDmLqa.AVTmSleTv
A0A2K6SAG3_SAIBB 297 313 USERPAT1 . . .
sEEDmLqa.AVTmSleTv

A0A2P6TCJ9_CHLSO 52 68 USERPAT1 . . .
tEEEeLqr.ALEmSmaEa

A0A2R2MSQ0_LINUN 623 639 USERPAT1 . . .
sEEDqLqa.ALEmSlqEa
A0A2R2MSQ0_LINUN 623 639 USERPAT1 . . .
sEEDqLqa.ALEmSlqEa

A0A2R2MSQ0_LINUN 623 639 USERPAT1 . . .
sEEDqLqa.ALEmSlqEa

A0A2R2MSS4_LINUN 624 640 USERPAT1 . . .
sEEDqLqa.ALEmSlqEa
A0A2R2MSS4_LINUN 624 640 USERPAT1 . . .
sEEDqLqa.ALEmSlqEa
A0A2R2MSS4_LINUN 624 640 USERPAT1 . . .
sEEDqLqa.ALEmSlqEa

A0A2R8MPD6_CALJA 325 341 USERPAT1 . . .
sEEDmLqa.AVTmSleTv
A0A2R8MPD6_CALJA 325 341 USERPAT1 . . .
sEEDmLqa.AVTmSleTv
A0A2R8MPD6_CALJA 325 341 USERPAT1 . . .
sEEDmLqa.AVTmSleTv

A0A2R8N2N5_CALJA 119 136 USERPAT1 . . .
tDEEqAisrVLEaSiaEn
A0A2R8N2N5_CALJA 119 136 USERPAT1 . . .
tDEEqAisrVLEaSiaEn

A0A2R8P551_CALJA 289 305 USERPAT1 . . .
sEEDmLqa.AVTmSleTv
A0A2R8P551_CALJA 289 305 USERPAT1 . . .
sEEDmLqa.AVTmSleTv
A0A2R8P551_CALJA 289 305 USERPAT1 . . .
sEEDmLqa.AVTmSleTv

A0A2R8PDN5_CALJA 270 286 USERPAT1 . . .
sEEDmLqa.AVTmSleTv
A0A2R8PDN5_CALJA 270 286 USERPAT1 . . .
sEEDmLqa.AVTmSleTv
A0A2R8PDN5_CALJA 270 286 USERPAT1 . . .
sEEDmLqa.AVTmSleTv

A0A2R8Z9I3_PANPA 283 299 USERPAT1 . . .
sEEDmLqa.AVTmSleTv
A0A2R8Z9I3_PANPA 283 299 USERPAT1 . . .
sEEDmLqa.AVTmSleTv
A0A2R8Z9I3_PANPA 283 299 USERPAT1 . . .
sEEDmLqa.AVTmSleTv

A0A2R8Z9J0_PANPA 232 248 USERPAT1 . . .
sEEDmLqa.AVTmSleTv
A0A2R8Z9J0_PANPA 232 248 USERPAT1 . . .
sEEDmLqa.AVTmSleTv
A0A2R8Z9J0_PANPA 232 248 USERPAT1 . . .
sEEDmLqa.AVTmSleTv

A0A2R8ZCW5_PANPA 224 240 USERPAT1 . . .
qDEEdFqr.ALElSrqEt
A0A2R8ZCW5_PANPA 224 240 USERPAT1 . . .
qDEEdFqr.ALElSrqEt

A0A2R8ZDP2_PANPA 281 297 USERPAT1 . . .
sEEDmLqa.AVTmSleTv
A0A2R8ZDP2_PANPA 281 297 USERPAT1 . . .
sEEDmLqa.AVTmSleTv
A0A2R8ZDP2_PANPA 281 297 USERPAT1 . . .
sEEDmLqa.AVTmSleTv

A0A2R8ZGA2_PANPA 268 284 USERPAT1 . . .
sEEDmLqa.AVTmSleTv
A0A2R8ZGA2_PANPA 268 284 USERPAT1 . . .
sEEDmLqa.AVTmSleTv
A0A2R8ZGA2_PANPA 268 284 USERPAT1 . . .
sEEDmLqa.AVTmSleTv

A0A2R9A530_PANPA 704 720 USERPAT1 . . .
sEEElLaa.VLEiSkrDa
A0A2R9A530_PANPA 704 720 USERPAT1 . . .
sEEElLaa.VLEiSkrDa

A0A2R9A6A4_PANPA 704 720 USERPAT1 . . .
sEEElLaa.VLEiSkrDa
A0A2R9A6A4_PANPA 704 720 USERPAT1 . . .
sEEElLaa.VLEiSkrDa

A0A2R9A9T7_PANPA 610 626 USERPAT1 . . .
sEEElLaa.VLEiSkrDa
A0A2R9A9T7_PANPA 610 626 USERPAT1 . . .
sEEElLaa.VLEiSkrDa

A0A2R9BV93_PANPA 163 180 USERPAT1 . . .
tDEEqAisrVLEaSiaEn
A0A2R9BV93_PANPA 163 180 USERPAT1 . . .
tDEEqAisrVLEaSiaEn

A0A2R9BVF7_PANPA 108 125 USERPAT1 . . .
tDEEqAisrVLEaSiaEn
A0A2R9BVF7_PANPA 108 125 USERPAT1 . . .
tDEEqAisrVLEaSiaEn

A0A2R9BVY5_PANPA 172 189 USERPAT1 . . .
tDEEqAisrVLEaSiaEn
A0A2R9BVY5_PANPA 172 189 USERPAT1 . . .
tDEEqAisrVLEaSiaEn

A0A2R9BYP4_PANPA 163 180 USERPAT1 . . .
tDEEqAisrVLEaSiaEn
A0A2R9BYP4_PANPA 163 180 USERPAT1 . . .
tDEEqAisrVLEaSiaEn

A0A2R9BYT7_PANPA 116 133 USERPAT1 . . .
tDEEqAisrVLEaSiaEn
A0A2R9BYT7_PANPA 116 133 USERPAT1 . . .
tDEEqAisrVLEaSiaEn

A0A2R9BYV0_PANPA 108 125 USERPAT1 . . .
tDEEqAisrVLEaSiaEn
A0A2R9BYV0_PANPA 108 125 USERPAT1 . . .
tDEEqAisrVLEaSiaEn

A0A2R9C547_PANPA 116 133 USERPAT1 . . .
tDEEqAisrVLEaSiaEn
A0A2R9C547_PANPA 116 133 USERPAT1 . . .
tDEEqAisrVLEaSiaEn

A0A2U3VI69_ODORO 319 335 USERPAT1 . . .
sEEDmLqa.AVTmSleTv
A0A2U3VI69_ODORO 319 335 USERPAT1 . . .
sEEDmLqa.AVTmSleTv
A0A2U3VI69_ODORO 319 335 USERPAT1 . . .
sEEDmLqa.AVTmSleTv

A0A2U3VI71_ODORO 283 299 USERPAT1 . . .
sEEDmLqa.AVTmSleTv
A0A2U3VI71_ODORO 283 299 USERPAT1 . . .
sEEDmLqa.AVTmSleTv
A0A2U3VI71_ODORO 283 299 USERPAT1 . . .
sEEDmLqa.AVTmSleTv

A0A2U3VI74_ODORO 334 350 USERPAT1 . . .
sEEDmLqa.AVTmSleTv
A0A2U3VI74_ODORO 334 350 USERPAT1 . . .
sEEDmLqa.AVTmSleTv
A0A2U3VI74_ODORO 334 350 USERPAT1 . . .
sEEDmLqa.AVTmSleTv

A0A2U3W3W0_ODORO 704 720 USERPAT1 . . .
sEEElLaa.VLEmSkrEa
A0A2U3W3W0_ODORO 704 720 USERPAT1 . . .
sEEElLaa.VLEmSkrEa
A0A2U3W3W0_ODORO 704 720 USERPAT1 . . .
sEEElLaa.VLEmSkrEa

A0A2U3WNK6_ODORO 123 140 USERPAT1 . . .
tDEEqAisrVLEaSiaEn
A0A2U3WNK6_ODORO 123 140 USERPAT1 . . .
tDEEqAisrVLEaSiaEn

A0A2U3WNL0_ODORO 123 140 USERPAT1 . . .
tDEEqAisrVLEaSiaEn
A0A2U3WNL0_ODORO 123 140 USERPAT1 . . .
tDEEqAisrVLEaSiaEn

A0A2U3WNL2_ODORO 123 140 USERPAT1 . . .
tDEEqAisrVLEaSiaEn
A0A2U3WNL2_ODORO 123 140 USERPAT1 . . .
tDEEqAisrVLEaSiaEn

A0A2U3YG82_LEPWE 123 140 USERPAT1 . . .
tDEEqAisrVLEaSiaEn
A0A2U3YG82_LEPWE 123 140 USERPAT1 . . .
tDEEqAisrVLEaSiaEn

A0A2U3YG86_LEPWE 123 140 USERPAT1 . . .
tDEEqAisrVLEaSiaEn
A0A2U3YG86_LEPWE 123 140 USERPAT1 . . .
tDEEqAisrVLEaSiaEn

A0A2U3YG90_LEPWE 123 140 USERPAT1 . . .
tDEEqAisrVLEaSiaEn
A0A2U3YG90_LEPWE 123 140 USERPAT1 . . .
tDEEqAisrVLEaSiaEn

A0A2U3YYM3_LEPWE 619 635 USERPAT1 . . .
sEEElLaa.VLEmSkrEa
A0A2U3YYM3_LEPWE 619 635 USERPAT1 . . .
sEEElLaa.VLEmSkrEa
A0A2U3YYM3_LEPWE 619 635 USERPAT1 . . .
sEEElLaa.VLEmSkrEa

A0A2U3Z9T4_ODORO 327 343 USERPAT1 . . .
sEEDmLqa.AVTmSleTv
A0A2U3Z9T4_ODORO 327 343 USERPAT1 . . .
sEEDmLqa.AVTmSleTv
A0A2U3Z9T4_ODORO 327 343 USERPAT1 . . .
sEEDmLqa.AVTmSleTv

A0A2U3ZAS3_ODORO 325 341 USERPAT1 . . .
sEEDmLqa.AVTmSleTv
A0A2U3ZAS3_ODORO 325 341 USERPAT1 . . .
sEEDmLqa.AVTmSleTv
A0A2U3ZAS3_ODORO 325 341 USERPAT1 . . .
sEEDmLqa.AVTmSleTv

A0A2U4A049_TURTR 324 340 USERPAT1 . . .
sEEDmLqa.AVTmSleTv
A0A2U4A049_TURTR 324 340 USERPAT1 . . .
sEEDmLqa.AVTmSleTv
A0A2U4A049_TURTR 324 340 USERPAT1 . . .
sEEDmLqa.AVTmSleTv

A0A2U4A057_TURTR 334 350 USERPAT1 . . .
sEEDmLqa.AVTmSleTv
A0A2U4A057_TURTR 334 350 USERPAT1 . . .
sEEDmLqa.AVTmSleTv
A0A2U4A057_TURTR 334 350 USERPAT1 . . .
sEEDmLqa.AVTmSleTv

A0A2U4A0F2_TURTR 273 289 USERPAT1 . . .
sEEDmLqa.AVTmSleTv
A0A2U4A0F2_TURTR 273 289 USERPAT1 . . .
sEEDmLqa.AVTmSleTv

A0A2U4A0F2_TURTR 273 289 USERPAT1 . . .
sEEDmLqa.AVTmSleTv

A0A2U4AAQ4_TURTR 123 140 USERPAT1 . . .
tDEEqAisrVLEaSiaEn
A0A2U4AAQ4_TURTR 123 140 USERPAT1 . . .
tDEEqAisrVLEaSiaEn

A0A2U4AAU2_TURTR 123 140 USERPAT1 . . .
tDEEqAisrVLEaSiaEn
A0A2U4AAU2_TURTR 123 140 USERPAT1 . . .
tDEEqAisrVLEaSiaEn

A0A2U4AB23_TURTR 123 140 USERPAT1 . . .
tDEEqAisrVLEaSiaEn
A0A2U4AB23_TURTR 123 140 USERPAT1 . . .
tDEEqAisrVLEaSiaEn

A0A2U4BMR5_TURTR 706 722 USERPAT1 . . .
sEEElLaa.VLEiSkrEa
A0A2U4BMR5_TURTR 706 722 USERPAT1 . . .
sEEElLaa.VLEiSkrEa
A0A2U4BMR5_TURTR 706 722 USERPAT1 . . .
sEEElLaa.VLEiSkrEa

A0A2U9C875_SCOMX 686 702 USERPAT1 . . .
nDDEmLaa.VLEmSrqEa
A0A2U9C875_SCOMX 686 702 USERPAT1 . . .
nDDEmLaa.VLEmSrqEa
A0A2U9C875_SCOMX 686 702 USERPAT1 . . .
nDDEmLaa.VLEmSrqEa

A0A2Y9DB53_TRIMA 124 141 USERPAT1 . . .
tDEEqAisrVLEaSiaEn
A0A2Y9DB53_TRIMA 124 141 USERPAT1 . . .
tDEEqAisrVLEaSiaEn

A0A2Y9DKN0_TRIMA 706 722 USERPAT1 . . .
sEEElVaa.VLEiSkrEt
A0A2Y9DKN0_TRIMA 706 722 USERPAT1 . . .
sEEElVaa.VLEiSkrEt

A0A2Y9ELX6_PHYMC 323 339 USERPAT1 . . .
sEEDmLqa.AVTmSleTv
A0A2Y9ELX6_PHYMC 323 339 USERPAT1 . . .
sEEDmLqa.AVTmSleTv
A0A2Y9ELX6_PHYMC 323 339 USERPAT1 . . .
sEEDmLqa.AVTmSleTv

A0A2Y9EM29_PHYMC 324 340 USERPAT1 . . .
sEEDmLqa.AVTmSleTv
A0A2Y9EM29_PHYMC 324 340 USERPAT1 . . .
sEEDmLqa.AVTmSleTv
A0A2Y9EM29_PHYMC 324 340 USERPAT1 . . .

sEEDmLqa.AVTmSleTv

A0A2Y9ENB0_PHYMC 309 325 USERPAT1 . . .
sEEDmLqa.AVTmSleTv
A0A2Y9ENB0_PHYMC 309 325 USERPAT1 . . .
sEEDmLqa.AVTmSleTv
A0A2Y9ENB0_PHYMC 309 325 USERPAT1 . . .
sEEDmLqa.AVTmSleTv

A0A2Y9FV85_TRIMA 124 141 USERPAT1 . . .
tDEEqAisrVLEaSiaEn
A0A2Y9FV85_TRIMA 124 141 USERPAT1 . . .
tDEEqAisrVLEaSiaEn

A0A2Y9IQI5_ENHLU 683 699 USERPAT1 . . .
sEEElLaa.VLEmSkrEa
A0A2Y9IQI5_ENHLU 683 699 USERPAT1 . . .
sEEElLaa.VLEmSkrEa
A0A2Y9IQI5_ENHLU 683 699 USERPAT1 . . .
sEEElLaa.VLEmSkrEa

A0A2Y9IRQ7_ENHLU 705 721 USERPAT1 . . .
sEEElLaa.VLEmSkrEa
A0A2Y9IRQ7_ENHLU 705 721 USERPAT1 . . .
sEEElLaa.VLEmSkrEa
A0A2Y9IRQ7_ENHLU 705 721 USERPAT1 . . .
sEEElLaa.VLEmSkrEa

A0A2Y9JME8_ENHLU 123 140 USERPAT1 . . .
tDEEqAisrVLEaSiaEn
A0A2Y9JME8_ENHLU 123 140 USERPAT1 . . .
tDEEqAisrVLEaSiaEn

A0A2Y9JMF9_ENHLU 123 140 USERPAT1 . . .
tDEEqAisrVLEaSiaEn
A0A2Y9JMF9_ENHLU 123 140 USERPAT1 . . .
tDEEqAisrVLEaSiaEn

A0A2Y9JML0_ENHLU 326 342 USERPAT1 . . .
sEEDmLqa.AVTmSleTv
A0A2Y9JML0_ENHLU 326 342 USERPAT1 . . .
sEEDmLqa.AVTmSleTv
A0A2Y9JML0_ENHLU 326 342 USERPAT1 . . .
sEEDmLqa.AVTmSleTv

A0A2Y9JSS8_ENHLU 329 345 USERPAT1 . . .
sEEDmLqa.AVTmSleTv
A0A2Y9JSS8_ENHLU 329 345 USERPAT1 . . .
sEEDmLqa.AVTmSleTv
A0A2Y9JSS8_ENHLU 329 345 USERPAT1 . . .
sEEDmLqa.AVTmSleTv

A0A2Y9MM93_DELLE 706 722 USERPAT1 . . .
sEEElLaa.VLEiSkrEa

A0A2Y9MM93_DELLE 706 722 USERPAT1 . . .
sEEElLaa.VLEiSkrEa
A0A2Y9MM93_DELLE 706 722 USERPAT1 . . .
sEEElLaa.VLEiSkrEa

A0A2Y9MN29_DELLE 684 700 USERPAT1 . . .
sEEElLaa.VLEiSkrEa
A0A2Y9MN29_DELLE 684 700 USERPAT1 . . .
sEEElLaa.VLEiSkrEa
A0A2Y9MN29_DELLE 684 700 USERPAT1 . . .
sEEElLaa.VLEiSkrEa

A0A2Y9MWH8_DELLE 706 722 USERPAT1 . . .
sEEElLaa.VLEiSkrEa
A0A2Y9MWH8_DELLE 706 722 USERPAT1 . . .
sEEElLaa.VLEiSkrEa
A0A2Y9MWH8_DELLE 706 722 USERPAT1 . . .
sEEElLaa.VLEiSkrEa

A0A2Y9NAE4_DELLE 123 140 USERPAT1 . . .
tDEEqAisrVLEaSiaEn
A0A2Y9NAE4_DELLE 123 140 USERPAT1 . . .
tDEEqAisrVLEaSiaEn

A0A2Y9NCV4_DELLE 322 338 USERPAT1 . . .
sEEDmLqa.AVTmSleTv
A0A2Y9NCV4_DELLE 322 338 USERPAT1 . . .
sEEDmLqa.AVTmSleTv
A0A2Y9NCV4_DELLE 322 338 USERPAT1 . . .
sEEDmLqa.AVTmSleTv

A0A2Y9NG79_DELLE 271 287 USERPAT1 . . .
sEEDmLqa.AVTmSleTv
A0A2Y9NG79_DELLE 271 287 USERPAT1 . . .
sEEDmLqa.AVTmSleTv
A0A2Y9NG79_DELLE 271 287 USERPAT1 . . .
sEEDmLqa.AVTmSleTv

A0A2Y9NKV4_DELLE 143 159 USERPAT1 . . .
sEEDmLqa.AVTmSleTv
A0A2Y9NKV4_DELLE 143 159 USERPAT1 . . .
sEEDmLqa.AVTmSleTv
A0A2Y9NKV4_DELLE 143 159 USERPAT1 . . .
sEEDmLqa.AVTmSleTv

A0A2Y9QGD7_TRIMA 152 169 USERPAT1 . . .
tDEEqAisrVLEaSiaEn
A0A2Y9QGD7_TRIMA 152 169 USERPAT1 . . .
tDEEqAisrVLEaSiaEn

A0A2Y9QLT2_TRIMA 57 74 USERPAT1 . . .
tDEEqAisrVLEaSiaEn
A0A2Y9QLT2_TRIMA 57 74 USERPAT1 . . .
tDEEqAisrVLEaSiaEn

A0A2Y9SEV2_PHYMC 123 140 USERPAT1 . . .
tDEEqAisrVLEaSiaEn
A0A2Y9SEV2_PHYMC 123 140 USERPAT1 . . .
tDEEqAisrVLEaSiaEn

A0A2Y9SHU5_PHYMC 123 140 USERPAT1 . . .
tDEEqAisrVLEaSiaEn
A0A2Y9SHU5_PHYMC 123 140 USERPAT1 . . .
tDEEqAisrVLEaSiaEn

A0A2Y9SNP8_PHYMC 684 700 USERPAT1 . . .
sEEElLaa.VLEiSkrEa
A0A2Y9SNP8_PHYMC 684 700 USERPAT1 . . .
sEEElLaa.VLEiSkrEa
A0A2Y9SNP8_PHYMC 684 700 USERPAT1 . . .
sEEElLaa.VLEiSkrEa

A0A2Y9SS61_PHYMC 684 700 USERPAT1 . . .
sEEElLaa.VLEiSkrEa
A0A2Y9SS61_PHYMC 684 700 USERPAT1 . . .
sEEElLaa.VLEiSkrEa
A0A2Y9SS61_PHYMC 684 700 USERPAT1 . . .
sEEElLaa.VLEiSkrEa

A0A2Y9SUT9_PHYMC 706 722 USERPAT1 . . .
sEEElLaa.VLEiSkrEa
A0A2Y9SUT9_PHYMC 706 722 USERPAT1 . . .
sEEElLaa.VLEiSkrEa
A0A2Y9SUT9_PHYMC 706 722 USERPAT1 . . .
sEEElLaa.VLEiSkrEa

A0A2Y9SWY0_PHYMC 706 722 USERPAT1 . . .
sEEElLaa.VLEiSkrEa
A0A2Y9SWY0_PHYMC 706 722 USERPAT1 . . .
sEEElLaa.VLEiSkrEa
A0A2Y9SWY0_PHYMC 706 722 USERPAT1 . . .
sEEElLaa.VLEiSkrEa

A0A337RUH1_FELCA 123 140 USERPAT1 . . .
tDEEqAisrVLEaSiaEn
A0A337RUH1_FELCA 123 140 USERPAT1 . . .
tDEEqAisrVLEaSiaEn

A0A337S7J4_FELCA 123 140 USERPAT1 . . .
tDEEqAisrVLEaSiaEn
A0A337S7J4_FELCA 123 140 USERPAT1 . . .
tDEEqAisrVLEaSiaEn

A0A337S8J2_FELCA 274 290 USERPAT1 . . .
sEEDmLqa.AVTmSleVv
A0A337S8J2_FELCA 274 290 USERPAT1 . . .
sEEDmLqa.AVTmSleVv
A0A337S8J2_FELCA 274 290 USERPAT1 . . .

sEEDmLqa.AVTmSleVv

A0A337SFZ6_FELCA 683 699 USERPAT1 . . .
sEEElLaa.VLEiSkrEa
A0A337SFZ6_FELCA 683 699 USERPAT1 . . .
sEEElLaa.VLEiSkrEa
A0A337SFZ6_FELCA 683 699 USERPAT1 . . .
sEEElLaa.VLEiSkrEa

A0A337SHM7_FELCA 123 140 USERPAT1 . . .
tDEEqAisrVLEaSiaEn
A0A337SHM7_FELCA 123 140 USERPAT1 . . .
tDEEqAisrVLEaSiaEn

A0A337SMY3_FELCA 255 271 USERPAT1 . . .
sEEDmLqa.AVTmSleVv
A0A337SMY3_FELCA 255 271 USERPAT1 . . .
sEEDmLqa.AVTmSleVv
A0A337SMY3_FELCA 255 271 USERPAT1 . . .
sEEDmLqa.AVTmSleVv

A0A337SN16_FELCA 310 326 USERPAT1 . . .
sEEDmLqa.AVTmSleVv
A0A337SN16_FELCA 310 326 USERPAT1 . . .
sEEDmLqa.AVTmSleVv
A0A337SN16_FELCA 310 326 USERPAT1 . . .
sEEDmLqa.AVTmSleVv

A0A337SPY1_FELCA 270 286 USERPAT1 . . .
sEEDmLqa.AVTmSleVv
A0A337SPY1_FELCA 270 286 USERPAT1 . . .
sEEDmLqa.AVTmSleVv
A0A337SPY1_FELCA 270 286 USERPAT1 . . .
sEEDmLqa.AVTmSleVv

A0A337SSU9_FELCA 150 167 USERPAT1 . . .
tDEEqAisrVLEaSiaEn
A0A337SSU9_FELCA 150 167 USERPAT1 . . .
tDEEqAisrVLEaSiaEn

A0A340WV50_LIPVE 706 722 USERPAT1 . . .
sEEElLaa.VLEiSkrEa
A0A340WV50_LIPVE 706 722 USERPAT1 . . .
sEEElLaa.VLEiSkrEa
A0A340WV50_LIPVE 706 722 USERPAT1 . . .
sEEElLaa.VLEiSkrEa

A0A340WZ65_LIPVE 123 140 USERPAT1 . . .
tDEEqAisrVLEaSiaEn
A0A340WZ65_LIPVE 123 140 USERPAT1 . . .
tDEEqAisrVLEaSiaEn

A0A340X0E7_LIPVE 330 346 USERPAT1 . . .
sEEDmLqa.AVTmSleTv

A0A340X0E7_LIPVE 330 346 USERPAT1 . . .
sEEDmLqa.AVTmSleTv
A0A340X0E7_LIPVE 330 346 USERPAT1 . . .
sEEDmLqa.AVTmSleTv

A0A340X0F2_LIPVE 260 276 USERPAT1 . . .
sEEDmLqa.AVTmSleTv
A0A340X0F2_LIPVE 260 276 USERPAT1 . . .
sEEDmLqa.AVTmSleTv
A0A340X0F2_LIPVE 260 276 USERPAT1 . . .
sEEDmLqa.AVTmSleTv

A0A340X338_LIPVE 141 158 USERPAT1 . . .
tDEEqAisrVLEaSiaEn
A0A340X338_LIPVE 141 158 USERPAT1 . . .
tDEEqAisrVLEaSiaEn

A0A340X484_LIPVE 315 331 USERPAT1 . . .
sEEDmLqa.AVTmSleTv
A0A340X484_LIPVE 315 331 USERPAT1 . . .
sEEDmLqa.AVTmSleTv
A0A340X484_LIPVE 315 331 USERPAT1 . . .
sEEDmLqa.AVTmSleTv

A0A340X644_LIPVE 326 342 USERPAT1 . . .
sEEDmLqa.AVTmSleTv
A0A340X644_LIPVE 326 342 USERPAT1 . . .
sEEDmLqa.AVTmSleTv
A0A340X644_LIPVE 326 342 USERPAT1 . . .
sEEDmLqa.AVTmSleTv

A0A340X648_LIPVE 279 295 USERPAT1 . . .
sEEDmLqa.AVTmSleTv
A0A340X648_LIPVE 279 295 USERPAT1 . . .
sEEDmLqa.AVTmSleTv
A0A340X648_LIPVE 279 295 USERPAT1 . . .
sEEDmLqa.AVTmSleTv

A0A340X6S4_LIPVE 325 341 USERPAT1 . . .
sEEDmLqa.AVTmSleTv
A0A340X6S4_LIPVE 325 341 USERPAT1 . . .
sEEDmLqa.AVTmSleTv
A0A340X6S4_LIPVE 325 341 USERPAT1 . . .
sEEDmLqa.AVTmSleTv

A0A340X6S9_LIPVE 275 291 USERPAT1 . . .
sEEDmLqa.AVTmSleTv
A0A340X6S9_LIPVE 275 291 USERPAT1 . . .
sEEDmLqa.AVTmSleTv
A0A340X6S9_LIPVE 275 291 USERPAT1 . . .
sEEDmLqa.AVTmSleTv

A0A384AKT3_BALAS 706 722 USERPAT1 . . .
sEEElLaa.VLEiSktEa

A0A384AKT3_BALAS 706 722 USERPAT1 . . .
sEEElLaa.VLEiSktEa

A0A384AL14_BALAS 89 105 USERPAT1 . . .
sEDEaLqr.ALElSlaEa

A0A384C1U5_URSMA 243 259 USERPAT1 . . .
sEDEaLqr.ALElSlaEn

A0A384C2P1_URSMA 705 721 USERPAT1 . . .
sEEElLaa.VLEmSkrEs
A0A384C2P1_URSMA 705 721 USERPAT1 . . .
sEEElLaa.VLEmSkrEs
A0A384C2P1_URSMA 705 721 USERPAT1 . . .
sEEElLaa.VLEmSkrEs

A0A384CPI0_URSMA 346 362 USERPAT1 . . .
sEEDmLqa.AVTlSleTv
A0A384CPI0_URSMA 346 362 USERPAT1 . . .
sEEDmLqa.AVTlSleTv
A0A384CPI0_URSMA 346 362 USERPAT1 . . .
sEEDmLqa.AVTlSleTv

A0A3B1KDQ4_ASTMX 664 680 USERPAT1 . . .
nDDEmLaa.VLEmSrqEa
A0A3B1KDQ4_ASTMX 664 680 USERPAT1 . . .
nDDEmLaa.VLEmSrqEa
A0A3B1KDQ4_ASTMX 664 680 USERPAT1 . . .
nDDEmLaa.VLEmSrqEa

A0A3B3DMP5_ORYME 96 113 USERPAT1 . . .
tDEEqAisrVLEaSiaEn
A0A3B3DMP5_ORYME 96 113 USERPAT1 . . .
tDEEqAisrVLEaSiaEn

A0A3B3DZF9_ORYME 680 697 USERPAT1 . . .
iDDDeMlaaVLEmSrqEa
A0A3B3DZF9_ORYME 680 697 USERPAT1 . . .
iDDDeMlaaVLEmSrqEa
A0A3B3DZF9_ORYME 680 697 USERPAT1 . . .
iDDDeMlaaVLEmSrqEa

A0A3B3RSL1_9TELE 116 133 USERPAT1 . . .
tDEEqAisrVLEaSiaEn
A0A3B3RSL1_9TELE 116 133 USERPAT1 . . .
tDEEqAisrVLEaSiaEn

A0A3B3RSV1_9TELE 122 139 USERPAT1 . . .
tDEEqAisrVLEaSiaEn
A0A3B3RSV1_9TELE 122 139 USERPAT1 . . .
tDEEqAisrVLEaSiaEn

A0A3B3RV06_9TELE 116 133 USERPAT1 . . .
tDEEqAisrVLEaSiaEn

A0A3B3RV06_9TELE 116 133 USERPAT1 . . .
tDEEqAisrVLEaSiaEn

A0A3B3VXF7_9TELE 680 696 USERPAT1 . . .
nDDEmLaa.VLEmSreEa
A0A3B3VXF7_9TELE 680 696 USERPAT1 . . .
nDDEmLaa.VLEmSreEa
A0A3B3VXF7_9TELE 680 696 USERPAT1 . . .
nDDEmLaa.VLEmSreEa

A0A3B3WZ97_9TELE 680 696 USERPAT1 . . .
nDDEmLaa.VLEmSrqEa
A0A3B3WZ97_9TELE 680 696 USERPAT1 . . .
nDDEmLaa.VLEmSrqEa
A0A3B3WZ97_9TELE 680 696 USERPAT1 . . .
nDDEmLaa.VLEmSrqEa

A0A3B4DWK9_PYGNA 674 690 USERPAT1 . . .
nDDEmLaa.VLEmSrhDt
A0A3B4DWK9_PYGNA 674 690 USERPAT1 . . .
nDDEmLaa.VLEmSrhDt
A0A3B4DWK9_PYGNA 674 690 USERPAT1 . . .
nDDEmLaa.VLEmSrhDt

A0A3B4DXK6_PYGNA 675 691 USERPAT1 . . .
nDDEmLaa.VLEmSrhDt
A0A3B4DXK6_PYGNA 675 691 USERPAT1 . . .
nDDEmLaa.VLEmSrhDt
A0A3B4DXK6_PYGNA 675 691 USERPAT1 . . .
nDDEmLaa.VLEmSrhDt

A0A3B4G0S9_9CICH 687 703 USERPAT1 . . .
nDDEmLaa.VLEiSrqEa
A0A3B4G0S9_9CICH 687 703 USERPAT1 . . .
nDDEmLaa.VLEiSrqEa
A0A3B4G0S9_9CICH 687 703 USERPAT1 . . .
nDDEmLaa.VLEiSrqEa

A0A3B4TVV6_SERDU 685 701 USERPAT1 . . .
nDDEmLaa.VLEmSrqEa
A0A3B4TVV6_SERDU 685 701 USERPAT1 . . .
nDDEmLaa.VLEmSrqEa
A0A3B4TVV6_SERDU 685 701 USERPAT1 . . .
nDDEmLaa.VLEmSrqEa

A0A3B4Y2G5_SERLL 685 701 USERPAT1 . . .
nDDEmLaa.VLEmSrqEa
A0A3B4Y2G5_SERLL 685 701 USERPAT1 . . .
nDDEmLaa.VLEmSrqEa
A0A3B4Y2G5_SERLL 685 701 USERPAT1 . . .
nDDEmLaa.VLEmSrqEa

A0A3B5AJH2_9TELE 687 703 USERPAT1 . . .
nDDEmLaa.VLEmSrqEa

A0A3B5AJH2_9TELE 687 703 USERPAT1 . . .
nDDEmLaa.VLEmSrqEa
A0A3B5AJH2_9TELE 687 703 USERPAT1 . . .
nDDEmLaa.VLEmSrqEa

A0A3L8SJU7_CHLGU 126 143 USERPAT1 . . .
tDEEqAisrVLEaSiaEn
A0A3L8SJU7_CHLGU 126 143 USERPAT1 . . .
tDEEqAisrVLEaSiaEn

A0A3L8SNF6_CHLGU 714 730 USERPAT1 . . .
sEDElLaa.VLEiSkrEa
A0A3L8SNF6_CHLGU 714 730 USERPAT1 . . .
sEDElLaa.VLEiSkrEa
A0A3L8SNF6_CHLGU 714 730 USERPAT1 . . .
sEDElLaa.VLEiSkrEa

A0A3M0L0W4_HIRRU 627 643 USERPAT1 . . .
sEDElLaa.VLEiSkrEa
A0A3M0L0W4_HIRRU 627 643 USERPAT1 . . .
sEDElLaa.VLEiSkrEa
A0A3M0L0W4_HIRRU 627 643 USERPAT1 . . .
sEDElLaa.VLEiSkrEa

A0A3M9YCH6_9PEZI 1166 1182 USERPAT1 . . .
dDDEdFkr.ALEaSkiPs
A0A3M9YCH6_9PEZI 1166 1182 USERPAT1 . . .
dDDEdFkr.ALEaSkiPs
A0A3M9YCH6_9PEZI 1166 1182 USERPAT1 . . .
dDDEdFkr.ALEaSkiPs

A0A3P8NBI3_ASTCA 96 113 USERPAT1 . . .
tDEEqAisrVLEaSiaEn
A0A3P8NBI3_ASTCA 96 113 USERPAT1 . . .
tDEEqAisrVLEaSiaEn

A0A3P8NFM1_ASTCA 621 637 USERPAT1 . . .
nDDEmLaa.VLEiSrqEa
A0A3P8NFM1_ASTCA 621 637 USERPAT1 . . .
nDDEmLaa.VLEiSrqEa
A0A3P8NFM1_ASTCA 621 637 USERPAT1 . . .
nDDEmLaa.VLEiSrqEa

A0A3P8S1N9_AMPPE 625 641 USERPAT1 . . .
nDDEmLaa.VLEmSrqEa
A0A3P8S1N9_AMPPE 625 641 USERPAT1 . . .
nDDEmLaa.VLEmSrqEa
A0A3P8S1N9_AMPPE 625 641 USERPAT1 . . .
nDDEmLaa.VLEmSrqEa

A0A3P8S300_AMPPE 687 703 USERPAT1 . . .
nDDEmLaa.VLEmSrqEa
A0A3P8S300_AMPPE 687 703 USERPAT1 . . .
nDDEmLaa.VLEmSrqEa

A0A3P8S300_AMPPE 687 703 USERPAT1 . . .
nDDEmLaa.VLEmSrqEa

A0A3P8WGD3_CYNSE 653 669 USERPAT1 . . .
nDDEmLaa.VLEmSrrEa
A0A3P8WGD3_CYNSE 653 669 USERPAT1 . . .
nDDEmLaa.VLEmSrrEa

A0A3P8Y9G8_ESOLU 681 697 USERPAT1 . . .
nDEDmLaa.VLEmSrqDa
A0A3P8Y9G8_ESOLU 681 697 USERPAT1 . . .
nDEDmLaa.VLEmSrqDa
A0A3P8Y9G8_ESOLU 681 697 USERPAT1 . . .
nDEDmLaa.VLEmSrqDa

A0A3P9A0L5_ESOLU 641 657 USERPAT1 . . .
nDEDmLaa.VLEmSrqDa
A0A3P9A0L5_ESOLU 641 657 USERPAT1 . . .
nDEDmLaa.VLEmSrqDa
A0A3P9A0L5_ESOLU 641 657 USERPAT1 . . .
nDEDmLaa.VLEmSrqDa

A0A3P9CAX5_9CICH 686 702 USERPAT1 . . .
nDDEmLaa.VLEiSrqEa
A0A3P9CAX5_9CICH 686 702 USERPAT1 . . .
nDDEmLaa.VLEiSrqEa
A0A3P9CAX5_9CICH 686 702 USERPAT1 . . .
nDDEmLaa.VLEiSrqEa

A0A3P9MUX0_POERE 683 699 USERPAT1 . . .
nDDEmLaa.VLEmSrqEa
A0A3P9MUX0_POERE 683 699 USERPAT1 . . .
nDDEmLaa.VLEmSrqEa
A0A3P9MUX0_POERE 683 699 USERPAT1 . . .
nDDEmLaa.VLEmSrqEa

A0A3Q0CKB2_MESAU 682 698 USERPAT1 . . .
sEEEvLaa.VLEiSrrEa
A0A3Q0CKB2_MESAU 682 698 USERPAT1 . . .
sEEEvLaa.VLEiSrrEa
A0A3Q0CKB2_MESAU 682 698 USERPAT1 . . .
sEEEvLaa.VLEiSrrEa

A0A3Q0CM19_MESAU 123 140 USERPAT1 . . .
tDEEqAisrVLEaSiaEn
A0A3Q0CM19_MESAU 123 140 USERPAT1 . . .
tDEEqAisrVLEaSiaEn

A0A3Q0CR36_MESAU 704 720 USERPAT1 . . .
sEEEvLaa.VLEiSrrEa
A0A3Q0CR36_MESAU 704 720 USERPAT1 . . .
sEEEvLaa.VLEiSrrEa
A0A3Q0CR36_MESAU 704 720 USERPAT1 . . .
sEEEvLaa.VLEiSrrEa

A0A3Q0E644_TARSY 113	130	USERPAT1 .	.	.
tDEEqAisrVLEaSiaEn
A0A3Q0E644_TARSY 113	130	USERPAT1 .	.	.
tDEEqAisrVLEaSiaEn

A0A3Q0FMH0_ALLSI 589	605	USERPAT1 .	.	.
sEDElLaa.VLEiSkrEa
A0A3Q0FMH0_ALLSI 589	605	USERPAT1 .	.	.
sEDElLaa.VLEiSkrEa
A0A3Q0FMH0_ALLSI 589	605	USERPAT1 .	.	.
sEDElLaa.VLEiSkrEa

A0A3Q0RDB8_AMPCI 628	644	USERPAT1 .	.	.
nDDEmLaa.VLEmSrqEa
A0A3Q0RDB8_AMPCI 628	644	USERPAT1 .	.	.
nDDEmLaa.VLEmSrqEa
A0A3Q0RDB8_AMPCI 628	644	USERPAT1 .	.	.
nDDEmLaa.VLEmSrqEa

A0A3Q1BFK3_AMPOC 687	703	USERPAT1 .	.	.
nDDEmLaa.VLEmSrqEa
A0A3Q1BFK3_AMPOC 687	703	USERPAT1 .	.	.
nDDEmLaa.VLEmSrqEa
A0A3Q1BFK3_AMPOC 687	703	USERPAT1 .	.	.
nDDEmLaa.VLEmSrqEa

A0A3Q1GR08_9TELE 688	704	USERPAT1 .	.	.
nDDEmLaa.VLEmSrqEa
A0A3Q1GR08_9TELE 688	704	USERPAT1 .	.	.
nDDEmLaa.VLEmSrqEa
A0A3Q1GR08_9TELE 688	704	USERPAT1 .	.	.
nDDEmLaa.VLEmSrqEa

A0A3Q1HVU0_ANATE 684	700	USERPAT1 .	.	.
nDDEmLaa.VLEmSrqEa
A0A3Q1HVU0_ANATE 684	700	USERPAT1 .	.	.
nDDEmLaa.VLEmSrqEa
A0A3Q1HVU0_ANATE 684	700	USERPAT1 .	.	.
nDDEmLaa.VLEmSrqEa

A0A3Q1I6Z2_ANATE 682	698	USERPAT1 .	.	.
nDDEmLaa.VLEmSrqEa
A0A3Q1I6Z2_ANATE 682	698	USERPAT1 .	.	.
nDDEmLaa.VLEmSrqEa
A0A3Q1I6Z2_ANATE 682	698	USERPAT1 .	.	.
nDDEmLaa.VLEmSrqEa

A0A3Q1LHR5_BOVIN 151	168	USERPAT1 .	.	.
tDEEqAisrVLEaSiaEn
A0A3Q1LHR5_BOVIN 151	168	USERPAT1 .	.	.
tDEEqAisrVLEaSiaEn

A0A3Q1MMX0_BOVIN 123	140	USERPAT1 .	.	.

```
tDEEqAisrVLEaSiaEn
A0A3Q1MMX0_BOVIN 123      140      USERPAT1 .        .        .
tDEEqAisrVLEaSiaEn

A0A3Q2EJH3_CYPVA 681      697      USERPAT1 .        .        .
nDEEmLaa.VLEmSrrEa
A0A3Q2EJH3_CYPVA 681      697      USERPAT1 .        .        .
nDEEmLaa.VLEmSrrEa
A0A3Q2EJH3_CYPVA 681      697      USERPAT1 .        .        .
nDEEmLaa.VLEmSrrEa

A0A3Q2GUR0_HORSE 347      363      USERPAT1 .        .        .
sEEDmLra.AVTmSleTv
A0A3Q2GUR0_HORSE 347      363      USERPAT1 .        .        .
sEEDmLra.AVTmSleTv
A0A3Q2GUR0_HORSE 347      363      USERPAT1 .        .        .
sEEDmLra.AVTmSleTv

A0A3Q2H0C3_HORSE 57       74       USERPAT1 .        .        .
tDEEqAisrVLEaSiaEn
A0A3Q2H0C3_HORSE 57       74       USERPAT1 .        .        .
tDEEqAisrVLEaSiaEn

A0A3Q2HQZ4_HORSE 97       114      USERPAT1 .        .        .
tDEEqAisrVLEaSiaEn
A0A3Q2HQZ4_HORSE 97       114      USERPAT1 .        .        .
tDEEqAisrVLEaSiaEn

A0A3Q2HYB5_HORSE 706      722      USERPAT1 .        .        .
sEEElLaa.VLEiSkrEs
A0A3Q2HYB5_HORSE 706      722      USERPAT1 .        .        .
sEEElLaa.VLEiSkrEs
A0A3Q2HYB5_HORSE 706      722      USERPAT1 .        .        .
sEEElLaa.VLEiSkrEs

A0A3Q2HYS1_HORSE 123      140      USERPAT1 .        .        .
tDEEqAisrVLEaSiaEn
A0A3Q2HYS1_HORSE 123      140      USERPAT1 .        .        .
tDEEqAisrVLEaSiaEn

A0A3Q2HZ85_HORSE 128      145      USERPAT1 .        .        .
tDEEqAisrVLEaSiaEn
A0A3Q2HZ85_HORSE 128      145      USERPAT1 .        .        .
tDEEqAisrVLEaSiaEn

A0A3Q2I9L4_HORSE 128      145      USERPAT1 .        .        .
tDEEqAisrVLEaSiaEn
A0A3Q2I9L4_HORSE 128      145      USERPAT1 .        .        .
tDEEqAisrVLEaSiaEn

A0A3Q2IK66_HORSE 662      678      USERPAT1 .        .        .
sEEElLaa.VLEiSkrEs
A0A3Q2IK66_HORSE 662      678      USERPAT1 .        .        .
sEEElLaa.VLEiSkrEs
```

A0A3Q2IK66_HORSE 662 678 USERPAT1 . . .
sEEElLaa.VLEiSkrEs

A0A3Q2L1L7_HORSE 652 668 USERPAT1 . . .
sEEElLaa.VLEiSkrEs
A0A3Q2L1L7_HORSE 652 668 USERPAT1 . . .
sEEElLaa.VLEiSkrEs
A0A3Q2L1L7_HORSE 652 668 USERPAT1 . . .
sEEElLaa.VLEiSkrEs

A0A3Q2LCD7_HORSE 57 74 USERPAT1 . . .
tDEEqAisrVLEaSiaEn
A0A3Q2LCD7_HORSE 57 74 USERPAT1 . . .
tDEEqAisrVLEaSiaEn

A0A3Q3B3M1_KRYMA 683 699 USERPAT1 . . .
nDDEmLaa.VLEmSrqEa
A0A3Q3B3M1_KRYMA 683 699 USERPAT1 . . .
nDDEmLaa.VLEmSrqEa
A0A3Q3B3M1_KRYMA 683 699 USERPAT1 . . .
nDDEmLaa.VLEmSrqEa

A0A3Q3BH33_KRYMA 670 686 USERPAT1 . . .
nDDEmLaa.VLEmSrqEa
A0A3Q3BH33_KRYMA 670 686 USERPAT1 . . .
nDDEmLaa.VLEmSrqEa
A0A3Q3BH33_KRYMA 670 686 USERPAT1 . . .
nDDEmLaa.VLEmSrqEa

A0A3Q3D0I2_HAPBU 686 702 USERPAT1 . . .
nDDEmLaa.VLEiSrqEa
A0A3Q3D0I2_HAPBU 686 702 USERPAT1 . . .
nDDEmLaa.VLEiSrqEa
A0A3Q3D0I2_HAPBU 686 702 USERPAT1 . . .
nDDEmLaa.VLEiSrqEa

A0A3Q3E4J5_9LABR 668 684 USERPAT1 . . .
nDDEmLaa.VLEmSrqEv
A0A3Q3E4J5_9LABR 668 684 USERPAT1 . . .
nDDEmLaa.VLEmSrqEv
A0A3Q3E4J5_9LABR 668 684 USERPAT1 . . .
nDDEmLaa.VLEmSrqEv

A0A3Q3M0Z7_9TELE 684 700 USERPAT1 . . .
nDDEmLaa.VLEiSrqEa
A0A3Q3M0Z7_9TELE 684 700 USERPAT1 . . .
nDDEmLaa.VLEiSrqEa
A0A3Q3M0Z7_9TELE 684 700 USERPAT1 . . .
nDDEmLaa.VLEiSrqEa

A0A3Q3VY28_MOLML 632 648 USERPAT1 . . .
nDDEmLda.VLEmSqqEa
A0A3Q3VY28_MOLML 632 648 USERPAT1 . . .
nDDEmLda.VLEmSqqEa

A0A3Q3VY28_MOLML 632 648 USERPAT1 . . .
nDDEmLda.VLEmSqqEa

A0A3Q4HLL0_NEOBR 643 659 USERPAT1 . . .
nDDEmLaa.VLEiSrqEa
A0A3Q4HLL0_NEOBR 643 659 USERPAT1 . . .
nDDEmLaa.VLEiSrqEa
A0A3Q4HLL0_NEOBR 643 659 USERPAT1 . . .
nDDEmLaa.VLEiSrqEa

A0A3Q7R581_VULVU 333 349 USERPAT1 . . .
sEEDmLqa.AVTmSleTv
A0A3Q7R581_VULVU 333 349 USERPAT1 . . .
sEEDmLqa.AVTmSleTv
A0A3Q7R581_VULVU 333 349 USERPAT1 . . .
sEEDmLqa.AVTmSleTv

A0A3Q7R7A6_VULVU 318 334 USERPAT1 . . .
sEEDmLqa.AVTmSleTv
A0A3Q7R7A6_VULVU 318 334 USERPAT1 . . .
sEEDmLqa.AVTmSleTv
A0A3Q7R7A6_VULVU 318 334 USERPAT1 . . .
sEEDmLqa.AVTmSleTv

A0A3Q7R7B1_VULVU 154 170 USERPAT1 . . .
sEEDmLqa.AVTmSleTv
A0A3Q7R7B1_VULVU 154 170 USERPAT1 . . .
sEEDmLqa.AVTmSleTv
A0A3Q7R7B1_VULVU 154 170 USERPAT1 . . .
sEEDmLqa.AVTmSleTv

A0A3Q7SHU8_VULVU 332 348 USERPAT1 . . .
sEEDmLqa.AVTmSleTv
A0A3Q7SHU8_VULVU 332 348 USERPAT1 . . .
sEEDmLqa.AVTmSleTv
A0A3Q7SHU8_VULVU 332 348 USERPAT1 . . .
sEEDmLqa.AVTmSleTv

A0A3Q7SHV3_VULVU 282 298 USERPAT1 . . .
sEEDmLqa.AVTmSleTv
A0A3Q7SHV3_VULVU 282 298 USERPAT1 . . .
sEEDmLqa.AVTmSleTv
A0A3Q7SHV3_VULVU 282 298 USERPAT1 . . .
sEEDmLqa.AVTmSleTv

A0A3Q7SQN0_VULVU 684 700 USERPAT1 . . .
sEEElLaa.VLEmSkrEa
A0A3Q7SQN0_VULVU 684 700 USERPAT1 . . .
sEEElLaa.VLEmSkrEa
A0A3Q7SQN0_VULVU 684 700 USERPAT1 . . .
sEEElLaa.VLEmSkrEa

A0A3Q7TRU5_VULVU 706 722 USERPAT1 . . .
sEEElLaa.VLEmSkrEa

A0A3Q7TRU5_VULVU 706 722 USERPAT1 . . .
sEEElLaa.VLEmSkrEa
A0A3Q7TRU5_VULVU 706 722 USERPAT1 . . .
sEEElLaa.VLEmSkrEa

A0A3Q7UH43_URSAR 197 213 USERPAT1 . . .
sEDEaLqr.ALElSlaEn

A0A3Q7V6G1_URSAR 197 213 USERPAT1 . . .
sEDEaLqr.ALElSlaEn

A0A3Q7V6K7_URSAR 705 721 USERPAT1 . . .
sEEElLaa.VLEmSkrEs
A0A3Q7V6K7_URSAR 705 721 USERPAT1 . . .
sEEElLaa.VLEmSkrEs
A0A3Q7V6K7_URSAR 705 721 USERPAT1 . . .
sEEElLaa.VLEmSkrEs

A0A3Q7VYY1_URSAR 683 699 USERPAT1 . . .
sEEElLaa.VLEmSkrEs
A0A3Q7VYY1_URSAR 683 699 USERPAT1 . . .
sEEElLaa.VLEmSkrEs
A0A3Q7VYY1_URSAR 683 699 USERPAT1 . . .
sEEElLaa.VLEmSkrEs

A0A3Q7WBN1_URSAR 705 721 USERPAT1 . . .
sEEElLaa.VLEmSkrEs
A0A3Q7WBN1_URSAR 705 721 USERPAT1 . . .
sEEElLaa.VLEmSkrEs
A0A3Q7WBN1_URSAR 705 721 USERPAT1 . . .
sEEElLaa.VLEmSkrEs

A0A3Q7XA14_URSAR 326 342 USERPAT1 . . .
sEEDmLqa.AVTlSleTv
A0A3Q7XA14_URSAR 326 342 USERPAT1 . . .
sEEDmLqa.AVTlSleTv
A0A3Q7XA14_URSAR 326 342 USERPAT1 . . .
sEEDmLqa.AVTlSleTv

A0A3Q7XA19_URSAR 148 164 USERPAT1 . . .
sEEDmLqa.AVTlSleTv
A0A3Q7XA19_URSAR 148 164 USERPAT1 . . .
sEEDmLqa.AVTlSleTv
A0A3Q7XA19_URSAR 148 164 USERPAT1 . . .
sEEDmLqa.AVTlSleTv

A0A3Q7XVY1_URSAR 276 292 USERPAT1 . . .
sEEDmLqa.AVTlSleTv
A0A3Q7XVY1_URSAR 276 292 USERPAT1 . . .
sEEDmLqa.AVTlSleTv
A0A3Q7XVY1_URSAR 276 292 USERPAT1 . . .
sEEDmLqa.AVTlSleTv

A0A3Q7Y3E5_URSAR 327 343 USERPAT1 . . .

sEEDmLqa.AVTlSleTv
A0A3Q7Y3E5_URSAR 327 343 USERPAT1 . . .
sEEDmLqa.AVTlSleTv
A0A3Q7Y3E5_URSAR 327 343 USERPAT1 . . .
sEEDmLqa.AVTlSleTv

A0A3R7PRM8_PENVA 1 18 USERPAT1 . . .
mDDDdLlslALHmSaqEa
A0A3R7PRM8_PENVA 1 18 USERPAT1 . . .
mDDDdLlslALHmSaqEa

A0A401P2Z5_SCYTO 227 243 USERPAT1 . . .
dDEEsLqr.ALTlSrqHm
A0A401P2Z5_SCYTO 227 243 USERPAT1 . . .
dDEEsLqr.ALTlSrqHm

A0A401RIH8_CHIPU 643 659 USERPAT1 . . .
sEDElLaa.VLEiSkrEt
A0A401RIH8_CHIPU 643 659 USERPAT1 . . .
sEDElLaa.VLEiSkrEt
A0A401RIH8_CHIPU 643 659 USERPAT1 . . .
sEDElLaa.VLEiSkrEt
A0A401RIH8_CHIPU 643 659 USERPAT1 . . .
sEDElLaa.VLEiSkrEt

A0A401SMB2_CHIPU 50 66 USERPAT1 . . .
eEDEnLqr.ALEqSrkEs
A0A401SMB2_CHIPU 50 66 USERPAT1 . . .
eEDEnLqr.ALEqSrkEs

A0A401SSW7_CHIPU 318 334 USERPAT1 . . .
eDDEnLqr.ALTlSrqHm
A0A401SSW7_CHIPU 318 334 USERPAT1 . . .
eDDEnLqr.ALTlSrqHm

A0A402F317_9SAUR 711 727 USERPAT1 . . .
nEDElFaa.VLEiSkrEa
A0A402F317_9SAUR 711 727 USERPAT1 . . .
nEDElFaa.VLEiSkrEa

A0A452CL78_BALAS 198 214 USERPAT1 . . .
sEDEaLqr.ALElSlaEa

A0A452DJ84_BOVIN 706 722 USERPAT1 . . .
sEEElLaa.VLEiSkrEa
A0A452DJ84_BOVIN 706 722 USERPAT1 . . .
sEEElLaa.VLEiSkrEa
A0A452DJ84_BOVIN 706 722 USERPAT1 . . .
sEEElLaa.VLEiSkrEa

A0A452DUW2_CAPHI 706 722 USERPAT1 . . .
sEEElLaa.VLEiSkrEa
A0A452DUW2_CAPHI 706 722 USERPAT1 . . .
sEEElLaa.VLEiSkrEa

A0A452DUW2_CAPHI 706 722 USERPAT1 . . .
sEEElLaa.VLEiSkrEa

A0A452ELE0_CAPHI 334 350 USERPAT1 . . .
sEEDmLqa.AVTmSleTv
A0A452ELE0_CAPHI 334 350 USERPAT1 . . .
sEEDmLqa.AVTmSleTv
A0A452ELE0_CAPHI 334 350 USERPAT1 . . .
sEEDmLqa.AVTmSleTv

A0A452ERN2_CAPHI 151 168 USERPAT1 . . .
tDEEqAisrVLEaSiaEn
A0A452ERN2_CAPHI 151 168 USERPAT1 . . .
tDEEqAisrVLEaSiaEn

A0A452ERQ3_CAPHI 118 135 USERPAT1 . . .
tDEEqAisrVLEaSiaEn
A0A452ERQ3_CAPHI 118 135 USERPAT1 . . .
tDEEqAisrVLEaSiaEn

A0A452ERR7_CAPHI 146 163 USERPAT1 . . .
tDEEqAisrVLEaSiaEn
A0A452ERR7_CAPHI 146 163 USERPAT1 . . .
tDEEqAisrVLEaSiaEn

A0A452ERT6_CAPHI 123 140 USERPAT1 . . .
tDEEqAisrVLEaSiaEn
A0A452ERT6_CAPHI 123 140 USERPAT1 . . .
tDEEqAisrVLEaSiaEn

A0A452ERW3_CAPHI 151 168 USERPAT1 . . .
tDEEqAisrVLEaSiaEn
A0A452ERW3_CAPHI 151 168 USERPAT1 . . .
tDEEqAisrVLEaSiaEn

A0A452H1J7_9SAUR 715 731 USERPAT1 . . .
sEDElLaa.VLEiSkrEa
A0A452H1J7_9SAUR 715 731 USERPAT1 . . .
sEDElLaa.VLEiSkrEa
A0A452H1J7_9SAUR 715 731 USERPAT1 . . .
sEDElLaa.VLEiSkrEa

A0A452H1R4_9SAUR 715 731 USERPAT1 . . .
sEDElLaa.VLEiSkrEa
A0A452H1R4_9SAUR 715 731 USERPAT1 . . .
sEDElLaa.VLEiSkrEa
A0A452H1R4_9SAUR 715 731 USERPAT1 . . .
sEDElLaa.VLEiSkrEa

A0A452H270_9SAUR 88 104 USERPAT1 . . .
sEDElLaa.VLEiSkrEa
A0A452H270_9SAUR 88 104 USERPAT1 . . .
sEDElLaa.VLEiSkrEa
A0A452H270_9SAUR 88 104 USERPAT1 . . .

```
sEDElLaa.VLEiSkrEa

A0A452Q8F3_URSAM 95       111      USERPAT1 .        .        .
sEEElLaa.VLEmSkrEs
A0A452Q8F3_URSAM 95       111      USERPAT1 .        .        .
sEEElLaa.VLEmSkrEs
A0A452Q8F3_URSAM 95       111      USERPAT1 .        .        .
sEEElLaa.VLEmSkrEs

A0A452QRA9_URSAM 197      213      USERPAT1 .        .        .
sEDEaLqr.ALElSlaEn

A0A452SVJ4_URSAM 275      291      USERPAT1 .        .        .
sEEDmLqa.AVTlSleTv
A0A452SVJ4_URSAM 275      291      USERPAT1 .        .        .
sEEDmLqa.AVTlSleTv
A0A452SVJ4_URSAM 275      291      USERPAT1 .        .        .
sEEDmLqa.AVTlSleTv

A0A452SVY9_URSAM 263      279      USERPAT1 .        .        .
sEEDmLqa.AVTlSleTv
A0A452SVY9_URSAM 263      279      USERPAT1 .        .        .
sEEDmLqa.AVTlSleTv
A0A452SVY9_URSAM 263      279      USERPAT1 .        .        .
sEEDmLqa.AVTlSleTv

A0A452SW03_URSAM 325      341      USERPAT1 .        .        .
sEEDmLqa.AVTlSleTv
A0A452SW03_URSAM 325      341      USERPAT1 .        .        .
sEEDmLqa.AVTlSleTv
A0A452SW03_URSAM 325      341      USERPAT1 .        .        .
sEEDmLqa.AVTlSleTv

A0A452SW18_URSAM 332      348      USERPAT1 .        .        .
sEEDmLqa.AVTlSleTv
A0A452SW18_URSAM 332      348      USERPAT1 .        .        .
sEEDmLqa.AVTlSleTv
A0A452SW18_URSAM 332      348      USERPAT1 .        .        .
sEEDmLqa.AVTlSleTv

A0A452SW49_URSAM 303      319      USERPAT1 .        .        .
sEEDmLqa.AVTlSleTv
A0A452SW49_URSAM 303      319      USERPAT1 .        .        .
sEEDmLqa.AVTlSleTv
A0A452SW49_URSAM 303      319      USERPAT1 .        .        .
sEEDmLqa.AVTlSleTv

A0A455ANT6_PHYMC 706      722      USERPAT1 .        .        .
sEEElLaa.VLEiSkrEa
A0A455ANT6_PHYMC 706      722      USERPAT1 .        .        .
sEEElLaa.VLEiSkrEa
A0A455ANT6_PHYMC 706      722      USERPAT1 .        .        .
sEEElLaa.VLEiSkrEa
```

A0A455B0F9_PHYMC 706 722 USERPAT1 . . .
sEEElLaa.VLEiSkrEa
A0A455B0F9_PHYMC 706 722 USERPAT1 . . .
sEEElLaa.VLEiSkrEa
A0A455B0F9_PHYMC 706 722 USERPAT1 . . .
sEEElLaa.VLEiSkrEa

A0A455BMJ7_PHYMC 151 168 USERPAT1 . . .
tDEEqAisrVLEaSiaEn
A0A455BMJ7_PHYMC 151 168 USERPAT1 . . .
tDEEqAisrVLEaSiaEn

A0A455BQW5_PHYMC 151 168 USERPAT1 . . .
tDEEqAisrVLEaSiaEn
A0A455BQW5_PHYMC 151 168 USERPAT1 . . .
tDEEqAisrVLEaSiaEn

A0A455BT30_PHYMC 279 295 USERPAT1 . . .
sEEDmLqa.AVTmSleTv
A0A455BT30_PHYMC 279 295 USERPAT1 . . .
sEEDmLqa.AVTmSleTv
A0A455BT30_PHYMC 279 295 USERPAT1 . . .
sEEDmLqa.AVTmSleTv

A0A455BTS4_PHYMC 272 288 USERPAT1 . . .
sEEDmLqa.AVTmSleTv
A0A455BTS4_PHYMC 272 288 USERPAT1 . . .
sEEDmLqa.AVTmSleTv
A0A455BTS4_PHYMC 272 288 USERPAT1 . . .
sEEDmLqa.AVTmSleTv

A0A455C2P2_PHYMC 151 168 USERPAT1 . . .
tDEEqAisrVLEaSiaEn
A0A455C2P2_PHYMC 151 168 USERPAT1 . . .
tDEEqAisrVLEaSiaEn

A0A455C853_PHYMC 273 289 USERPAT1 . . .
sEEDmLqa.AVTmSleTv
A0A455C853_PHYMC 273 289 USERPAT1 . . .
sEEDmLqa.AVTmSleTv
A0A455C853_PHYMC 273 289 USERPAT1 . . .
sEEDmLqa.AVTmSleTv

A0A484BT32_DRONA 563 579 USERPAT1 . . .
eDDEqLqr.VLHeSlmGa
A0A484BT32_DRONA 563 579 USERPAT1 . . .
eDDEqLqr.VLHeSlmGa
A0A484BT32_DRONA 563 579 USERPAT1 . . .
eDDEqLqr.VLHeSlmGa
A0A484BT32_DRONA 563 579 USERPAT1 . . .
eDDEqLqr.VLHeSlmGa

A0A498LFL3_LABRO 630 646 USERPAT1 . . .
nDEEmLaa.VLEmSrhDt

A0A498LFL3_LABRO 630 646 USERPAT1 . . .
nDEEmLaa.VLEmSrhDt

A0A498M7M2_LABRO 122 139 USERPAT1 . . .
tDEEqAisrVLEaSiaEn
A0A498M7M2_LABRO 122 139 USERPAT1 . . .
tDEEqAisrVLEaSiaEn

A0A4D9DMU2_9SAUR 205 221 USERPAT1 . . .
sEEEaLqr.ALEmSlaEs
A0A4D9DMU2_9SAUR 205 221 USERPAT1 . . .
sEEEaLqr.ALEmSlaEs

A0A4D9E540_9SAUR 715 731 USERPAT1 . . .
sEDElLaa.VLEiSkrEa
A0A4D9E540_9SAUR 715 731 USERPAT1 . . .
sEDElLaa.VLEiSkrEa
A0A4D9E540_9SAUR 715 731 USERPAT1 . . .
sEDElLaa.VLEiSkrEa

A2EEG1_TRIVA 216 232 USERPAT1 . . .
dEDEeFrr.VLEeSrhEt
A2EEG1_TRIVA 216 232 USERPAT1 . . .
dEDEeFrr.VLEeSrhEt
A2EEG1_TRIVA 216 232 USERPAT1 . . .
dEDEeFrr.VLEeSrhEt
A2EEG1_TRIVA 216 232 USERPAT1 . . .
dEDEeFrr.VLEeSrhEt

A3GG13_PICST 656 673 USERPAT1 . . .
dEEDeVmrrVLElSlhEh
A3GG13_PICST 656 673 USERPAT1 . . .
dEEDeVmrrVLElSlhEh
A3GG13_PICST 656 673 USERPAT1 . . .
dEEDeVmrrVLElSlhEh

ATX3L_HUMAN 224 240 USERPAT1 . . .
qDEEdFqr.ALElSrqEt
ATX3L_HUMAN 224 240 USERPAT1 . . .
qDEEdFqr.ALElSrqEt

ATX3_HUMAN 335 351 USERPAT1 . . .
sEEDmLqa.AVTmSleTv
ATX3_HUMAN 335 351 USERPAT1 . . .
sEEDmLqa.AVTmSleTv
ATX3_HUMAN 335 351 USERPAT1 . . .
sEEDmLqa.AVTmSleTv

ATX3_MOUSE 329 345 USERPAT1 . . .
sEEDmLra.AVTmSleTa
ATX3_MOUSE 329 345 USERPAT1 . . .
sEEDmLra.AVTmSleTa
ATX3_MOUSE 329 345 USERPAT1 . . .
sEEDmLra.AVTmSleTa

B4H729_DROPE 504 520 USERPAT1 . . .
eDDEqLqr.VLHeSllGa
B4H729_DROPE 504 520 USERPAT1 . . .
eDDEqLqr.VLHeSllGa
B4H729_DROPE 504 520 USERPAT1 . . .
eDDEqLqr.VLHeSllGa
B4H729_DROPE 504 520 USERPAT1 . . .
eDDEqLqr.VLHeSllGa

B4JVM8_DROGR 572 588 USERPAT1 . . .
eDDEqLqr.VLHeSllGa
B4JVM8_DROGR 572 588 USERPAT1 . . .
eDDEqLqr.VLHeSllGa
B4JVM8_DROGR 572 588 USERPAT1 . . .
eDDEqLqr.VLHeSllGa
B4JVM8_DROGR 572 588 USERPAT1 . . .
eDDEqLqr.VLHeSllGa

B4KU71_DROMO 563 579 USERPAT1 . . .
eDDEqLqr.VLHeSlmGa
B4KU71_DROMO 563 579 USERPAT1 . . .
eDDEqLqr.VLHeSlmGa
B4KU71_DROMO 563 579 USERPAT1 . . .
eDDEqLqr.VLHeSlmGa
B4KU71_DROMO 563 579 USERPAT1 . . .
eDDEqLqr.VLHeSlmGa

B4MCN8_DROVI 564 580 USERPAT1 . . .
eDDEqLqr.VLHeSllGa
B4MCN8_DROVI 564 580 USERPAT1 . . .
eDDEqLqr.VLHeSllGa
B4MCN8_DROVI 564 580 USERPAT1 . . .
eDDEqLqr.VLHeSllGa
B4MCN8_DROVI 564 580 USERPAT1 . . .
eDDEqLqr.VLHeSllGa

C9JQV6_HUMAN 265 281 USERPAT1 . . .
sEEDmLqa.AVTmSleTv
C9JQV6_HUMAN 265 281 USERPAT1 . . .
sEEDmLqa.AVTmSleTv
C9JQV6_HUMAN 265 281 USERPAT1 . . .
sEEDmLqa.AVTmSleTv

C9SHD9_VERA1 1166 1182 USERPAT1 . . .
dDDEdFkr.ALEaSkiPs
C9SHD9_VERA1 1166 1182 USERPAT1 . . .
dDDEdFkr.ALEaSkiPs
C9SHD9_VERA1 1166 1182 USERPAT1 . . .
dDDEdFkr.ALEaSkiPs

D2V513_NAEGR 146 162 USERPAT1 . . .
nEDEeLqr.VLElSkqEh
D2V513_NAEGR 146 162 USERPAT1 . . .

nEDEeLqr.VLElSkqEh

D4ABE5_RAT 704 720 USERPAT1 . . .
sEEEvLaa.VLEiSrrEa
D4ABE5_RAT 704 720 USERPAT1 . . .
sEEEvLaa.VLEiSrrEa
D4ABE5_RAT 704 720 USERPAT1 . . .
sEEEvLaa.VLEiSrrEa

D4ACD3_RAT 124 141 USERPAT1 . . .
tDEEqAisrVLEaSiaEn
D4ACD3_RAT 124 141 USERPAT1 . . .
tDEEqAisrVLEaSiaEn

DAR6_ARATH 244 260 USERPAT1 . . .
dEDEqLak.AVEeSlkGk
DAR6_ARATH 244 260 USERPAT1 . . .
dEDEqLak.AVEeSlkGk

E0CY18_MOUSE 197 213 USERPAT1 . . .
sEDEaLqr.ALElSlaEa

E1ZSZ0_CHLVA 160 176 USERPAT1 . . .
dEDEeLqr.ALEmSmaGg

E2QUQ9_CANLF 333 349 USERPAT1 . . .
sEEDmLqa.AVTmSleTv
E2QUQ9_CANLF 333 349 USERPAT1 . . .
sEEDmLqa.AVTmSleTv
E2QUQ9_CANLF 333 349 USERPAT1 . . .
sEEDmLqa.AVTmSleTv

E2QW53_CANLF 123 140 USERPAT1 . . .
tDEEqAisrVLEaSiaEn
E2QW53_CANLF 123 140 USERPAT1 . . .
tDEEqAisrVLEaSiaEn

E2RHS7_CANLF 326 342 USERPAT1 . . .
sEEDmLqa.AVTmSleTv
E2RHS7_CANLF 326 342 USERPAT1 . . .
sEEDmLqa.AVTmSleTv
E2RHS7_CANLF 326 342 USERPAT1 . . .
sEEDmLqa.AVTmSleTv

E2RK08_CANLF 666 682 USERPAT1 . . .
sEEElLaa.VLEmSkrEa
E2RK08_CANLF 666 682 USERPAT1 . . .
sEEElLaa.VLEmSkrEa
E2RK08_CANLF 666 682 USERPAT1 . . .
sEEElLaa.VLEmSkrEa

E9PFU9_HUMAN 197 213 USERPAT1 . . .
sEDEaLqr.ALEmSlaEt

F1MP13_BOVIN 151 168 USERPAT1 . . .
tDEEqAisrVLEaSiaEn
F1MP13_BOVIN 151 168 USERPAT1 . . .
tDEEqAisrVLEaSiaEn

F2TYZ4_SALR5 201 217 USERPAT1 . . .
aEDEeLqr.VLElSaeEh
F2TYZ4_SALR5 201 217 USERPAT1 . . .
aEDEeLqr.VLElSaeEh

F5H211_HUMAN 344 360 USERPAT1 . . .
sEEDmLqa.AVTmSleTv
F5H211_HUMAN 344 360 USERPAT1 . . .
sEEDmLqa.AVTmSleTv
F5H211_HUMAN 344 360 USERPAT1 . . .
sEEDmLqa.AVTmSleTv

F6SMT5_MONDO 711 727 USERPAT1 . . .
sEDElLaa.VLEiSkrEa
F6SMT5_MONDO 711 727 USERPAT1 . . .
sEDElLaa.VLEiSkrEa
F6SMT5_MONDO 711 727 USERPAT1 . . .
sEDElLaa.VLEiSkrEa

F6SNZ3_MONDO 123 140 USERPAT1 . . .
tDEEqAisrVLEaSiaEn
F6SNZ3_MONDO 123 140 USERPAT1 . . .
tDEEqAisrVLEaSiaEn

F6SVL6_CALJA 347 363 USERPAT1 . . .
sEEDmLqa.AVTmSleTv
F6SVL6_CALJA 347 363 USERPAT1 . . .
sEEDmLqa.AVTmSleTv
F6SVL6_CALJA 347 363 USERPAT1 . . .
sEEDmLqa.AVTmSleTv

F6SVY6_CALJA 285 301 USERPAT1 . . .
sEEDmLqa.AVTmSleTv
F6SVY6_CALJA 285 301 USERPAT1 . . .
sEEDmLqa.AVTmSleTv
F6SVY6_CALJA 285 301 USERPAT1 . . .
sEEDmLqa.AVTmSleTv

F6TSS4_HORSE 333 349 USERPAT1 . . .
sEEDmLra.AVTmSleTv
F6TSS4_HORSE 333 349 USERPAT1 . . .
sEEDmLra.AVTmSleTv
F6TSS4_HORSE 333 349 USERPAT1 . . .
sEEDmLra.AVTmSleTv

F6VWD3_MACMU 706 722 USERPAT1 . . .
sEEElLaa.VLEiSkrDa
F6VWD3_MACMU 706 722 USERPAT1 . . .
sEEElLaa.VLEiSkrDa

F6Y2G0_MACMU 123 140 USERPAT1 . . .
tDEEqAisrVLEaSiaEn
F6Y2G0_MACMU 123 140 USERPAT1 . . .
tDEEqAisrVLEaSiaEn

F6Y2I3_MACMU 123 140 USERPAT1 . . .
tDEEqAisrVLEaSiaEn
F6Y2I3_MACMU 123 140 USERPAT1 . . .
tDEEqAisrVLEaSiaEn

F6Y2J9_MACMU 123 140 USERPAT1 . . .
tDEEqAisrVLEaSiaEn
F6Y2J9_MACMU 123 140 USERPAT1 . . .
tDEEqAisrVLEaSiaEn

F6YF39_XENTR 208 224 USERPAT1 . . .
tEEEaLqr.ALEmSlaEs

F6ZG63_HORSE 684 700 USERPAT1 . . .
sEEElLaa.VLEiSkrEs
F6ZG63_HORSE 684 700 USERPAT1 . . .
sEEElLaa.VLEiSkrEs
F6ZG63_HORSE 684 700 USERPAT1 . . .
sEEElLaa.VLEiSkrEs

F6ZIZ7_HORSE 57 74 USERPAT1 . . .
tDEEqAisrVLEaSiaEn
F6ZIZ7_HORSE 57 74 USERPAT1 . . .
tDEEqAisrVLEaSiaEn

F7AII7_ORNAN 124 141 USERPAT1 . . .
tDEEqAisrVLEaSiaEn
F7AII7_ORNAN 124 141 USERPAT1 . . .
tDEEqAisrVLEaSiaEn

F7AIJ4_ORNAN 124 141 USERPAT1 . . .
tDEEqAisrVLEaSiaEn
F7AIJ4_ORNAN 124 141 USERPAT1 . . .
tDEEqAisrVLEaSiaEn

F7ARV7_CALJA 123 140 USERPAT1 . . .
tDEEqAisrVLEaSiaEn
F7ARV7_CALJA 123 140 USERPAT1 . . .
tDEEqAisrVLEaSiaEn

F7DC76_XENTR 711 727 USERPAT1 . . .
nDDElVaa.VLEmSkkEt
F7DC76_XENTR 711 727 USERPAT1 . . .
nDDElVaa.VLEmSkkEt

F7DY46_CALJA 123 140 USERPAT1 . . .
tDEEqAisrVLEaSiaEn
F7DY46_CALJA 123 140 USERPAT1 . . .

tDEEqAisrVLEaSiaEn

F7ES35_CALJA 706 722 USERPAT1 . . .
sEEElLaa.VLEiSkrEa
F7ES35_CALJA 706 722 USERPAT1 . . .
sEEElLaa.VLEiSkrEa
F7ES35_CALJA 706 722 USERPAT1 . . .
sEEElLaa.VLEiSkrEa

F7F5L2_CALJA 123 140 USERPAT1 . . .
tDEEqAisrVLEaSiaEn
F7F5L2_CALJA 123 140 USERPAT1 . . .
tDEEqAisrVLEaSiaEn

F7F5M5_CALJA 612 628 USERPAT1 . . .
sEEElLaa.VLEiSkrEa
F7F5M5_CALJA 612 628 USERPAT1 . . .
sEEElLaa.VLEiSkrEa
F7F5M5_CALJA 612 628 USERPAT1 . . .
sEEElLaa.VLEiSkrEa

F7F5Q1_CALJA 123 140 USERPAT1 . . .
tDEEqAisrVLEaSiaEn
F7F5Q1_CALJA 123 140 USERPAT1 . . .
tDEEqAisrVLEaSiaEn

G1KAT4_ANOCA 697 713 USERPAT1 . . .
nEDElLaa.VLEmSkkEt
G1KAT4_ANOCA 697 713 USERPAT1 . . .
nEDElLaa.VLEmSkkEt
G1KAT4_ANOCA 697 713 USERPAT1 . . .
nEDElLaa.VLEmSkkEt

G1L8C8_AILME 706 722 USERPAT1 . . .
sEEElLaa.VLEmSkrEs
G1L8C8_AILME 706 722 USERPAT1 . . .
sEEElLaa.VLEmSkrEs
G1L8C8_AILME 706 722 USERPAT1 . . .
sEEElLaa.VLEmSkrEs

G1M8G9_AILME 320 336 USERPAT1 . . .
sEEDmLqa.AVTmSlaTv
G1M8G9_AILME 320 336 USERPAT1 . . .
sEEDmLqa.AVTmSlaTv
G1M8G9_AILME 320 336 USERPAT1 . . .
sEEDmLqa.AVTmSlaTv

G1NG28_MELGA 715 731 USERPAT1 . . .
sEDElLaa.VLEiSkrEa
G1NG28_MELGA 715 731 USERPAT1 . . .
sEDElLaa.VLEiSkrEa
G1NG28_MELGA 715 731 USERPAT1 . . .
sEDElLaa.VLEiSkrEa

G1PHP0_MYOLU 108 125 USERPAT1 . . .
tDEEqAisrVLEaSiaEn
G1PHP0_MYOLU 108 125 USERPAT1 . . .
tDEEqAisrVLEaSiaEn

G1QNB5_NOMLE 116 133 USERPAT1 . . .
tDEEqAisrVLEaSiaEn
G1QNB5_NOMLE 116 133 USERPAT1 . . .
tDEEqAisrVLEaSiaEn

G1RBP9_NOMLE 706 722 USERPAT1 . . .
sEEElLaa.VLEiSkrDa
G1RBP9_NOMLE 706 722 USERPAT1 . . .
sEEElLaa.VLEiSkrDa

G1SMF4_RABIT 151 168 USERPAT1 . . .
tDEEqAisrVLEaSiaEn
G1SMF4_RABIT 151 168 USERPAT1 . . .
tDEEqAisrVLEaSiaEn

G1SPH1_RABIT 322 338 USERPAT1 . . .
sEEDmLqa.AVTmSleTv
G1SPH1_RABIT 322 338 USERPAT1 . . .
sEEDmLqa.AVTmSleTv
G1SPH1_RABIT 322 338 USERPAT1 . . .
sEEDmLqa.AVTmSleTv

G1T3H8_RABIT 706 722 USERPAT1 . . .
nEEElIaa.VLEiSkrEt
G1T3H8_RABIT 706 722 USERPAT1 . . .
nEEElIaa.VLEiSkrEt

G2WTE6_VERDV 1167 1183 USERPAT1 . . .
dDDEdFkr.ALEaSkiPs
G2WTE6_VERDV 1167 1183 USERPAT1 . . .
dDDEdFkr.ALEaSkiPs
G2WTE6_VERDV 1167 1183 USERPAT1 . . .
dDDEdFkr.ALEaSkiPs
G2WTE6_VERDV 1167 1183 USERPAT1 . . .
dDDEdFkr.ALEaSkiPs

G3HWH3_CRIGR 704 720 USERPAT1 . . .
sEEEvLaa.VLEiSrrEa
G3HWH3_CRIGR 704 720 USERPAT1 . . .
sEEEvLaa.VLEiSrrEa
G3HWH3_CRIGR 704 720 USERPAT1 . . .
sEEEvLaa.VLEiSrrEa

G3I1H9_CRIGR 281 297 USERPAT1 . . .
rEEDmLra.AVNmSleTa
G3I1H9_CRIGR 281 297 USERPAT1 . . .
rEEDmLra.AVNmSleTa
G3I1H9_CRIGR 281 297 USERPAT1 . . .
rEEDmLra.AVNmSleTa

G3NDF4_GASAC 696 712 USERPAT1 . . .
nDDEmLaa.VLEmSrqEa
G3NDF4_GASAC 696 712 USERPAT1 . . .
nDDEmLaa.VLEmSrqEa
G3NDF4_GASAC 696 712 USERPAT1 . . .
nDDEmLaa.VLEmSrqEa

G3Q9R1_GASAC 122 139 USERPAT1 . . .
tDEEqAisrVLEaSiaEn
G3Q9R1_GASAC 122 139 USERPAT1 . . .
tDEEqAisrVLEaSiaEn

G3QGR2_GORGO 123 140 USERPAT1 . . .
tDEEqAisrVLEaSiaEn
G3QGR2_GORGO 123 140 USERPAT1 . . .
tDEEqAisrVLEaSiaEn

G3RE51_GORGO 276 292 USERPAT1 . . .
sEEDmLqa.AVTmSleTv
G3RE51_GORGO 276 292 USERPAT1 . . .
sEEDmLqa.AVTmSleTv
G3RE51_GORGO 276 292 USERPAT1 . . .
sEEDmLqa.AVTmSleTv

G3REK7_GORGO 610 626 USERPAT1 . . .
sEEElLaa.VLEiSkrDa
G3REK7_GORGO 610 626 USERPAT1 . . .
sEEElLaa.VLEiSkrDa

G3S3D3_GORGO 704 720 USERPAT1 . . .
sEEElLaa.VLEiSkrDa
G3S3D3_GORGO 704 720 USERPAT1 . . .
sEEElLaa.VLEiSkrDa

G3SA57_GORGO 261 277 USERPAT1 . . .
sEEDmLqa.AVTmSleTv
G3SA57_GORGO 261 277 USERPAT1 . . .
sEEDmLqa.AVTmSleTv
G3SA57_GORGO 261 277 USERPAT1 . . .
sEEDmLqa.AVTmSleTv

G3TA40_LOXAF 35 52 USERPAT1 . . .
tDEEqAisrVLEaSiaEn
G3TA40_LOXAF 35 52 USERPAT1 . . .
tDEEqAisrVLEaSiaEn

G3TES3_LOXAF 707 723 USERPAT1 . . .
sEEElVaa.VLEiSkrEt
G3TES3_LOXAF 707 723 USERPAT1 . . .
sEEElVaa.VLEiSkrEt

G3VV53_SARHA 136 153 USERPAT1 . . .
tDEEqAisrVLEaSiaEn

```
G3VV53_SARHA     136      153      USERPAT1 .        .        .
tDEEqAisrVLEaSiaEn

G3VV54_SARHA     123      140      USERPAT1 .        .        .
tDEEqAisrVLEaSiaEn
G3VV54_SARHA     123      140      USERPAT1 .        .        .
tDEEqAisrVLEaSiaEn

G3WTH5_SARHA     711      727      USERPAT1 .        .        .
sEDElLaa.VLEiSkkEa
G3WTH5_SARHA     711      727      USERPAT1 .        .        .
sEDElLaa.VLEiSkkEa
G3WTH5_SARHA     711      727      USERPAT1 .        .        .
sEDElLaa.VLEiSkkEa

G3WTH6_SARHA     710      726      USERPAT1 .        .        .
sEDElLaa.VLEiSkkEa
G3WTH6_SARHA     710      726      USERPAT1 .        .        .
sEDElLaa.VLEiSkkEa
G3WTH6_SARHA     710      726      USERPAT1 .        .        .
sEDElLaa.VLEiSkkEa

G5AZI7_HETGA     638      654      USERPAT1 .        .        .
sEEElLaa.VLEiSkrEt
G5AZI7_HETGA     638      654      USERPAT1 .        .        .
sEEElLaa.VLEiSkrEt
G5AZI7_HETGA     638      654      USERPAT1 .        .        .
sEEElLaa.VLEiSkrEt

G5AZL7_HETGA     347      363      USERPAT1 .        .        .
sEEDmLqa.AVTmSleTv
G5AZL7_HETGA     347      363      USERPAT1 .        .        .
sEEDmLqa.AVTmSleTv
G5AZL7_HETGA     347      363      USERPAT1 .        .        .
sEEDmLqa.AVTmSleTv

G8Y9V0_PICSO     279      295      USERPAT1 .        .        .
dEDEqLrr.ALElSlqDs
G8Y9V0_PICSO     279      295      USERPAT1 .        .        .
dEDEqLrr.ALElSlqDs

H0UXL2_CAVPO     123      140      USERPAT1 .        .        .
tDEEqAisrVLEaSiaEn
H0UXL2_CAVPO     123      140      USERPAT1 .        .        .
tDEEqAisrVLEaSiaEn

H0VHY0_CAVPO     325      341      USERPAT1 .        .        .
sEEDmLqa.AVTmSleTv
H0VHY0_CAVPO     325      341      USERPAT1 .        .        .
sEEDmLqa.AVTmSleTv
H0VHY0_CAVPO     325      341      USERPAT1 .        .        .
sEEDmLqa.AVTmSleTv

H0VSL2_CAVPO     706      722      USERPAT1 .        .        .
```

nEEElLaa.VLEiSkrEa
H0VSL2_CAVPO 706 722 USERPAT1 . . .
nEEElLaa.VLEiSkrEa
H0VSL2_CAVPO 706 722 USERPAT1 . . .
nEEElLaa.VLEiSkrEa

H0WPC2_OTOGA 711 727 USERPAT1 . . .
sEEElLta.VLEiSkrEt
H0WPC2_OTOGA 711 727 USERPAT1 . . .
sEEElLta.VLEiSkrEt

H0X9P3_OTOGA 126 143 USERPAT1 . . .
tDEEqAisrVLEaSiaEn
H0X9P3_OTOGA 126 143 USERPAT1 . . .
tDEEqAisrVLEaSiaEn

H0Z4Z5_TAEGU 714 730 USERPAT1 . . .
sEDElLaa.VLEiSkrEa
H0Z4Z5_TAEGU 714 730 USERPAT1 . . .
sEDElLaa.VLEiSkrEa
H0Z4Z5_TAEGU 714 730 USERPAT1 . . .
sEDElLaa.VLEiSkrEa

H0ZTK0_TAEGU 123 140 USERPAT1 . . .
tDEEqAisrVLEaSiaEn
H0ZTK0_TAEGU 123 140 USERPAT1 . . .
tDEEqAisrVLEaSiaEn

H2MJB1_ORYLA 653 669 USERPAT1 . . .
nDDEmLaa.VLEmSrqEa
H2MJB1_ORYLA 653 669 USERPAT1 . . .
nDDEmLaa.VLEmSrqEa
H2MJB1_ORYLA 653 669 USERPAT1 . . .
nDDEmLaa.VLEmSrqEa

H2NM22_PONAB 305 321 USERPAT1 . . .
sEEDmLqa.AVTmSleTv
H2NM22_PONAB 305 321 USERPAT1 . . .
sEEDmLqa.AVTmSleTv
H2NM22_PONAB 305 321 USERPAT1 . . .
sEEDmLqa.AVTmSleTv

H2P2S3_PONAB 108 125 USERPAT1 . . .
tDEEqAisrVLEaSiaEn
H2P2S3_PONAB 108 125 USERPAT1 . . .
tDEEqAisrVLEaSiaEn

H2P2S4_PONAB 108 125 USERPAT1 . . .
tDEEqAisrVLEaSiaEn
H2P2S4_PONAB 108 125 USERPAT1 . . .
tDEEqAisrVLEaSiaEn

H2P8L1_PONAB 620 636 USERPAT1 . . .
sEEElLaa.VLEiSkrDa

H2P8L1_PONAB 620 636 USERPAT1 . . .
sEEElLaa.VLEiSkrDa

H2QJF0_PANTR 704 720 USERPAT1 . . .
sEEElLaa.VLEiSkrDa
H2QJF0_PANTR 704 720 USERPAT1 . . .
sEEElLaa.VLEiSkrDa

H2QKV1_PANTR 116 133 USERPAT1 . . .
tDEEqAisrVLEaSiaEn
H2QKV1_PANTR 116 133 USERPAT1 . . .
tDEEqAisrVLEaSiaEn

H2R2I4_PANTR 348 364 USERPAT1 . . .
sEEDmLqa.AVTmSleTv
H2R2I4_PANTR 348 364 USERPAT1 . . .
sEEDmLqa.AVTmSleTv
H2R2I4_PANTR 348 364 USERPAT1 . . .
sEEDmLqa.AVTmSleTv

H2ZYR3_LATCH 109 126 USERPAT1 . . .
tDEEqAisrVLEaSiaEn
H2ZYR3_LATCH 109 126 USERPAT1 . . .
tDEEqAisrVLEaSiaEn

H3BDY2_LATCH 718 734 USERPAT1 . . .
sEEElLaa.VLEmSkrEt
H3BDY2_LATCH 718 734 USERPAT1 . . .
sEEElLaa.VLEmSkrEt
H3BDY2_LATCH 718 734 USERPAT1 . . .
sEEElLaa.VLEmSkrEt
H3BDY2_LATCH 718 734 USERPAT1 . . .
sEEElLaa.VLEmSkrEt

H3CKN5_TETNG 123 140 USERPAT1 . . .
tDEEqAisrVLEaSiaEn
H3CKN5_TETNG 123 140 USERPAT1 . . .
tDEEqAisrVLEaSiaEn

H7C491_HUMAN 11 27 USERPAT1 . . .
sEDEaLqr.ALEmSlaEt

H9FUL0_MACMU 334 350 USERPAT1 . . .
gEEDmLqa.AVTmSleTv
H9FUL0_MACMU 334 350 USERPAT1 . . .
gEEDmLqa.AVTmSleTv
H9FUL0_MACMU 334 350 USERPAT1 . . .
gEEDmLqa.AVTmSleTv

H9H469_MACMU 279 295 USERPAT1 . . .
gEEDmLqa.AVTmSleTv
H9H469_MACMU 279 295 USERPAT1 . . .
gEEDmLqa.AVTmSleTv
H9H469_MACMU 279 295 USERPAT1 . . .

gEEDmLqa.AVTmSleTv

H9H470_MACMU 319 335 USERPAT1 . . .
gEEDmLqa.AVTmSleTv
H9H470_MACMU 319 335 USERPAT1 . . .
gEEDmLqa.AVTmSleTv
H9H470_MACMU 319 335 USERPAT1 . . .
gEEDmLqa.AVTmSleTv

H9KUV4_CANLF 706 722 USERPAT1 . . .
sEEElLaa.VLEmSkrEa
H9KUV4_CANLF 706 722 USERPAT1 . . .
sEEElLaa.VLEmSkrEa
H9KUV4_CANLF 706 722 USERPAT1 . . .
sEEElLaa.VLEmSkrEa

I1IGT8_BRADI 72 88 USERPAT1 . . .
eEDEqLar.ALHeSlnTg
I1IGT8_BRADI 72 88 USERPAT1 . . .
eEDEqLar.ALHeSlnTg

I3KJB2_ORENI 687 703 USERPAT1 . . .
nDDEmLaa.VLEiSrqEa
I3KJB2_ORENI 687 703 USERPAT1 . . .
nDDEmLaa.VLEiSrqEa
I3KJB2_ORENI 687 703 USERPAT1 . . .
nDDEmLaa.VLEiSrqEa

I3KJB3_ORENI 678 694 USERPAT1 . . .
nDDEmLaa.VLEiSrqEa
I3KJB3_ORENI 678 694 USERPAT1 . . .
nDDEmLaa.VLEiSrqEa
I3KJB3_ORENI 678 694 USERPAT1 . . .
nDDEmLaa.VLEiSrqEa

I3L8M9_PIG 123 140 USERPAT1 . . .
tDEEqAisrVLEaSiaEn
I3L8M9_PIG 123 140 USERPAT1 . . .
tDEEqAisrVLEaSiaEn

I3LEV3_PIG 705 721 USERPAT1 . . .
sEEElLaa.VLEiSkrEa
I3LEV3_PIG 705 721 USERPAT1 . . .
sEEElLaa.VLEiSkrEa
I3LEV3_PIG 705 721 USERPAT1 . . .
sEEElLaa.VLEiSkrEa

I3LU36_PIG 123 140 USERPAT1 . . .
tDEEqAisrVLEaSiaEn
I3LU36_PIG 123 140 USERPAT1 . . .
tDEEqAisrVLEaSiaEn

I3MNV2_ICTTR 701 717 USERPAT1 . . .
sEEElLaa.VLEiSkrEa

I3MNV2_ICTTR 701 717 USERPAT1 . . .
sEEElLaa.VLEiSkrEa
I3MNV2_ICTTR 701 717 USERPAT1 . . .
sEEElLaa.VLEiSkrEa

I3MQ41_ICTTR 315 331 USERPAT1 . . .
sEEDmLqa.AVTmSleTv
I3MQ41_ICTTR 315 331 USERPAT1 . . .
sEEDmLqa.AVTmSleTv
I3MQ41_ICTTR 315 331 USERPAT1 . . .
sEEDmLqa.AVTmSleTv

J9P3Q0_CANLF 123 140 USERPAT1 . . .
tDEEqAisrVLEaSiaEn
J9P3Q0_CANLF 123 140 USERPAT1 . . .
tDEEqAisrVLEaSiaEn

J9PA48_CANLF 123 140 USERPAT1 . . .
tDEEqAisrVLEaSiaEn
J9PA48_CANLF 123 140 USERPAT1 . . .
tDEEqAisrVLEaSiaEn

K0KGU5_WICCF 257 273 USERPAT1 . . .
dDDEdLkk.ALElSlrEs
K0KGU5_WICCF 257 273 USERPAT1 . . .
dDDEdLkk.ALElSlrEs

K1P5Q1_CRAGI 964 980 USERPAT1 . . .
gDDEdLkr.ALElSlqEd
K1P5Q1_CRAGI 964 980 USERPAT1 . . .
gDDEdLkr.ALElSlqEd
K1P5Q1_CRAGI 964 980 USERPAT1 . . .
gDDEdLkr.ALElSlqEd

K7EV91_PONAB 302 318 USERPAT1 . . .
sEEDmLqa.AVTmSleTv
K7EV91_PONAB 302 318 USERPAT1 . . .
sEEDmLqa.AVTmSleTv
K7EV91_PONAB 302 318 USERPAT1 . . .
sEEDmLqa.AVTmSleTv

K7FJG9_PELSI 146 163 USERPAT1 . . .
tDEEqAisrVLEaSiaEn
K7FJG9_PELSI 146 163 USERPAT1 . . .
tDEEqAisrVLEaSiaEn

K7FQ01_PELSI 715 731 USERPAT1 . . .
sEDElLaa.VLEiSkrEs
K7FQ01_PELSI 715 731 USERPAT1 . . .
sEDElLaa.VLEiSkrEs
K7FQ01_PELSI 715 731 USERPAT1 . . .
sEDElLaa.VLEiSkrEs

L5JMJ6_PTEAL 128 145 USERPAT1 . . .

tDEEqAisrVLEaSiaEn
L5JMJ6_PTEAL 128 145 USERPAT1 . . .
tDEEqAisrVLEaSiaEn

L5JQB9_PTEAL 335 351 USERPAT1 . . .
sEEDmLqa.AVTmSleTv
L5JQB9_PTEAL 335 351 USERPAT1 . . .
sEEDmLqa.AVTmSleTv
L5JQB9_PTEAL 335 351 USERPAT1 . . .
sEEDmLqa.AVTmSleTv

L5L1R3_PTEAL 671 687 USERPAT1 . . .
sEEElLaa.VLEiSkrEa
L5L1R3_PTEAL 671 687 USERPAT1 . . .
sEEElLaa.VLEiSkrEa
L5L1R3_PTEAL 671 687 USERPAT1 . . .
sEEElLaa.VLEiSkrEa

M2N5Y0_BAUPA 1236 1252 USERPAT1 . . .
tEDEdLqk.ALElSmqGh

M3VUA8_FELCA 123 140 USERPAT1 . . .
tDEEqAisrVLEaSiaEn
M3VUA8_FELCA 123 140 USERPAT1 . . .
tDEEqAisrVLEaSiaEn

M3VYE7_FELCA 705 721 USERPAT1 . . .
sEEElLaa.VLEiSkrEa
M3VYE7_FELCA 705 721 USERPAT1 . . .
sEEElLaa.VLEiSkrEa
M3VYE7_FELCA 705 721 USERPAT1 . . .
sEEElLaa.VLEiSkrEa

M3WT23_FELCA 325 341 USERPAT1 . . .
sEEDmLqa.AVTmSleVv
M3WT23_FELCA 325 341 USERPAT1 . . .
sEEDmLqa.AVTmSleVv
M3WT23_FELCA 325 341 USERPAT1 . . .
sEEDmLqa.AVTmSleVv

M3YH27_MUSPF 127 144 USERPAT1 . . .
tDEEqAisrVLEaSiaEn
M3YH27_MUSPF 127 144 USERPAT1 . . .
tDEEqAisrVLEaSiaEn

M3YYM3_MUSPF 705 721 USERPAT1 . . .
sEEElLaa.VLEmSkrEa
M3YYM3_MUSPF 705 721 USERPAT1 . . .
sEEElLaa.VLEmSkrEa
M3YYM3_MUSPF 705 721 USERPAT1 . . .
sEEElLaa.VLEmSkrEa

M4AKN0_XIPMA 680 696 USERPAT1 . . .
nDDEmLaa.VLEmSrqEa

M4AKN0_XIPMA 680 696 USERPAT1 . . .
nDDEmLaa.VLEmSrqEa
M4AKN0_XIPMA 680 696 USERPAT1 . . .
nDDEmLaa.VLEmSrqEa

M7BB78_CHEMY 715 731 USERPAT1 . . .
sEDElLaa.VLEiSkrEa
M7BB78_CHEMY 715 731 USERPAT1 . . .
sEDElLaa.VLEiSkrEa
M7BB78_CHEMY 715 731 USERPAT1 . . .
sEDElLaa.VLEiSkrEa

Q06AV3_PIG 327 343 USERPAT1 . . .
sEEDmLqa.AVTmSleTv
Q06AV3_PIG 327 343 USERPAT1 . . .
sEEDmLqa.AVTmSleTv
Q06AV3_PIG 327 343 USERPAT1 . . .
sEEDmLqa.AVTmSleTv

Q0P3R6_XENLA 197 213 USERPAT1 . . .
tEEEaLqr.ALEmSlsEs

Q28BY1_XENTR 122 139 USERPAT1 . . .
tDEEqAisrVLEaSiaEn
Q28BY1_XENTR 122 139 USERPAT1 . . .
tDEEqAisrVLEaSiaEn

Q6BZF2_DEBHA 184 200 USERPAT1 . . .
dDDDeMqk.ALEmSriTa
Q6BZF2_DEBHA 184 200 USERPAT1 . . .
dDDDeMqk.ALEmSriTa

Q6C7S0_YARLI 628 645 USERPAT1 . . .
eDEEeAlarALEaSllEa
Q6C7S0_YARLI 628 645 USERPAT1 . . .
eDEEeAlarALEaSllEa
Q6C7S0_YARLI 628 645 USERPAT1 . . .
eDEEeAlarALEaSllEa
Q6C7S0_YARLI 681 697 USERPAT1 . . .
eEDEdLky.ALElSlaEe
Q6C7S0_YARLI 681 697 USERPAT1 . . .
eEDEdLky.ALElSlaEe
Q6C7S0_YARLI 681 697 USERPAT1 . . .
eEDEdLky.ALElSlaEe

Q96B65_HUMAN 123 140 USERPAT1 . . .
tDEEqAisrVLEaSiaEn
Q96B65_HUMAN 123 140 USERPAT1 . . .
tDEEqAisrVLEaSiaEn

R0L3N1_ANAPL 715 731 USERPAT1 . . .
sEDElLaa.VLEiSkrEa
R0L3N1_ANAPL 715 731 USERPAT1 . . .
sEDElLaa.VLEiSkrEa

```
R0L3N1_ANAPL     715      731      USERPAT1 .        .        .
sEDElLaa.VLEiSkrEa

R9PXQ8_CHICK     714      730      USERPAT1 .        .        .
sEDElLaa.VLEiSkrEa
R9PXQ8_CHICK     714      730      USERPAT1 .        .        .
sEDElLaa.VLEiSkrEa
R9PXQ8_CHICK     714      730      USERPAT1 .        .        .
sEDElLaa.VLEiSkrEa

S4R6R4_PETMA     648      664      USERPAT1 .        .        .
tDEDqLkr.ALNlSkqVr
S4R6R4_PETMA     648      664      USERPAT1 .        .        .
tDEDqLkr.ALNlSkqVr

S8AAA9_DACHA     1413     1429     USERPAT1 .        .        .
tDDDdLqr.VLEmSmkEe
S8AAA9_DACHA     1413     1429     USERPAT1 .        .        .
tDDDdLqr.VLEmSmkEe
S8AAA9_DACHA     1413     1429     USERPAT1 .        .        .
tDDDdLqr.VLEmSmkEe

U3IEF8_ANAPP     2        18       USERPAT1 .        .        .
sEDElLaa.VLEiSkrEa
U3IEF8_ANAPP     2        18       USERPAT1 .        .        .
sEDElLaa.VLEiSkrEa
U3IEF8_ANAPP     2        18       USERPAT1 .        .        .
sEDElLaa.VLEiSkrEa

U3JN86_FICAL     714      730      USERPAT1 .        .        .
sEDElLaa.VLEiSkrEa
U3JN86_FICAL     714      730      USERPAT1 .        .        .
sEDElLaa.VLEiSkrEa
U3JN86_FICAL     714      730      USERPAT1 .        .        .
sEDElLaa.VLEiSkrEa

UBP25_HUMAN      123      140      USERPAT1 .        .        .
tDEEqAisrVLEaSiaEn
UBP25_HUMAN      123      140      USERPAT1 .        .        .
tDEEqAisrVLEaSiaEn

UBP25_MOUSE      123      140      USERPAT1 .        .        .
tDEEqAisrVLEaSiaEn
UBP25_MOUSE      123      140      USERPAT1 .        .        .
tDEEqAisrVLEaSiaEn

UBP37_BOVIN      706      722      USERPAT1 .        .        .
sEEElLaa.VLEiSkrEa
UBP37_BOVIN      706      722      USERPAT1 .        .        .
sEEElLaa.VLEiSkrEa
UBP37_BOVIN      706      722      USERPAT1 .        .        .
sEEElLaa.VLEiSkrEa

UBP37_CANLF      706      722      USERPAT1 .        .        .
```

sEEElLaa.VLEmSkrEa
UBP37_CANLF 706 722 USERPAT1 . . .
sEEElLaa.VLEmSkrEa
UBP37_CANLF 706 722 USERPAT1 . . .
sEEElLaa.VLEmSkrEa

UBP37_CHICK 712 728 USERPAT1 . . .
sEDElLaa.VLEiSkrEa
UBP37_CHICK 712 728 USERPAT1 . . .
sEDElLaa.VLEiSkrEa
UBP37_CHICK 712 728 USERPAT1 . . .
sEDElLaa.VLEiSkrEa

UBP37_DANRE 672 688 USERPAT1 . . .
nDEEmLaa.VLEmSrhDt
UBP37_DANRE 672 688 USERPAT1 . . .
nDEEmLaa.VLEmSrhDt
UBP37_DANRE 672 688 USERPAT1 . . .
nDEEmLaa.VLEmSrhDt

UBP37_HUMAN 704 720 USERPAT1 . . .
sEEElLaa.VLEiSkrDa
UBP37_HUMAN 704 720 USERPAT1 . . .
sEEElLaa.VLEiSkrDa

UBP37_MOUSE 704 720 USERPAT1 . . .
sEEEvLaa.VLEiSrrEa
UBP37_MOUSE 704 720 USERPAT1 . . .
sEEEvLaa.VLEiSrrEa
UBP37_MOUSE 704 720 USERPAT1 . . .
sEEEvLaa.VLEiSrrEa

UBP37_PIG 707 723 USERPAT1 . . .
sEEElLaa.VLEiSkrEa
UBP37_PIG 707 723 USERPAT1 . . .
sEEElLaa.VLEiSkrEa
UBP37_PIG 707 723 USERPAT1 . . .
sEEElLaa.VLEiSkrEa

V7CRH6_PHAVU 71 87 USERPAT1 . . .
eEDEqLar.ALEeSlnVe
V7CRH6_PHAVU 71 87 USERPAT1 . . .
eEDEqLar.ALEeSlnVe

V7CTT5_PHAVU 75 91 USERPAT1 . . .
eEDEqLar.ALEeSlnVe
V7CTT5_PHAVU 75 91 USERPAT1 . . .
eEDEqLar.ALEeSlnVe

W2LJ66_PHYPR 330 346 USERPAT1 . . .
dEDEdIrk.ALElSlqTa

W2RCY5_PHYPN 330 346 USERPAT1 . . .
dEDEdIrk.ALElSlqTa

W5KFG5_ASTMX 674 690 USERPAT1 . . .
nDDEmLaa.VLEmSrqEa
W5KFG5_ASTMX 674 690 USERPAT1 . . .
nDDEmLaa.VLEmSrqEa
W5KFG5_ASTMX 674 690 USERPAT1 . . .
nDDEmLaa.VLEmSrqEa

W5MR55_LEPOC 676 692 USERPAT1 . . .
nDEEmLaa.VLEmSrqEn
W5MR55_LEPOC 676 692 USERPAT1 . . .
nDEEmLaa.VLEmSrqEn
W5MR55_LEPOC 676 692 USERPAT1 . . .
nDEEmLaa.VLEmSrqEn

W5PT81_SHEEP 346 362 USERPAT1 . . .
sEEDmLqa.AVTmSleTv
W5PT81_SHEEP 346 362 USERPAT1 . . .
sEEDmLqa.AVTmSleTv
W5PT81_SHEEP 346 362 USERPAT1 . . .
sEEDmLqa.AVTmSleTv

W5Q3D4_SHEEP 117 134 USERPAT1 . . .
tDEEqAisrVLEaSiaEn
W5Q3D4_SHEEP 117 134 USERPAT1 . . .
tDEEqAisrVLEaSiaEn

W5Q3D6_SHEEP 117 134 USERPAT1 . . .
tDEEqAisrVLEaSiaEn
W5Q3D6_SHEEP 117 134 USERPAT1 . . .
tDEEqAisrVLEaSiaEn

W5QEA5_SHEEP 706 722 USERPAT1 . . .
sEEElLaa.VLEiSkrEa
W5QEA5_SHEEP 706 722 USERPAT1 . . .
sEEElLaa.VLEiSkrEa
W5QEA5_SHEEP 706 722 USERPAT1 . . .
sEEElLaa.VLEiSkrEa

Expasy is operated by the SIB Swiss Institute of Bioinformatics |
Terms of Use
Back to the top
