# Supplementary material for: Ubiquitin Interacting Motifs: Duality Between Structured and Disordered Motifs
Source: Front Mol Biosci. 2021 Jun 28;8:676235. doi: 10.3389/fmolb.2021.676235 (PMC8273247; doi:10.3389/fmolb.2021.676235)
Supplement: Supplementary file 2 [file Table1.DOCX]

**
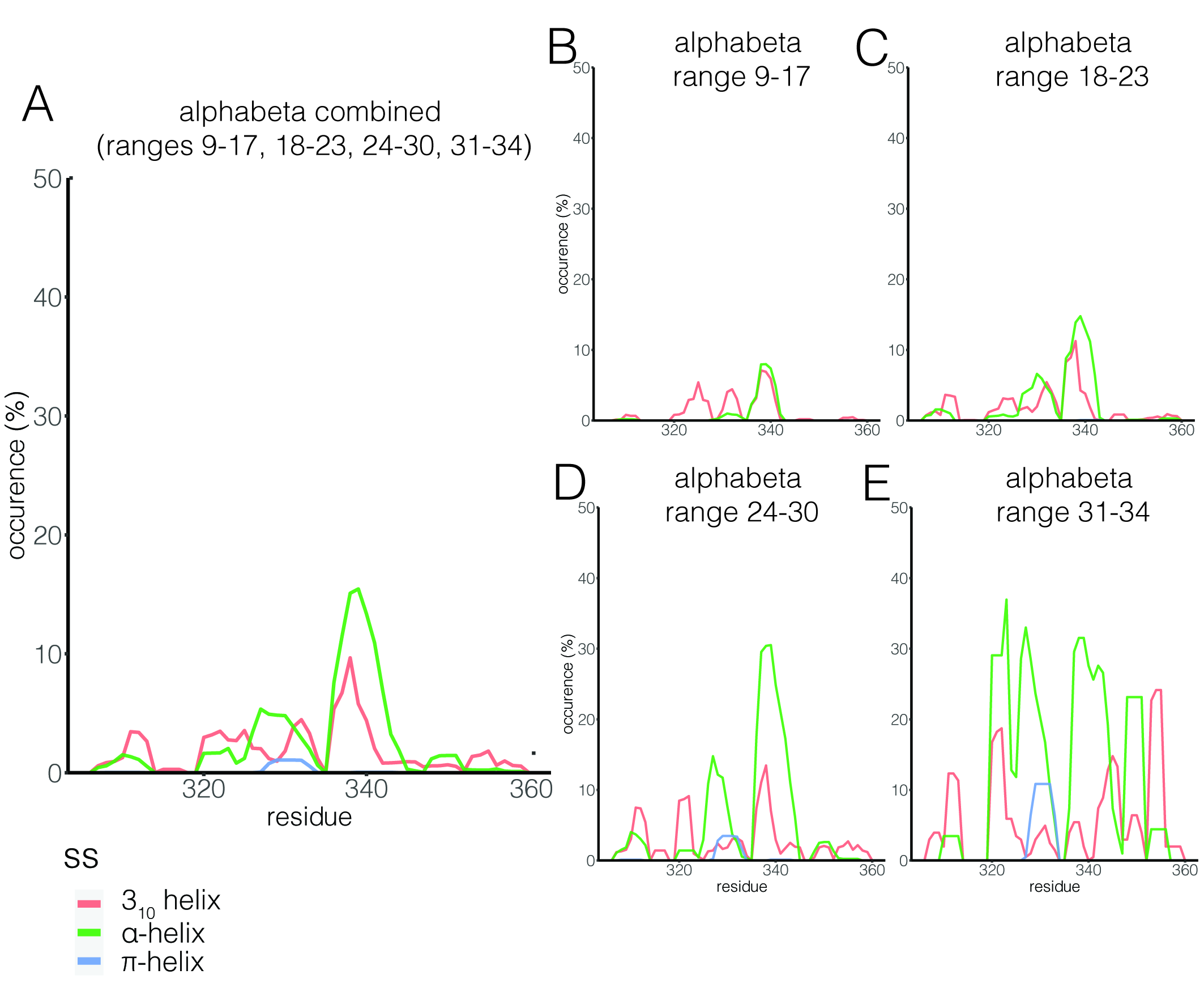
**

**Figure S1.** **UIM3 of AT-3_306-361_ assumes transient helical conformations in the unbound ensemble in solution.** The line plots show the percentage of AT-3_306-361_ structures in which the residues assume helical conformations (3_10_ helix, α-helix, π-helix) according to the DSSP algorithm. We calculated the occurrence of helical conformations on subsets of structures of AT-3_306-361_ from the CHARMM22*_2_ metadynamics with alphabeta values in the ranges of 9-17, 18-23, 24-30, and 31-34, both combined (A) and separately (B-E, respectively). We observe that the N-terminal region of UIM3 (residues 336-344) has the highest occurrence of transient helical conformations in the ensemble. We observe formation of helical structures with lower occurrence also in other tracts of AT-3_306-361_, as around residues 320-334 and to a minor extent around residues 348-351.
