# Supplementary material for: Ubiquitin Interacting Motifs: Duality Between Structured and Disordered Motifs
Source: Front Mol Biosci. 2021 Jun 28;8:676235. doi: 10.3389/fmolb.2021.676235 (PMC8273247; doi:10.3389/fmolb.2021.676235)
Supplement: Supplementary file 3 [file Table2.DOCX]

**
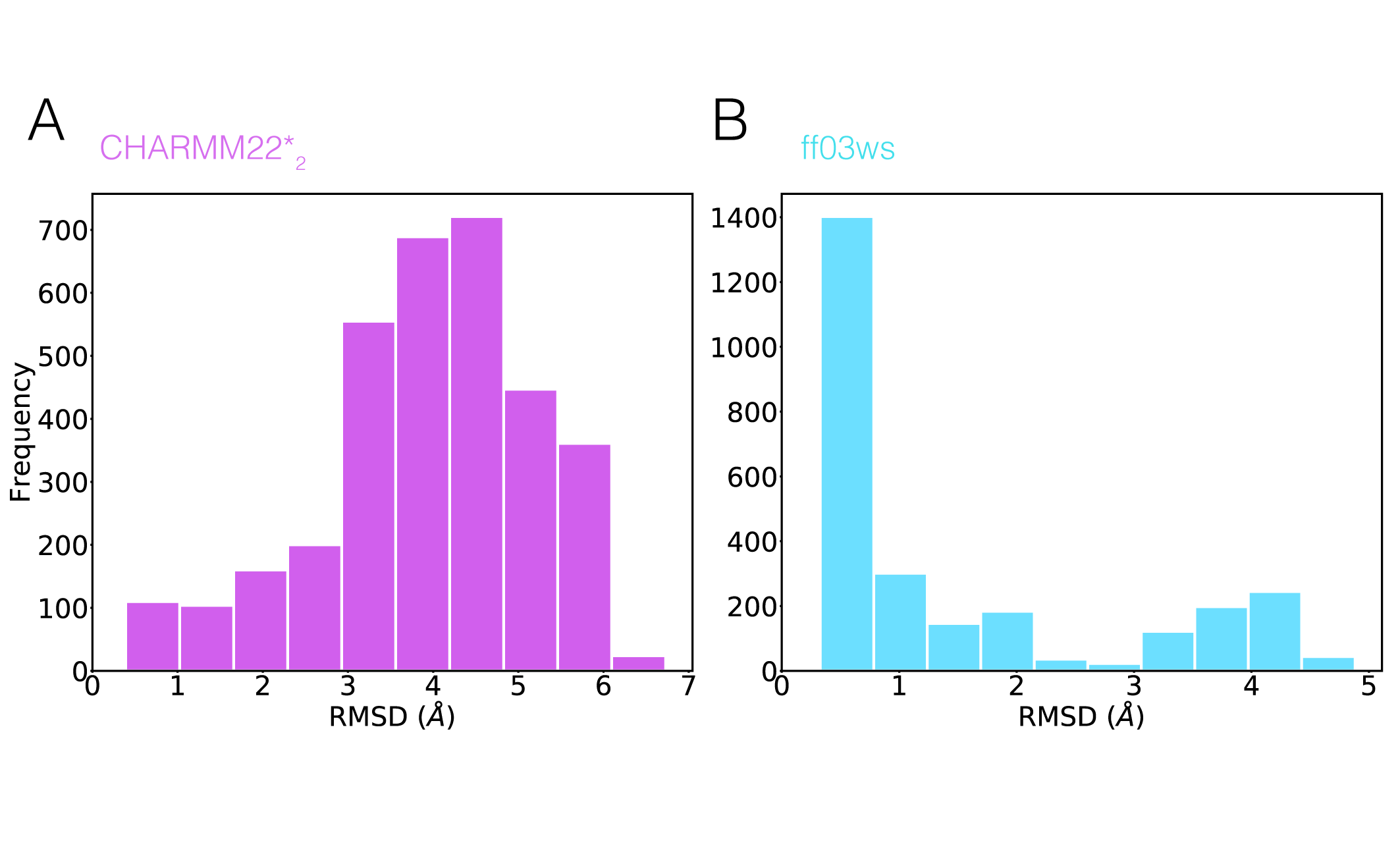
**

**Figure S2. UIM3 of AT-3_306-361_ assumes helical and disordered conformations in the free ensembles.** The plots show the RMSD calculations of UIM3 of AT-3_306-361_ on the replica at 304 K from the REMD simulations with A) CHARMM22*_2_ (pink) and B) ff03ws (blue), using as a reference the starting structures in which the UIM region is modeled as an α-helix.
