# Supplementary figures and images for: Ubiquitin Interacting Motifs: Duality Between Structured and Disordered Motifs
Source: Front Mol Biosci. 2021 Jun 28;8:676235. doi: 10.3389/fmolb.2021.676235 (PMC8273247; doi:10.3389/fmolb.2021.676235)

**
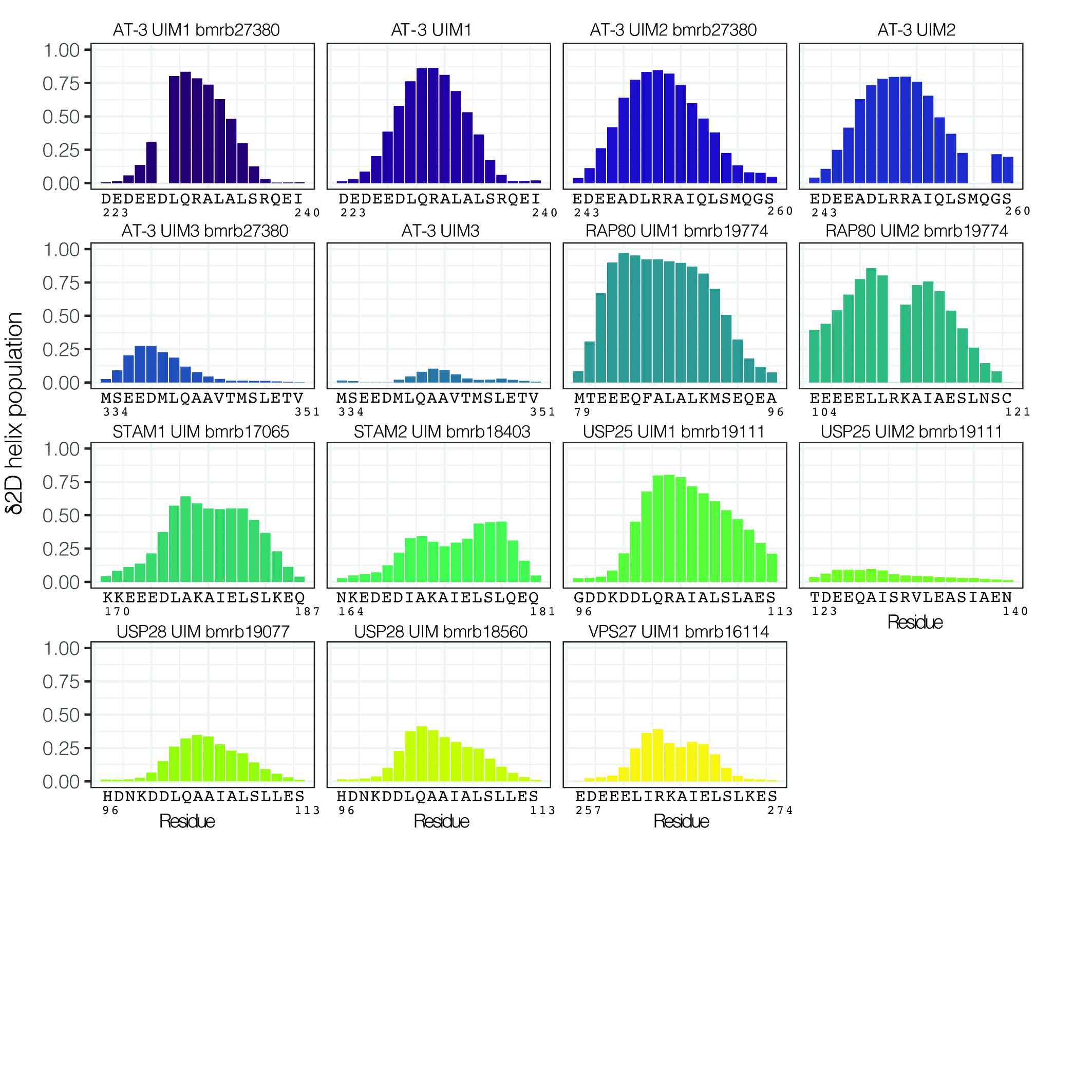
**

**Figure S3.** Helical content for UIMs predicted from experimental NMR chemical shifts by δ2D.

Supplement: Supplementary file 4 [file Table3.DOCX]
