# Supplementary material for: Ubiquitin Interacting Motifs: Duality Between Structured and Disordered Motifs
Source: Front Mol Biosci. 2021 Jun 28;8:676235. doi: 10.3389/fmolb.2021.676235 (PMC8273247; doi:10.3389/fmolb.2021.676235)
Supplement: Supplementary file 5 [file Table4.DOCX]

**
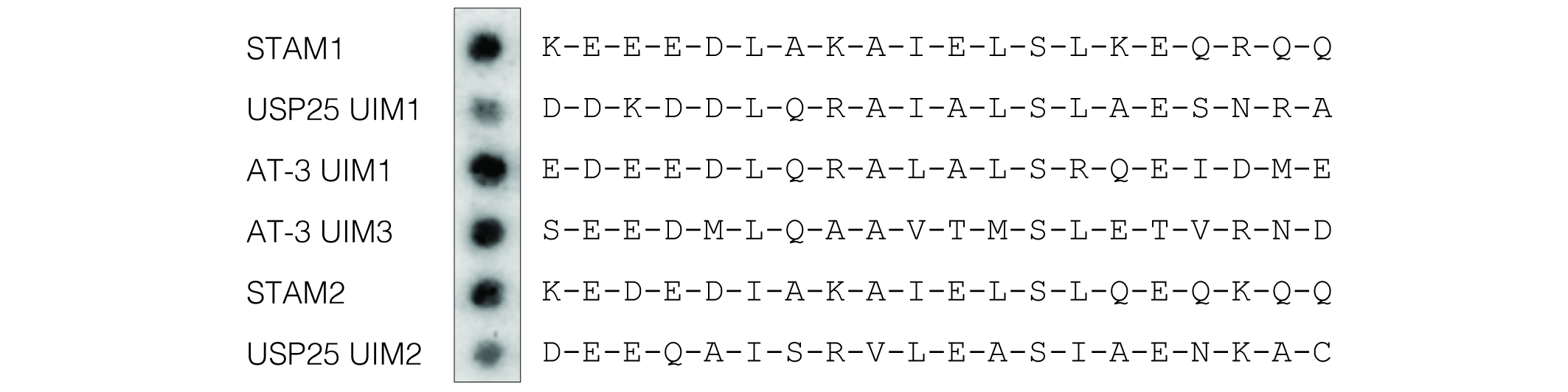
**

**Figure S4. Ub interacts with UIM3 *in vitro***. Peptide array of 20-mer peptides covering UIMs of STAM1, STAM2, USP25, and AT-3 incubated with His-Ub. The peptide/protein interaction is retrieved with anti-his antibody. The spotted peptide sequence is indicated on the right.
